# Comparative Analysis Reveals Dynamic Changes in miRNAs and Their Targets and Expression during Somatic Embryogenesis in Longan (*Dimocarpus longan* Lour.)

**DOI:** 10.1371/journal.pone.0060337

**Published:** 2013-04-11

**Authors:** Yuling Lin, Zhongxiong Lai

**Affiliations:** Institute of Horticultural Biotechnology, Fujian Agriculture and Forestry, Fuzhou, Fujian, China; Wuhan University, China

## Abstract

Somatic embryogenesis (SE), which resembles zygotic embryogenesis, is an essential component of the process of plant cell differentiation and embryo development. Although microRNAs (miRNAs) are important regulators of many plant develop- mental processes, their roles in SE have not been thoroughly investigated. In this study, we used deep-sequencing, computational, and qPCR methods to identify, profile, and describe conserved and novel miRNAs involved in longan (*Dimocarpus longan*) SE. A total of 643 conserved and 29 novel miRNAs (including star strands) from more than 169 miRNA families were identified in longan embryogenic tissue using Solexa sequencing. By combining computational and degradome sequencing approaches, we were able to predict 2063 targets of 272 miRNAs and verify 862 targets of 181 miRNAs. Target annotation revealed that the putative targets were involved in a broad variety of biological processes, including plant metabolism, signal transduction, and stimulus response. Analysis of stage- and tissue-specific expressions of 20 conserved and 4 novel miRNAs indicated their possible roles in longan SE. These miRNAs were *dlo-miR156* family members and dlo-miR166c* associated with early embryonic culture developmental stages; *dlo-miR26*, *dlo-miR160a*, and families *dlo-miR159*, *dlo-miR390,* and *dlo-miR398b* related to heart-shaped and torpedo- shaped embryo formation; *dlo-miR4a, dlo-miR24, dlo-miR167a*, dlo-miR168a*, *dlo-miR397a*, *dlo-miR398b.1*, *dlo-miR398b.2*, *dlo-miR808* and *dlo-miR5077* involved in cotyledonary embryonic development; and *dlo-miR17* and dlo-miR2089*-1 that have regulatory roles during longan SE. In addition, *dlo-miR167a*, *dlo-miR808*, and *dlo-miR5077* may be required for mature embryo formation. This study is the first reported investigation of longan SE involving large-scale cloning, characterization, and expression profiling of miRNAs and their targets. The reported results contribute to our knowledge of somatic embryo miRNAs and provide insights into miRNA biogenesis and expression in plant somatic embryo development.

## Introduction

Longan (*Dimocarpus longan* Lour.) is a tropical/subtropical fruit tree in the family Sapindaceae. In this species, embryo development status strongly influences seed size, fruit quality, fruit set, and yield. To improve longan fruit quality and yield, researchers have consequently focused much attention on mechanisms of longan embryonic development. Advances have been limited, however, primarily because of the extreme genetic heterozygosity exhibited by this species and the difficulty of sampling the early embryos. Although the longan somatic embryogenesis (SE) system has been widely used as a model system for investigating *in vitro* and *in vivo* regulation of embryogenesis in woody plants, most studies of longan SE have focused on a few selected aspects related to cell biology, molecular biology, and proteomics [1]–[5]. There are no reported studies devoted to identification and expression of microRNAs (miRNAs)–an important group of plant regulators–during longan SE.

miRNAs, a class of single-stranded noncoding RNAs that are 18–25 nt in length, negatively regulate gene expression by cleaving targeted mRNAs and repressing translation [6]. There is increasing evidence that plant miRNAs play important roles in various processes, including leaf morphogenesis and polarization, lateral root formation, floral induction, meristem boundary formation, floral organ identity, and reproduction [7]–[10]. SE, which resembles zygotic embryogenesis, is strongly associated with plant cell differentiation and embryo development–a process during which many specific miRNAs are undoubtedly expressed [11], [12]. Several studies have accordingly shown that miRNA-mediated repression of target transcripts plays a role in embryogenesis [13], [14] and that certain miRNAs may function at specific developmental stages [11], [15]–[18]. In *Arabidopsis*, miRNAs enable pattern formation during embryogenesis [14] and regulate the timing of embryo maturation by repressing several key targets [13]. *AGO1*, which is involved in miRNA function, is specifically expressed during carrot SE [19], and *ptAGO9L* as well as miRNAs such as *Pta-miR166* and *Pta-miR167* exhibit spatiotemporally variable expression in loblolly pine zygotic embryos and female gametophytes [18]. These observations suggest that a miRNA expression controlling system is required for SE in these species. The regulatory role of miRNAs during plant embryogenesis is still not well understood, however, and few relevant studies have been conducted in non-model plants. No studies have attempted to detect miRNAs in longan.

In this study, we used Solexa sequencing and an integrated bioinformatics analysis approach to clone and identify conserved and novel miRNAs in longan. We then predicted, identified, and verified longan miRNA targets using prediction analysis, degradome sequencing, and modified RNA ligase-mediated amplification of cDNA ends (RLM-RACE). Finally, we comparatively analyzed expression patterns to reveal miRNA participation in longan SE. The results of this study provide new information regarding miRNA regulatory networks and contribute to our knowledge of the role of miRNAs in longan SE.

## Results

### Categories and Size Distribution of Small RNA Populations in *D. longan*

To identify miRNAs involved in longan SE, a pooled sRNA library was generated from embryogenic cultures and subjected to Solexa sequencing. We obtained 12,554,858 raw reads, comprising 447,851 low-quality and 12,107,007 high-quality reads. After removing low-quality reads and adapter sequences, the remaining 11,645,841 clean reads, which were 18–30 nt long, included 6,553,782 unique sequences. Only 378,143 (5.77%) of the unique sRNA sequences could be mapped to the longan transcriptome (Table 1). Most of the sequences (94.23%) could not be mapped.

**Table 1:** Distribution of small RNAs among different categories in *Dimocarpus longan.*

category	Unique sRNA	Percent(%)	Total sRNA	Percent(%)
miRNA	18,299	0.28	1,208,973	10.38
rRNA	32,362	0.49	342,850	2.94
siRNA	–	–	–	–
snRNA	1,759	0.03	6,220	0.05
snoRNA	1,872	0.03	6,820	0.06
tRNA	5,633	0.09	158,587	1.36
unannotated	6,493,857	99.09	9,922,391	85.20
Total	6,553,782	100	11,645,841	100

The unique sRNAs were then compared against all plant miRNA precursors and mature miRNAs listed in miRBase (miRBase17); 18,299 (0.28%) of the unique sequences were found to be similar to known miRNAs. By performing a BLASTN search against the Rfam database, we then identified small RNAs corresponding to rRNA (0.49%), small nuclear RNA (snRNA; 0.03%), small nucleolar RNA (snoRNA; 0.03%), and tRNA (0.09%) in our unique sRNA dataset (Table 1). Most sRNA unique sequences (6,351,648; 96.92%) could not be annotated, however, which is consistent with results obtained in other plant studies [20]–[23]. The high percentage of unannotated sequences may be due to the limited number of species-specific genomes or ESTs in these databases, such that many potential miRNAs were missing; alternatively, plant sRNAs have not yet been adequately surveyed, leading to incomplete data. The distribution of sRNAs detected in longan is given in Table 1.

**Figure 1:**
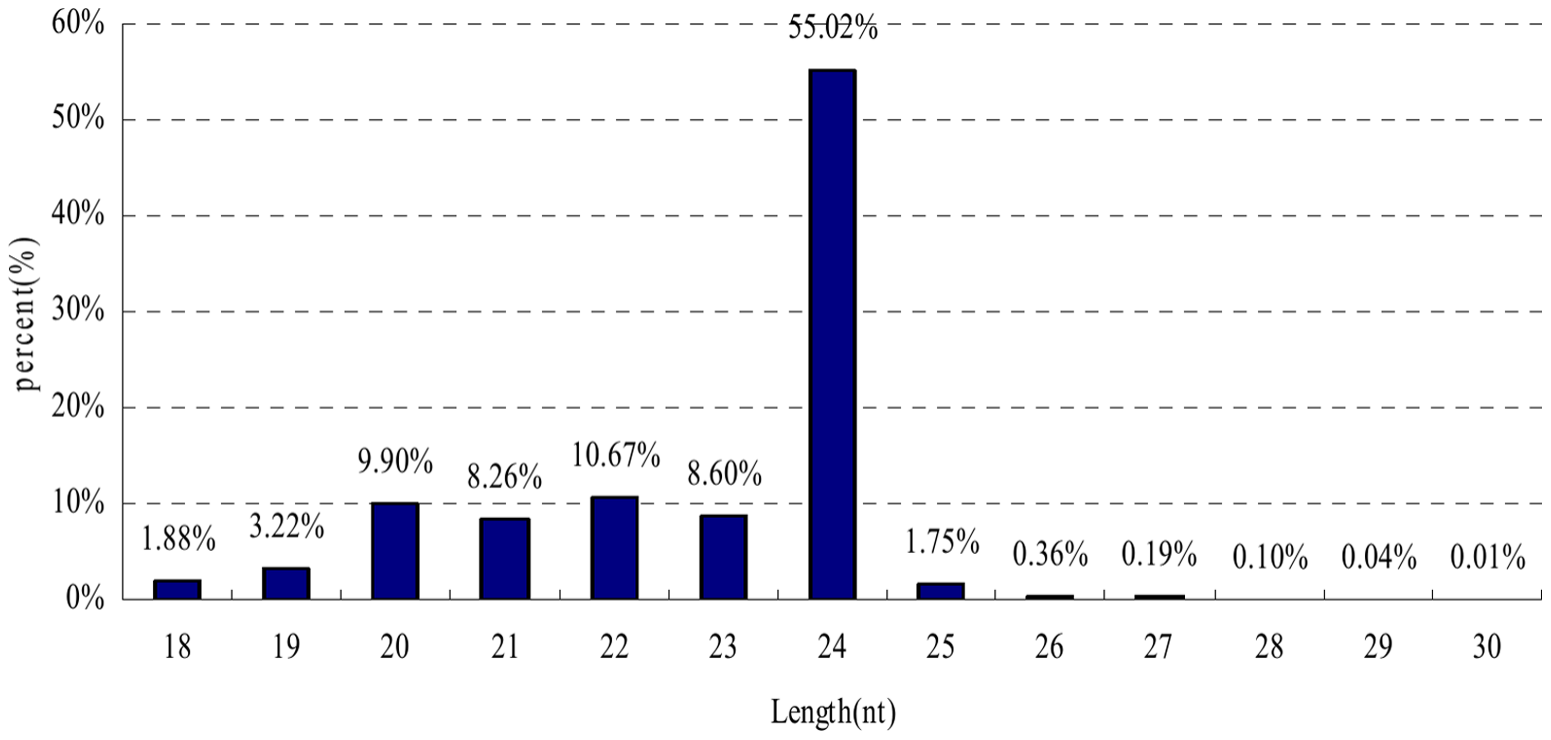
Length distribution of unique sequences in longan.

The size distribution pattern of unique sRNA sequences generated by Solexa sequencing is summarized in Fig. 1. Distributions were uneven. Unique sRNAs were 18–25 nt long, with 24 nt lengths predominating (55.02%), followed by 22 nt (10.67%) and 20 nt (9.90%). The percentage of 24-nt sRNAs was much higher than that of 21 nt sRNAs. This result is consistent with that observed for most angiosperms [21], [23], [24] other than tomato [25], wheat [26], and *Populus* [27], in which the 21-nt length is more prevalent. The 21-nt to 24-nt length ratio is highly variable among plants, indicating the existence of significant differences in sRNA biogenesis pathways among different species [28].

### Identification of Conserved miRNAs in *D. longan*

To identify conserved miRNAs in *D. longan*, unique sRNAs from longan were compared against mature plant miRNAs listed in miRBase17. As a result, 643 miRNAs belonging to more than 169 miRNA families were identified. These miRNAs varied in length from 18 nt to 25 nt. Most miRNAs were 24-nt long (34.07%), with 21 nt (20.2%), 20 nt (12.36%), and 19 nt (10.62%) lengths also heavily represented. A few were 25 nt long, such as *dlo-miR2095* and dlo-miR3436*. After miRNAs with expression levels too low to be analyzed were removed, most miRNA families were represented by only one member. Some miRNA families were represented by multiple sRNAs, including *miR156* (17 members), *miR166* (12 members), *miR159* (8 members), *miR171* (9 members), *miR399* (5 members), *miR441* (6 members), and *miR1520* (6 members); these required further validation, however. Information about known miRNA families detected in longan, including number of members, sizes, and expression, is included in Table S1 in Supporting Information S1.

In addition to mature miRNAs, some miRNAs* with high expression were sequenced, including dlo-miR408* (3727 reads), dlo-miR946a* (1114 reads), dlo-miR 390a* (723 reads), dlo-miR3441* (433 reads), dlo-miR168a* (404 reads), dlo-miR 166c* (346 reads), and dlo-miR1162* (300 reads) (Table S1 in Supporting Information S1). Compared with mature miRNAs, however, lower expression levels were observed during longan SE for most of the miRNAs*. This asymmetrical accumulation of transcripts may be due to preferential loading of the miRNA strand into the silencing complex, where it is protected from degradation; this is in contrast to the miRNA* strand, which is preferentially excluded from the silencing complex and subject to degradation [29].

Most of the miRNA families identified in longan are highly conserved across many plant species. For example, families *dlo-miR156* and *dlo-miR157* were a perfect match to families in *Arabidopsis thaliana*, *Zea mays*, *Vitis vinifera*, and *Populus*, and families *dlo-miR158*–*dlo-miR172* and *dlo-miR393–dlo-miR399* were highly homologous to families from other species. Dicot-specific miRNA families, including *miR158*, *miR161*, *miR163*, and *miR403* [30], [31], were also detected in longan. In addition to conserved miRNAs, 21 non-conserved miRNA families were present in low abundance (2–123 reads) (Table S1 in Supporting Information S1); a few, including *dlo-miR535a* (9232 reads), *dlo-miR894* (6039 reads), *dlo-miR529* (881 reads) and *dlo-miR827* (753 reads), were expressed at high levels. Interestingly, several miRNA families considered to be monocot-specific–*miR437*, *miR443*, *miR444*, *miR445*, *miR528*, *miR1122*, and *miR1858*–were also found in our library (2–173 reads), suggesting that they also exist in dicots.

### Prediction of Potentially Novel Longan miRNAs via Longan Transcriptome and *Populus* Genome Analysis

In addition to conserved miRNAs, 29 potentially novel longan miRNAs were predicted from the remaining unannotated sRNA unique sequences–22 based on the longan transcriptome and 7 based on a *Populus trichocarpa* genome (Table 2). With the exception of *dlo-miR4* and *dlo-miR27*, which were represented by 2 genomic loci each, each novel longan miRNA was associated with a single genomic locus. The lengths of these miRNAs varied from 21 nt to 23 nt; 48.28% were 21 nt long. Based on Mfold calculations, their precursors had negative folding free energies ranging from −18.20 kcal/mol to −65.24 kcal/mol; the average was −40.03 kcal/mol, which was lower than the folding free energies reported for rice (−71.0 kcal/mol) and *Arabidopsis* (−59.5 kcal/mol) [26]. The number of reads obtained for these novel longan miRNAs varied from 5 to 8964; for example, *dlo-miR7* (8964 reads), *dlo-miR17* (1309), *dlo-miR9* (1144 reads), and *dlo-miR14* (609 reads) were highly expressed, while *dlo-miR1* (5 reads) and *dlo-miR18* (6 reads) were expressed at low levels. When nucleotide bias was analyzed, the nucleotide T (58.62%) was most frequent, followed by A (20.69%), C (10.34%), and G (13.79%).

**Table 2:** Longan-specific miRNAs identified from *Dimocarpus longan* transcriptome and populus genome.

miRNA	location	Sequence(5′–3′)	Length(nt)	count	Mfe(kcal/mol)	5′/3′
*dlo-miR1*	Contig325306_Longan-RNA:27∶90:+	TATGTATATATATGTGTAGAT	21	5	−18.2	3
*dlo-miR2*	Contig401887_Longan-RNA:25∶90:−	TTGCTGGTGATGTGGTGGGGTG	22	9	−25.5	5
*dlo-miR3*	Contig424091_Longan-RNA:14∶90:−	AAAGATCTTTAGGTTTTCGT	20	15	−31.6	3
*dlo-miR4a*	Contig569989_Longan-RNA:1∶81:−	ACGGGTTCAAAGGTTGACAGA	21	11	−27.2	3
*dlo-miR4b*	Unigene4793_Longan-RNA:488∶584:+	ACGGGTTCAAAGGTTGACAGA	21	13	−32	3
*dlo-miR5*	Contig629412_Longan-RNA:1∶95:−	TTGAGGAGTGGAAGTCCAGAA	21	17	−19.75	3
*dlo-miR6*	Scaffold39496_Longan-RNA:47∶139:-	TCAAGGGATGAAGATTTTAACT	22	213	−21	3
*dlo-miR7*	Unigene10300_Longan-RNA:116∶231:−	TCATCGGAACACAAGCCCATGC	22	8964	−47.5	3
*dlo-miR8*	Unigene12720_Longan-RNA:16∶197:+	TTGATCAAATGTCCAAGGCTCA	22	611	−61.5	5
*dlo-miR9*	Unigene13717_Longan-RNA:777∶907:−	AGTGAATGATGCGGGAGACAAAT	23	1144	−42.61	3
*dlo-miR10*	Unigene14204_Longan-RNA:50∶162:+	TTCGAGCGCAAATTAATAGGA	21	12	−51.5	3
*dlo-miR11*	Unigene19708_Longan-RNA:14∶99:−	TGATGCTGTAGATGATTCGGA	21	187	−20.7	5
*dlo-miR12*	Unigene3012_Longan-RNA:21∶128:+	TGAAAATGAACTTAAGAGTTGC	22	120	−35.4	3
*dlo-miR13*	Unigene30135_Longan-RNA:22∶153:−	AGAAGTTTTGATCTCGTAAGACA	23	164	−54	5
*dlo-miR14*	Unigene31710_Longan-RNA:49∶145:−	GGAGCGACCTGAGATCACATG	21	609	−49.8	5
*dlo-miR15*	Unigene40670_Longan-RNA:84∶181:+	TGGCTTTAATGAAGACCTGGG	21	10	−37.89	3
*dlo-miR16*	Unigene47226_Longan-RNA:9∶101:−	GGTATGGAAGGATTGGGTGCA	21	57	−35.2	5
*dlo-miR17*	Unigene54607_Longan-RNA:85∶298:+	TGCAGCACAAAATACAGTCTGG	22	1309	−77.6	3
*dlo-miR18*	Unigene56582_Longan-RNA:219∶296:+	TGGGAATTGTGATGACTTACAT	22	6	−21.2	3
*dlo-miR19*	Unigene60375_Longan-RNA:59∶202:−	CGTCATTCTAGTCGGATCATT	21	490	−55.81	5
*dlo-miR20*	Unigene67206_Longan-RNA:105∶270:−	TTGAGGAAGTGAGCAAGAAAT	21	21	−65.24	5
*dlo-miR21*	Unigene8816_Longan-RNA:7∶111:−	GAGGATTGATGGTAGACCTAAG	22	243	−22.8	3
*dlo-miR22*	LG_I:30129469∶30129680:+	AAGTTTAAGAGGGGGTGTTGAA	22	10	−41.5	5
*dlo-miR23*	LG_IV:14080863∶14081082:+	GCTGGAGTAGCTCAGTTGGTT	21	130	−44.8	5
*dlo-miR24*	LG_V:15276189∶15276277:−	TGAATGATTTCGGACCAGGCT	21	122	−40.8	3
*dlo-miR25*	LG_VIII:10042573∶10042866:−	CCGACCTTAGCTCAGTTGGCAGA	23	72	−48.18	3
*dlo-miR26*	LG_XIII:9782450∶9782561:+	TGTGAATGATGCGGGAGATAA	21	14	−40	3
*dlo-miR27a*	LG_II:21395857∶21395930:−	TGGGCGTGCCGGAGTGGTTATC	22	10	−28.6	5
*dlo-miR27b*	LG_VIII:8501904∶8501977:+	TGGGCGTGCCGGAGTGGTTATC	22	10	−32	5

To determine whether these novel miRNAs are conserved across other plant species, their sequences were compared with miRNA sequences of other organisms present in miRBase (miRBase19.0). This analysis revealed that *dlo-miR14*, *dlo-miR25*, and *dlo-miR26* were orthologous to hbr-miR398, ptc-miR6478 [32], and mtr-miR 4414b, respectively, suggesting that these 3 miRNAs, newly identified in longan, are conserved in other plants. In addition to these 3 miRNAs, 11 other potentially novel miRNAs (4 based on longan and 7 based on *P. trichocarpa*) also had matches in the miRBase database. Although very similar to zma-miR156d*, *gma-miR159a*, aly-miR 162a*, *vun-miR164*, zma-miR166c*, aly-miR168a*, aly-miR171c*, aly-miR390a*, *gma-miR390a*, and zma-miR398a*, they were not identical, which raises the question as to whether or not these miRNAs should be classified as known miRNAs. This problem is not unique: Pantaleo et al. [33] found that *miRC9* (a new miRNA) was very similar to *aqc-miR477e*, differing by only 2 mismatches. They classified *miRC9* as a new miRNA not belonging to the *miR477* family, however, because there were 6 mismatches between *miRC9* and *vvi-miR477* and 7 mismatches between *miRC9* and *ptc-miR477*. In our study, the 11 potentially novel miRNAs in longan showed high homology to known conserved miRNAs, and were consequently classified as known miRNAs rather than truly novel ones. As miRNAs are sequenced in an increasing number of species, this situation is expected to arise more frequently, leading to difficulties in miRNA classification.

Furthermore, miRNAs *dlo-miR22* through *dlo-miR27*, which were predicted based on the *P. trichocarpa* genome, cannot be accurately classified as longan- specific miRNAs because they may also be expressed in *P. trichocarpa*. If possible, species-specific miRNA prediction for non-model plants should rely on ESTs or GSSs originating from the studied species. When reference sequences originate from closely-related species, any predicted “novel” miRNAs will not be species-unique, but will instead be conserved across the selected species.

### Prediction and Classification of Targets of Conserved and Non-conserved miRNAs in *D. longan*

To better understand the biological functions of longan miRNAs identified in our study, 2040 targets of 260 conserved miRNAs and 23 targets of 12 novel miRNA families were predicted using previously-described methods (Table S2 in Supporting Information S1).

Homologs of known miRNA targets were predicted for several conserved longan miRNAs, including *SPL10* as a target of *dlo-miR156*, *MYB33* for *dlo-miR159/319*, *NAC* for *dlo-miR164*, *ARF3/ARF8* for *dlo-miR167*, *SCL6* for *dlo-miR171*, *AP2* for *dlo-miR172*, *TIR1* for *dlo-miR393*, *F-box/NF-YC11* for *dlo-miR394*, and *laccase* for *dlo-miR397/408*. In addition, some miRNAs regulated more than one target. For example, while 23.85% (62) of the conserved miRNAs regulated only one target, 103 (39.62%) regulated 2–5 targets, and 19, including *miR2628* (169 targets), *miR2673* (118 targets), *miR854* (119 targets), *miR780* (51 targets), and *miR1442* (48 targets), had at least 20 targets; this suggests that these miRNAs are probably involved in a variety of biological processes during longan SE. Finally, some targets were regulated by multiple miRNAs. Examples include Unigene 62142 (ubiquitin-associated/TS-N domain-containing protein) targeted by both *miR396* and *miR1520*, and Unigene 68246 (GRAS family transcription factor) regulated by *miR171*, *miR952*, *miR1081*, and *miR5078*. The above-predicted targets still required further experimental validation, however.

Based on Gene Ontology (GO) annotations, these potential miRNA targets appear to be involved in a broad variety of biological processes. Their functions could be divided into 5 categories: (1) plant metabolism, including biosynthesis of secondary metabolism, lignin catabolism, ethanol metabolism, and cell metabolism, (2) signal transduction and apoptosis, including calcium, B-cell receptor, TGF-β, and chemokine signaling pathways, (3) biological and abiotic stress processes (as for the NBS-LRR-type disease resistance protein, a target of *miR482)*, (4) developmental processes (the case for *LEC* [Unigene 60033, a target of *miR476*] closely related to embryonic development), and (5) for many longan miRNA targets, genetic information processing, including categories such as spliceosome, ribosome, and RNA degradation, nucleotide excision, mismatch, and base excision repair, and homologous recombination. In addition, predicted targets not only included transcription factor genes (e.g., *AP2*, *NF-YC11*, *SPL*, *MYB*, and *WRKY*), but also novel genes of unknown function. The miRNAs identified in this study may control longan somatic embryo development by regulating the expression of these targets.

### Identification of miRNA Cleavage Targets in *D.longan* Using Degradome Analysis

Bioinformatics analysis alone could not be used to determine whether the predicted miRNA targets were real or false positives. To verify predicted targets of known and novel miRNAs in the longan transcriptome, a high-throughput experimental approach–degradome sequencing–was therefore employed. More than 30 million raw reads were produced by Solexa sequencing. After processing, 72.14% of the resulting clean reads were 20- or 21-nt long; 58.81% (1,198,119) of the unique reads mapped perfectly to the longan transcriptome, indicating the high quality of the degradome library. The reads that mapped to the longan transcriptome were subjected to further analysis.

A total of 843 targets of 178 conserved miRNAs and 19 targets of 3 novel miRNAs (*dlo-miR1*, *dlo-miR2*, and *dlo-miR5*) were identified (Table S3 in Supporting Information S1). Similar to predictions based on sRNA library sequencing, degradome analysis indicated that most of the known miRNAs were capable of regulating multiple targets. These miRNAs included *dlo-miR854* (90 targets), *dlo-miR2673* (37 targets), *dlo-miR1046* (21 targets), *dlo-miR780* (13 targets), *dlo-miR1442* (10 targets), and *dlo-miR2628* (9 targets). The number of targets identified through degradome sequencing was less than that obtained from bioinformatics prediction methods, perhaps because the latter approach could not distinguish genuine targets from false ones. In contrast to known miRNAs, novel miRNAs (i.e., *dlo-miR1, dlo-miR2,* and *dlo-miR5*) appeared to have only a limited number of targets.

In longan, cleavage sites for most miRNAs were usually located in the CDS of their targets. Most targeted mRNAs harbored multiple cleavage sites, a phenomenon also observed in Arabidopsis and rice [34], [35]. For example, the C2H2 (Unigene 11990) mRNA (targeted by *miR157*) was cleaved at nucleotide positions 211, 330, 331, 387, 404, and 1143. Only the site at position 211 was mediated by *miR157,* however. The remaining cleavage sites were split by other sRNAs or by unknown miRNAs, suggesting a complex degradation mechanism for these targets in plants. The cleavage sites in a given transcript may be targeted either by different miRNAs or the same one; the latter is the case with Unigene 67649 (similar to At4g28230), which has 7 cleavage sites (between positions 1411 and 1417) that are all split by *dlo-miR 2673*. This example demonstrates that cleavage does not always take place between nucleotides 10 and 11 of the target transcript, thus providing a new reference for miRNA target prediction.

Because prediction methods cannot determine the authenticity of miRNA targets, experimental confirmation is required. To verify the accuracy of miRNA target prediction in our study, we compared the results of prediction analysis and degradome sequencing. Surprisingly, most of the predicted targets could not be verified by degradome sequencing. This was the case with several highly conserved longan miRNAs (*miR156*, *miR161*, *miR164*, *miR171*, *miR172*, *miR393*, *miR394*, and *miR408*) that were predicted to regulate homologs of known miRNA targets. Degradome sequencing failed to identify these targets, and also failed to identify other predicted targets for the above miRNAs. In addition, targets could not be predicted or verified for some highly conserved miRNAs using either prediction or degradome sequencing methods, although additional non-conserved targets were identified for them instead. This is consistent with the results of a previous study [36] and indicates that some conserved miRNAs had additional “novel” targets. Further analysis revealed that among 2063 predicted targets of 272 longan miRNAs, only 27 predicted targets of 18 miRNAs were verified by the degradome method. For example, 5 predicted targets of *miR2673*, including Unigene 49256 (expressed protein), Unigene 54104 (*GIL1*), Unigene 60784 (similar to At1g73320), Unigene 67649 (similar to At4g28230), and Unigene 67737 (*MYB*), as well as several targets of *dlo-miR780*, *dlo-miR2099*, *dlo-miR5021*, and *dlo-miR5057*, were verified by degradome analysis (Table S4 in Supporting Information S1). There are several possible explanations for the observed discrepancies. For example, it is possible that miRNAs (e.g., *miR161, miR393*, and *miR394*) or targets were not detected because of their low expression levels during longan SE or because they were specific to tissues that had not been analyzed. Another possibility is that the degradome or transcriptome data was incomplete for target analysis. Alternatively, some miRNAs, such as *miR156* and *miR172*, inhibit expression of their target genes (*SBP* and *AP2*), primarily through translational arrest. Our results suggest that a combined prediction and degradome analysis approach is the most effective way to identify miRNA targets in non-model plants.

### Validation of Longan *miR398* Targets Using Modified RLM-RACE

Recent studies have demonstrated that *miR398* plays a key role in plant SE [11], [15], [16]. Although *miR398* influences plant response to oxidative stress by down- regulating Cu/Zn-superoxide dismutase (*CSD*) expression [37], it is not known whether this miRNA’s regulatory role in plant SE also involves *CSD* down-regulation. To verify the nature of predicted *miR398* targets and to investigate possible regulation of *CSD* by *miR398* during longan SE, we performed a psRNATarget analysis and a modified RLM-RACE experiment.

**Figure 2:**
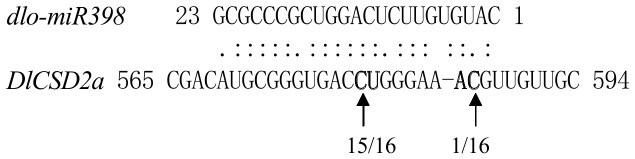
Cleavage site mapping of *miR398* target gene. mRNA sequence of *DlCSD2a* is aligned with *miR398*. Nucleotides flanking the cleavage site are in bold. Numbers indicate the fraction of cloned PCR products terminating at different positions.

The psRNATarget analysis indicated that *dlo-miR398* can target *DlCSD1a* and *DlCSD1b*. This result is consistent with previous studies in *Arabidopsis* and rice [30], [38], [39], indicating that these miRNA binding sites are highly conserved among different plant species. A modified RLM-RACE experiment was then conducted, which verified *DlCSD2a* as a target of *dlo-miR398* (Fig. 2). Tags derived from cleavage of *DlCSD2a* by *dlo-miR398* were predominant, suggesting that *dlo-miR398* plays a major role in *DlCSD2a* regulation during longan SE. On the other hand, the conserved target *DlCSD1a* was not detected, implying that *DlCSD1a* expression is not regulated by *dlo-miR398* during longan SE. These results indicate that *miR398* regulates longan SE primarily through mediation of the target *DlCSD2a.*

### Expression Patterns of Conserved and Novel miRNAs During Longan SE

**Figure 3:**
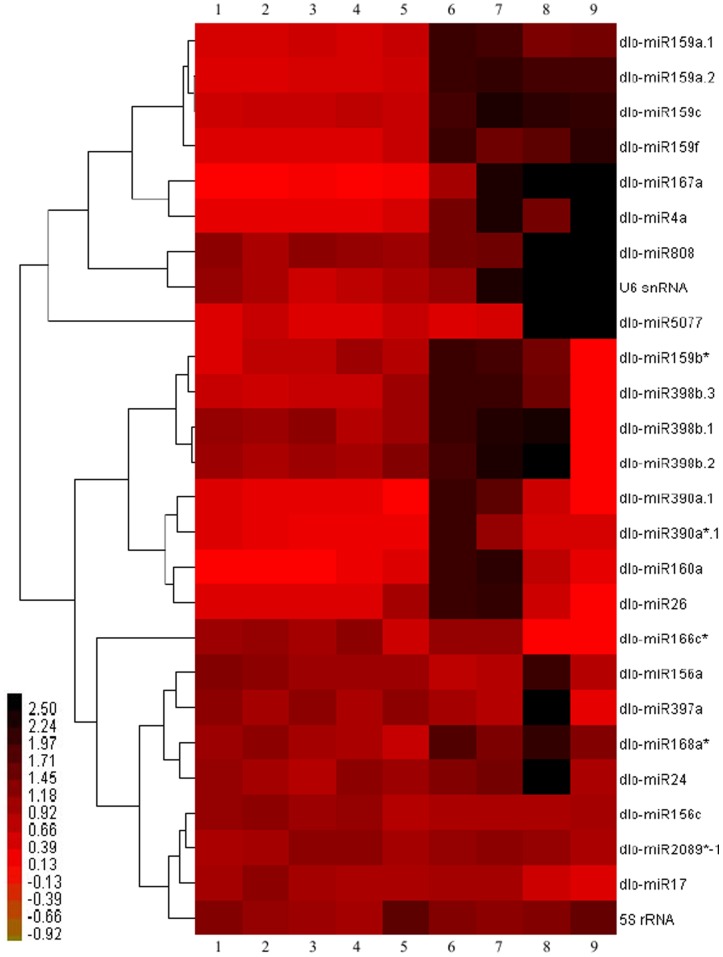
qPCR analysis of relative expressions of known and novel miRNAs, 5S rRNA, and U6 snRNA during longan SE. The bar represents the scale of relative expression levels of miRNAs, and colors indicate relative signal intensities of miRNAs. Each column represents a sample, and each row represents a single miRNA. Samples: 1. friable-embryogenic callus(EC); 2. embryogenic callus II(EC II); 3. incomplete compact pro-embryogenic cultures(ICpEC); 4. compact pro-embryogenic cultures(CpEC); 5. globular embryos(GE); 6. heart-shaped embryos(HE); 7. torpedo- shaped embryos(TE); 8. cotyledonary embryos(CE); 9. mature embryos(ME).

**Figure 4:**
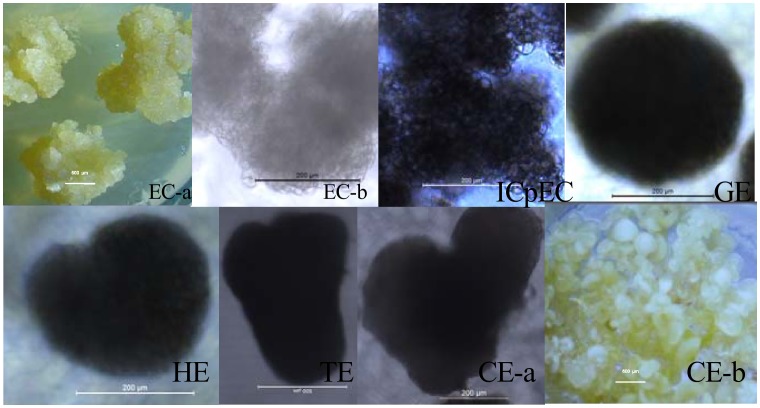
Morphology of embryogenic calli and embryos during the six sequential developmental stages of longan SE. Developmental stages are indicated at the left of each row. The bars in each phenotypic class are indicated at the middle of each image. The morphology of embryogenic cultures (EC-b, ICpEC, GE, HE, TE, CE-a) were observed using an inverted Leica DMIL LED microscope, except for TE(bar = 500 µm), the bars of others are 200 µm; the images of EC-a and CE-b were both obtained under an Leica DFC295, bars, 600 µm; EC, ICpEC, GE, HE, and TE were cultured on MS medium supplemented with 1 mg/L, 0.5 mg/L, 0.1 mg/L, 0.06 mg/L and 0.03 mg/L 2,4-D, respectively; and the CE was cultured on MS medium.

Expression patterns of miRNAs were closely related to their functions. To better understand the role of miRNAs during longan SE, expression patterns of 24 miRNAs (20 conserved and 4 novel), U6 *snRNA*, and *5S rRNA* were analyzed using quantitative real-time PCR (qPCR). The qPCR analysis revealed that all selected miRNAs were expressed at varying levels in different embryogenic tissues, with some preferentially expressed in specific tissues. The miRNAs could be divided into roughly 3 groups (Fig. 3 and Fig. 4).

The first group, consisting of U6 *snRNA* and miRNAs, *dlo-miR159*, *dlo-miR4a*, *dlo-miR808*, and *dlo-miR5077*, displayed ubiquitous but varied expression levels during middle to late stages of longan SE. Different members of the *dlo-miR159* family had contrasting expression patterns, suggesting that each member of a family may have its own function. Expression of *miR159a.1* was high in heart- and torpedo-shaped embryos, but low in other tissues tested, especially in friable-embryogenic calli. *miR159f* was strongly expressed in heart-shaped and mature embryos. *miR159a.2* and *miR159c* had similar expression patterns during longan SE, being expressed weakly during early stages but abundantly in middle and late stages. *dlo-miR4* showed strong expression in torpedo-shaped and mature embryos, moderate expression in globular and heart-shaped embryos, and weak expression elsewhere. *dlo-miR5077* was highly expressed during late stages of longan SE. In addition, *dlo-miR808* and *U6 snRNA* also exhibited a similar expression pattern: unlike the other miRNAs, they were highly expressed during both early and late developmental stages, suggesting a regulatory role in morphogenesis during longan early and late SE.

In the second category, dlo-miR159b*, *dlo-miR160a*, *dlo-miR167a, dlo-miR390*, *dlo-miR398b*, and *dlo-miR26* exhibited tissue-specific expression, indicating that they may have stage-specific functions during longan SE. Among these, dlo-miR159b* expression patterns were similar to those of the *miR398b* family (b.1, b.2, and b.3): they were all highly expressed in heart-shaped, torpedo-shaped, and cotyledonary embryos, but barely detectable in mature embryos. In an earlier study in oranges [16], *miR398* also exhibited high expression in globular and cotyledonary embryos, suggesting that its function may be conserved across different species. *dlo-miR167a* was barely detectable during early longan SE stages; during late developmental stages, however, it was strongly expressed, suggesting a major role in SE during cotyledonary and mature embryonic stages. This is consistent with previous studies [16]. In addition, *dlo-miR390a.1* and dlo-miR390a*.1, differing by only 2 mismatches, showed low expression during early and late SE stages but high expression during middle stages; *dlo-miR390a.1* was not detected in longan globular and mature embryos, but was expressed in orange globular embryos [16]. *dlo-miR160a* and *dlo-miR26* were highly expressed in the middle stages of SE, implying that their accumulation may be required for heart- and torpedo-shaped embryo formation and morphogenesis. On the other hand, *dlo-miR160a* was barely detectable during the period from the friable-embryogenic callus stage (stage 1) to the incomplete compact pro-embryogenic culture stage (stage 3), and *dlo-miR26* was not detected in mature embryos; this suggests that their targets of regulation were consistently promoted.

Members of the third group, comprising *dlo-miR156*, dlo-miR166c*, dlo-miR 168a*, *dlo-miR397a*, dlo-miR 2089*-1, *dlo-miR24*, *dlo-miR17*, and *5S rRNA*, were expressed at varied levels in different embryogenic tissues, suggesting their wide involvement in various developmental stages during longan SE. *dlo-miR156a* and *dlo-miR156c* were both highly expressed during early stages of longan SE. *dlo-miR156a* was strongly expressed in cotyledonary embryos, whereas *dlo-miR156c* expression was concentrated in embryogenic calli II (stage 2). dlo-miR166c* showed high expression during friable-embryogenic callus through compact pro-embryogenic culture stages, moderate expression in globular embryos, and weak expression in cotyledonary and mature embryos; this indicates it may play a key role in early stages of longan SE. In contrast, *miR166* has been found to be abundantly expressed in orange cotyledonary embryos [16] and *Pinus taeda* zygotic embryos [18]. dlo-miR 168a* expressed relatively high levels from heart-shaped embryos to mature embryos, expecially in cotyledonary embryos, and high expression in cotyledonary embryos and moderate expression elsewhere was observed for both *dlo-miR397a* and *dlo-miR24*. In addition to *dlo-miR17*, which was weakly expressed during late developmental stages, the remaining miRNAs dlo-miR2089*-1 and *5S rRNA* showed ubiquitous expression during longan SE.

In summary, the above results indicate that different miRNA families and their individual members, as well as miRNAs*, have different regulatory roles during longan SE.

## Discussion

In this study, 29 novel and 643 conserved miRNAs in more than 169 miRNA families were identified during longan SE by Solexa sequencing. This research represents the first large-scale cloning and characterization of plant SE-related miRNAs. Although many miRNAs remain to be identified, our results reveal that longan has a larger and more diverse miRNA (snRNA) population than other plants [11], [15], [16] when conserved miRNAs are compared. Bioinformatics prediction, degradome analysis, and modified RLM-RACE demonstrated that putative miRNA targets were involved in a broad variety of biological processes, including plant metabolism, signal transduction, and stimulus response. The stage- and tissue-specific expression patterns observed for 24 conserved and novel miRNAs suggest their probable roles in longan SE, and imply that different miRNA families are responsible for morphogenesis at different developmental stages.

### Putative Functions of miRNAs during Longan SE Based on Analysis of Expression Patterns

SE is a multi-step regeneration process which begins with proembryogenic mass formation followed by somatic embryo formation, maturation, desiccation, and plant regeneration. This complicated process involves a large number of genes with complex expression patterns. By identifying miRNAs and their targets and studying their expression patterns, we can propose possible roles for miRNAs during longan SE.

The largest miRNA family in plants–*miR156*–plays critical regulatory roles during juvenile–adult transitions [40] and flowering [41] by targeting *SPL* genes. In *Arabidopsis*, beginning as early as the eight-cell stage, *miR156*-mediated repression of zygotic *SPL10* and *SPL11* prevents premature accumulation of transcripts from genes normally induced during the embryonic maturation phase [14]. In the study, the highest concentrations of *dlo-miR156a* were observed in cotyledonary embryos, which is consistent with *miR156* patterns in larch [15]. In contrast, *dlo-miR156c* expression was strongest in embryogenic calli II (stage 2), similar to results in citrus, in which *miR156* levels were at a maximum during the E2 (EC induced for 2 weeks) stage [16]. These results suggest that *miR156* members play regulatory roles during early stages of longan SE or cotyledonary embryo development by targeting *SPL,* and further imply that functional diversification can take place through the existence of multicopy miRNA families.

In *Arabidopsis*, ABA induction of *miR159* controls transcript levels of 2 *MYB* factors during seed germination [42]. In our study, *dlo-miR159* family transcripts accumulated during middle and late stages of longan SE. This result is similar to findings with respect to *miR159* at the cotyledonary embryo stage of larch [15]. It is not consistent, however, with the pattern observed in citrus, in which *miR159* was expressed at significantly higher levels in globular-shaped embryos [16]. These contrasting results suggest that the role of *miR159* differs between species, and imply that *miR159* may function in middle and late stages of longan SE by targeting Unigene 66155 (*MYB33*).

dlo-miR159b*, *dlo-miR160a*, *dlo-miR167a*, *dlo-miR390*, *dlo-miR398b*, and *dlo-miR26* may have stage-specific functions, because they exhibit tissue-specific expression during longan SE. dlo-miR159b*, the complementary strand of mature functional miRNA, exhibited an expression pattern similar to that of other *miR159* family members; unlike them, it was not expressed in mature cotyledonary embryos, demonstrating that it regulates different targets during longan SE. *miR160*, which regulates the expression of *ARF16* and *ARF17* during embryonic development in *Arabidopsis* [43], was barely detectable during early stages of longan SE. It was instead most highly expressed during heart- and torpedo-shaped embryonic stages, suggesting that *dlo-miR160a* is involved in longan heart- and torpedo-shaped embryo formation and morphogenesis. This also contrasts with larch *miR160*, which may play a regulatory role during cotyledonary embryo development. *miR167* regulates plant fertility by targeting *ARF6* and *ARF8* in response to auxin signal [44]–[46]. In our study, the strongly-expressed *miR167a* appears to play a major role during cotyledonary and mature embryonic stages by regulating *ARF*s (Unigenes 65933/62178, *ARF3*/*8*), which is consistent with previous studies in larch and oranges [15], [16]. In rice, *miR167* levels gradually decrease when cells cultured in the presence of auxin are transferred to an auxin-free medium [47]. Surprisingly in our study, *dlo-miR167a* was undetectable in a medium containing 2,4-D, perhaps because of the endogenous hormone levels present in the longan embryogenic cultures. *miR167* can be regulated by ABA and GA_3_ [48]. A previous study found that during longan SE, the ratio of endogenous ABA/GA_3_ gradually increases [49]; in our study, the expression trend of *dlo-miR167a* generally mirrored that of ABA/GA_3_, indicating that *dlo-miR167a* accumulates in response to endogenous auxin, and that reduced expression of its targets is required for cotyledonary and mature embryo morphogenesis in longan. Like *miR160*, *dlo-miR390 a.1* and -a*.1 also accumulated during heart- and torpedo- shaped embryonic stages. Previous studies have shown that *miR390* directs the formation of ta-siRNAs, which target *ARF2*, *ARF3*, and *ARF4* genes to modulate developmental timing and patterning in *Arabidopsis* [50], and that this miRNA is most highly expressed in citrus in globular-shaped embryos [16] and in larch in cotyledonary embryos [15]. These differing results among different species may be a consequence of different *miR390* members being examined, and suggest that a strict division of labor may exist in this miRNA family.

During plant embryonic development, cells not only increase in number, but also undergo differentiation. By inducing autonomous cell division, oxidative stress can modulate plant SE [51], [52]. *miR398* is the first miRNA to be directly linked to regulation of plant oxidative stress response via down-regulation of *CSD* expression [37]. Recent studies have shown that *miR398* is required for cotyledon-shaped embryo morphogenesis in citrus [16] and is involved in modulation of proembryogenic mass propagation and transition to single embryos in larch [15]. Surprisingly, *dlo-miR398a* was barely detected during longan SE in our study (data not shown). Moreover, in a previous study, *pre-miR398a* was only weakly expressed during early longan SE stages and was not detected at all during middle and late stages [53]. These results indicate that *dlo-miR398a* is not a key developmental factor in longan SE. In addition, the low expression of *dlo-miR398a* led to increased accumulation of its target (*DlCSD1a*), such that mRNA cleavage barely took place; this may explain why cleaved *DlCSD1a* mRNAs were not detected using modified RLM-RACE in our study. In contrast to *dlo-miR398a*, the *dlo-miR398b* family was highly expressed in heart-shaped, torpedo-shaped, and cotyledonary embryos, suggesting its involvement in these developmental stages. *miR398* targets *CSD* and is down-regulated under stress conditions [39]. In our study, the addition of 5% sucrose to the mature embryo induction medium produced a stress-like osmotic pressure. Low expression levels of *dlo-miR398b* during cotyledonary embryo maturation permitted accumulation of *DlCSD*2a as a response to this stress, promoting maturation of longan cotyledonary embryos, consistent with a previous report [16].

High expression in cotyledonary embryos and moderate expression elsewhere was observed for both *dlo-miR397a* and *dlo-miR24*. *miR397* is known to target laccases, which are associated with cell wall lignification and thickening during secondary cell growth [54]. In citrus, *miR397* is specifically expressed at high levels in globular embryos; in larch, *miR397* levels are highest in early cotyledonary embryos–consistent with our results–with low levels observed for its target mRNA. We consequently propose that *dlo-miR397a* helps regulate cell wall thickness during SE through cleavage of laccase mRNA.

*miR166* targets HD-ZIP III transcription factors [55] and is required for cotyledon-shaped embryo morphogenesis [15], [16]. In our study, similar targets, such as Unigenes 12408 and 14044 (class III HD-Zip proteins 4 and 5), were also predicted for *dlo-miR166*, demonstrating the evolutionarily conserved nature of these miRNA targets in different plants. In another study, *miR166* was induced by gibberellic acid (GA_3_) [56]. Expression levels of endogenous GA_3_ showed a gradual downward trend during longan SE [49], which was mirrored in our study by dlo-miR166c* expression levels. This suggests that changes in miR166c* levels are caused by alterations in endogenous GA_3_ concentrations. GA_3_ inhibits the early stage of embryogenic cell differentiation/development leading to globular embryos [57]; dlo-miR166c* in our study exhibited a decreasing level of expression during this transition, suggesting a role for dlo-miR166* during early stages of longan SE. dlo-miR168a* expressed relatively high levels from stages heart-shaped embryos to mature embryos, expecially in cotyledonary embryos, which is consistent with *miR168* from larch [15]. *miR168* is associated with repression of *AGO1* accumulation, and AGO1 is the most important AGO protein in the miRNA pathway, which has specific expression during carrot SE [19]. Therefore, we speculated the accumulation of dlo-miR168a* during cotyledonary embryos develop- ment repressed the expression of *AGO1*, thus leading to the development of longan embryo.

In addition, several miRNAs, such as *dlo-miR80*8, −2089* and −*5077* were also detected in longan SE, which have rarely been studied in plant development. Among them, *dlo-miR80*8 and −*5077* both showed highly expression embryos at late developmental stages of longan somatic embryo. the former reaches an abundance peak at cotyledonary embryos; and the later, which is similar to *osa-miR5077*, reaches peak at mature embryos, suggesting the accumulation of the two miRNAs is required for the development of somatic embryo at late stages. Besides, miR2089*, which considered to be legume-specific [58], showed ubiquitous expression levels and exerted regulatory function during longan SE. Further, the expression analysis of the 4 novel miRNAs uncovered in this study (*dlo-miR4a, dlo-miR17*, *dlo-miR26*, and *dlo-miR24*), showed varied levels in different embryogenic tissues during longan SE, has laid a foundation for future functional studies of longan embryogenesis. Such a study is currently underway in our laboratory. The investigation reported here represents a small but significant step towards the elucidation of functions of miRNAs during longan embryogenesis.

## Materials and Methods

### Plant Materials and RNA Isolation

Synchronized embryogenic cultures at different developmental stages were obtained following previously published methods for longan [1], [2], [58]–[60]. The synchronized cultures, consisting of friable-embryogenic calli, embryogenic calli II, incomplete compact pro-embryogenic cultures, compact pro-embryogenic cultures, globular embryos, heart-shaped embryos, torpedo-shaped embryos, cotyledonary embryos, and mature embryos, were collected and stored at −80°C for later use. Total RNAs were extracted from the above-described cultures using Trizol reagent (Invitrogen, Carlsbad, CA, USA). Only RNA samples with *A260/A280* ratios between 1.9 and 2.1 and *A260/A230* ratios higher than 2.0 were used for further analyses.

### Longan sRNA cDNA Library Construction, Solexa Sequencing, and Data Analysis

To identify potential conserved and novel miRNAs from longan SE, a pooled sRNA library was generated from the synchronized embryogenic cultures (friable-embryogenic calli, globular embryos, heart-shaped embryos, torpedo-shaped embryos, and cotyledonary embryos) and sequenced on a Solexa system (Illumina). First, small (16–30 nt) RNA fragments were isolated from a 15% PAGE gel and purified. The small RNAs were then sequentially ligated to a 5′ RNA adapter (5′-GUUCAGAGUUCUACAGUCCGACGAUC-3′) and a 3′ RNA adapter (5′-pUCG UAUGCCGUCUUCUGCUUGidT-3′; p, phosphate; idT, inverted deoxythymidine) using T4 RNA ligase. The resulting samples were reverse- transcribed to cDNA with an RT primer (5′-CAAGCAGAAGACGGCATACGA-3′) using Superscript II reverse transcriptase (Invitrogen) and PCR-amplified. Finally, Solexa sequencing was used to sequence the sRNAs in the pooled longan samples (Beijing Genomics Institute, China).

Bioinformatics analyses were conducted on the resulting Solexa sequencing data. The 35-nt sequence tags were first trimmed of adaptors, regions of low complexity, and low-quality sequences, and the length distribution of clean tags was summarized. The remaining sRNA sequences (clean reads) were mapped to both a longan embryogenic callus transcriptome (SRA050205) and a *P. trichocarpa* genome. To classify degradation fragments of noncoding RNA, the clean tags were then compared against non-coding RNAs in Rfam and NCBI GenBank databases. Any sRNAs having exact matches to these sequences were excluded from further analysis. To identify conserved miRNAs in longan, the unique sRNA sequences were searched using miRBase 17.0. Novel miRNAs were predicted from unannotated sRNAs using Mireap, and their secondary structures were predicted using the computational software package Mfold 3.1. At the same time, first position base bias among sRNA candidates of certain lengths was summarized to determine prediction accuracy. Finally, to understand the biological functions of miRNAs, potential longan novel and conserved miRNA targets were predicted as described in [41], [61].

### Degradome cDNA Library Construction and Sequencing

To experimentally verify both conserved and novel miRNA targets in longan, a degradome cDNA library from the above pooled total RNA samples was constructed using the following steps: ligation of polyA-enriched RNA samples to a custom RNA adaptor containing an *Mme*I site, followed by reverse transcription (RT), second- strand synthesis, *Mme*I digestion, ligation of a 3′ double-stranded DNA adaptor, gel purification, and PCR amplification. The amplified degradome tag library was sequenced on a Solexa/Illumina genome analyzer. After sequencing, 20- and 21-nt reads from the degradome library were analyzed using PairFinder to identify sRNA targets.

### psRNATarget and Modified 5′ RLM-RACE

To verify the nature of predicted *miR398* targets and to investigate possible regulation of *CSD* by *miR398* during longan SE, a psRNATarget [62] analysis and a modified 5′ RLM-RACE experiment were performed. For the psRNATarget analysis, sequences of the newly-identified longan *miR398* were matched against nucleic acid sequences of longan *SOD* gene families (*Cu/Zn-SOD*, *Fe-SOD*, and *Mn-SOD*). All predicted targets were scored using criteria described in [62]. Any sequences with total scores less than 5.0 were considered to be miRNA targets. To identify cleavage sites of target transcripts, a modified RLM-RACE experiment was performed using a GeneRacer kit (Invitrogen). Briefly, total RNAs from equal mixtures of friable- embryogenic calli, globular embryos, heart-shaped embryos, and torpedo- shaped embryos were ligated to a 5′ RACE RNA adapter. The resulting samples were reverse-transcribed to cDNA using a GeneRacer Oligo dT primer and Superscript III reverse transcriptase, and PCR-amplified using GeneRacer 5′ primers and gene- specific primers (Table S5 in Supporting Information S1). The resulting PCR products were gel-purified, cloned, and sequenced.

### Real-time Quantitative PCR of Longan Conserved and Novel miRNAs

qPCR was used to validate results obtained from high-throughput sequencing of longan miRNAs. RNA samples from the 9 embryogenic cultures described above were reverse-transcribed using an NCode VILO miRNA cDNA Synthesis kit. Expression profiles of 24 miRNAs were examined using an NCode Express SYBR GreenER miRNA qPCR kit (Invitrogen). All reactions were performed in triplicate in a LightCycler 480 qPCR instrument (Roche Applied Science, Switzerland), with a dissociation curve used to control for primer dimers in the reactions. Mature miRNA abundance was calculated relative to expression of reference genes *dlo-miR156c*, dlo-miR2089*-1, and *5S rRNA*. miRNA names and primer sequences are provided in Table S6 in Supporting Information S1.

## Supporting Information

Supporting Information S1 File containing the following supporting information tables Table S1. Coserved and non-coserved longan miRNAs. Table S2. Longan-known miRNA targets by predicition method. Table S3. Identifiction targets for longan miRNAs by Degradome sequencing. Table S4. The consensus targets for longan miRNAs obtaining from prediction and degradome analysis. Table S5. Primer sequences. Table S6. The miRNA names, primer sequences.
(ZIP)
