# Supplementary material for: Comparative Analysis Reveals Dynamic Changes in miRNAs and Their Targets and Expression during Somatic Embryogenesis in Longan (Dimocarpus longan Lour.)
Source: PLoS One. 2013 Apr 11;8(4):e60337. doi: 10.1371/journal.pone.0060337 (PMC3623967; doi:10.1371/journal.pone.0060337)
Supplement: Supporting Information S1 File containing the following supporting information tables — Table S1. Coserved and non-coserved longan miRNAs. Table S2. Longan-known miRNA targets by predicition method. Table S3. Identifiction targets for longan miRNAs by Degradome sequencing. Table S4. The consensus targets for longan miRNAs obtaining from prediction and degradome analysis. Table S5. Primer sequences. Table S6. The miRNA names, primer sequences. (ZIP) [file pone.0060337.s001.zip › Supporting Information files/Table S1 coserved and non-coserved longan miRNAs.doc]

**Table S1** coserved and non-coserved longan miRNAs

| **conserved longan miRNA** | | | | | |
| --- | --- | --- | --- | --- | --- |
| family | gene name | count(total) | length | small RNA sequence | hairpin |
| MIR156 | dlo-miR156a | 730516 | 20 | TGACAGAAGAGAGTGAGCAC | longan-m0005 |
|  | dlo-miR156b | 3616 | 21 | CTGACAGAAGATAGAGAGCAC | no_hairpin |
|  | dlo-miR156c | 15261 | 22 | TTTGACAGAAGAGAGTGAGCAC | longan-m0005 |
|  | dlo-miR156d | 15 | 21 | TTGACAGAAGAGAGGGAGCAC | no_hairpin |
|  | dlo-miR156e | 146 | 20 | TGACAGAGGAGAGTGAGCAC | no_hairpin |
|  | dlo-miR156f | 18 | 22 | TTGACAGAAGAGAGTGAGCACA | longan-m0005 |
|  | dlo-miR156g | 452 | 20 | CGACAGAAGAGAGTGAGCAC | no_hairpin |
|  | dlo-miR156h | 2 | 20 | TGATAGAAGAAAGTGAGCAC | no_hairpin |
|  | dlo-miR156k | 524 | 21 | TGACAGAAGAGAGAGAGCACA | no_hairpin |
|  | dlo-miR156l | 35 | 21 | CGACAGAAGAGAGTGAGCACA | no_hairpin |
|  | dlo-miR156a* | 5 | 21 | TGCTCACTTCTCCTCTGTCAG | longan-m0014 |
|  | dlo-miR156d*.1 | 22 | 21 | GCTCACTTCTCTTTCTGTCAGC | longan-m0005 |
|  | dlo-miR156d*.2 | 42 | 22 | TGCTCACTTCTCCTCTGTCAGC | longan-m0014 |
|  | dlo-miR156e* | 263 | 21 | GCTTACTCTCTATCTGTCACC | no_hairpin |
|  | dlo-miR157a | 81 | 21 | TTGACAGAAGATAGAGAGCAC | no_hairpin |
|  | dlo-miR157d | 591 | 20 | TGACAGAAGATAGAGAGCAC | no_hairpin |
|  | dlo-miR529 | 881 | 24 | AGAACAGAGAGAGACACAACTCAC | no_hairpin |
|  | dlo-miR1088-5p | 3 | 22 | CAGAAGAAATGTAGAGGAGCAT | no_hairpin |
| MIR158 | dlo-miR158b | 71 | 20 | TCAACAAATGTAGCAAAGTC | no_hairpin |
| MIR159 | dlo-miR159a.1 | 3086 | 21 | TTTGGATTGAAGGGAGCTCTA | no_hairpin |
|  | dlo-miR159a.2 | 343 | 20 | TTTGGATTGAAGGGAGCTTA | no_hairpin |
|  | dlo-miR159b | 63 | 21 | TTTGGATTGAAGGGAGCTCTT | no_hairpin |
|  | dlo-miR159c | 36 | 21 | TTTGGATTGAAGGGAGTCCTA | no_hairpin |
|  | dlo-miR159f | 1 | 21 | CTTGGATTGAAGGGAGCTCTA | no_hairpin |
|  | dlo-miR159b* | 16 | 21 | GAGCTCCTTGAAGTCCAATAG | no_hairpin |
|  | dlo-miR319 | 99 | 22 | TAGCTGCCGACTCATTCATCCA | longan-m0013 |
|  | dlo-miR319a | 210 | 21 | TTGGACTGAAGGGAGCTCCCT | no_hairpin |
|  | dlo-miR319c | 13 | 19 | TTGACTGAAGAGGCTCCTA | no_hairpin |
|  | dlo-miR319b* | 3 | 21 | AGAGCTTTCTTCAGTCCACTT | no_hairpin |
| MIR160 | dlo-miR160a | 19 | 21 | TGCCTGGCTCCCTGTATGCCA | no_hairpin |
|  | dlo-miR160a* | 208 | 21 | GCGTATGAGGAGCCATGCATA | no_hairpin |
|  | dlo-miR160d* | 8 | 22 | AGCGTGCGAGGAGCCATGCATG | no_hairpin |
| MIR162_1 | dlo-miR162a | 891 | 21 | TCGATAAACCTCTGCATCCAG | no_hairpin |
|  | dlo-miR162a* | 9 | 21 | GGAGGCAGCGGTTCATCGATC | no_hairpin |
| MIR164 | dlo-miR164a | 44 | 21 | TGGAGAAGCAGGGCACGTGCA | no_hairpin |
| MIR166 | dlo-miR165a | 87 | 21 | TCGGACCAGGCTTCATCCCCC | no_hairpin |
|  | dlo-miR166a | 22387 | 21 | TCGGACCAGGCTTCATTCCCC | longan-m0011 |
|  | dlo-miR166b | 10 | 20 | TCGGACCAGGCTTCATTCCA | no_hairpin |
|  | dlo-miR166e | 1987 | 21 | TTCGGACCAGGCTTCATTCCC | no_hairpin |
|  | dlo-miR166f | 817 | 21 | TCTCGGACTAGGCTTCATTCC | no_hairpin |
|  | dlo-miR166g | 59 | 21 | TCGGACCAGGCTTCATTCCTC | no_hairpin |
|  | dlo-miR166h | 691 | 20 | TCGGACCAGGCTTCATTCCC | longan-m0011 |
|  | dlo-miR166j | 18 | 21 | TCGGATCAGGCTTCATTCCCC | no_hairpin |
|  | dlo-miR166k | 8 | 19 | TCGGACCAGGCTTCATTCT | no_hairpin |
|  | dlo-miR166m | 30 | 21 | TCGGACCAGGCTTCATTCCCT | no_hairpin |
|  | dlo-miR166n | 16 | 19 | TCGGACCAGGCTTCATTTT | no_hairpin |
|  | dlo-miR166c* | 346 | 21 | GGAATGTTGTCTGGCTCGAGG | longan-m0011 |
| MIR167_1 | dlo-miR167a | 5518 | 21 | TGAAGCTGCCAGCATGATCTA | no_hairpin |
|  | dlo-miR167c | 8 | 21 | TGAAGCTGCCAGCATGATCTC | no_hairpin |
|  | dlo-miR167d | 63 | 22 | TGAAGCTGCCAGCATGATCTAG | no_hairpin |
|  | dlo-miR167f | 164 | 21 | TGAAGCTGCCAGCATGATCTT | no_hairpin |
|  | dlo-miR167b* | 7 | 23 | GGTCATGCTCTGACAGCCTCACT | no_hairpin |
| MIR168 | dlo-miR168a | 9059 | 21 | TCGCTTGGTGCAGGTCGGGAA | no_hairpin |
|  | dlo-miR168a | 12 | 24 | GATCCCGCCTTGCATCAACTGAAT | no_hairpin |
|  | dlo-miR168a* | 404 | 21 | CCCGCCTTGCATCAACTGAAT | no_hairpin |
| MIR169_2 | dlo-miR169a | 25 | 21 | CAGCCAAGGATGACTTGCCGA | no_hairpin |
| MIR171 | dlo-miR171 | 92 | 21 | TTGAGCCGCGTCAATATCTCC | no_hairpin |
|  | dlo-miR171b | 392 | 21 | TGATTGAGCCGTGCCAATATC | no_hairpin |
|  | dlo-miR171c | 103 | 21 | TATTGGTTCGGTTCAATGAGA | no_hairpin |
|  | dlo-miR171d | 24 | 20 | TTGAGCCGCGCCAATATCAC | no_hairpin |
|  | dlo-miR171f | 167 | 21 | TTGAGCCGCGCCAATATCACT | no_hairpin |
|  | dlo-miR171c* | 43 | 21 | CGATATTGGTGGGGTTCAATC | no_hairpin |
|  | dlo-miR171c* | 26 | 21 | ATTGGTGCGGTTCAATCTTAA | no_hairpin |
|  | dlo-miR479 | 20 | 24 | TGTGGTAATTGGTAGTCGACTCAT | no_hairpin |
| MIR172 | dlo-miR172a | 24 | 21 | AGAATCTTGATGATGCTGCAT | no_hairpin |
| MIR390 | dlo-miR390a.1 | 17 | 20 | CGCTATCCATCCTGAGTTTC | no_hairpin |
|  | dlo-miR390a.2 | 37 | 20 | CGCTATCTATCCTGAGTTTC | longan-m0015 |
|  | dlo-miR390a.3 | 2365 | 21 | AAGCTCAGGAGGGATAGCGCC | longan-m0015 |
|  | dlo-miR390b | 8 | 21 | CAGCTCAGGAGGGATAGACAA | no_hairpin |
|  | dlo-miR390a.1* | 148 | 21 | CGCTATCTATCCTGAGTTTCA | longan-m0015 |
|  | dlo-miR390a.2* | 723 | 21 | CGCTATCCATCCTGAGTTTCC | no_hairpin |
| MIR393 | dlo-miR393 | 7 | 21 | TTCCAAAGGGATCGCATTGAT | no_hairpin |
| MIR394 | dlo-miR394a | 8 | 20 | TTGGCATTCTGTCCACCTCC | longan-m0004 |
| MIR395 | dlo-miR395d* | 19 | 21 | TTCCCCAGAACACTTCATTGG | longan-m0008 |
| MIR396 | dlo-miR396a | 799 | 21 | TTCCACAGCTTTCTTGAACTG | longan-m0009 |
|  | dlo-miR396b | 156 | 18 | TTCCACAGCTTTCTTGAA | longan-m0009 |
|  | dlo-miR396e | 12 | 21 | CCACAGCTTTCTTGAACTGCA | longan-m0009 |
|  | dlo-miR396a* | 187 | 21 | GTTCAATAAAGCTGTGGGAAG | longan-m0009 |
|  | dlo-miR396b* | 33 | 20 | GTTCAATAAAGCTGTGGGAA | longan-m0009 |
| MIR397 | dlo-miR397a | 4994 | 21 | TCATTGAGTGCAGCGTTGATG | longan-m0003 |
|  | dlo-miR397 | 25 | 19 | ATTGAGTGCAGCGTTGATG | longan-m0003 |
| MIR398 | dlo-miR398a | 4 | 20 | TGTGTTCTCAGGTCGCCCTT | no_hairpin |
|  | dlo-miR398b.1 | 10 | 20 | TGTGTTCTCAGGTCGCCCCT | longan-m0002 |
|  | dlo-miR398b.2 | 23 | 18 | TGTGTTCTCAGGTCGCCC | longan-m0002 |
|  | dlo-miR398b.3 | 3 | 21 | TGTGTTCTCAGGTCGTCCCTG | no_hairpin |
|  | dlo-miR398b.4 | 456 | 21 | TGTGTTCTCAGGTCGCCCCTG | longan-m0002 |
|  | dlo-miR398a* | 6 | 23 | GGAGTGACTACTGAGAACACAGG | no_hairpin |
| MIR399 | dlo-miR399c | 54 | 21 | TGCCAATGGAGATTTGCCCGG | no_hairpin |
|  | dlo-miR399e | 3 | 18 | CGCCAAAGGAGAATTGCC | no_hairpin |
|  | dlo-miR399f | 24 | 21 | TGCCAAAGGAGATTTGCCCGG | no_hairpin |
|  | dlo-miR399i | 7 | 21 | CGCCAAAGGAGAGTTGCCCTG | no_hairpin |
|  | dlo-miR399l | 4 | 18 | CGCCAAAGGAGAGTTGCC | no_hairpin |
| MIR400 | dlo-miR400 | 26 | 18 | ATGATGTATTATAAGCAC | no_hairpin |
| undef | dlo-miR401 | 384 | 21 | CGAACACTGCTGTCGACGCCA | no_hairpin |
| MIR403 | dlo-miR403 | 822 | 19 | TTAGATTCACGCACAAACT | no_hairpin |
| undef | dlo-miR406 | 5 | 21 | TAGCTGCTGTTGATAATCCAG | no_hairpin |
| undef | dlo-miR407 | 13 | 23 | TTTAAATTATAGACTCTTTGGTT | no_hairpin |
| MIR408 | dlo-miR408 | 5550 | 21 | ATGCACTGCCTCTTCCCTGGC | longan-m0007 |
|  | dlo-miR408 | 2976 | 21 | ACGGGGAACAGGCAGAGCATG | longan-m0007 |
|  | dlo-miR408* | 3727 | 21 | CGGGGAACAGGCAGAGCATGG | longan-m0007 |
| undef | dlo-miR413 | 3 | 24 | AAGCTTTCTCTTTGATTCTGCACA | no_hairpin |
| MIR414 | dlo-miR414 | 36 | 24 | ATACATCTTCAATCATATCGTCAT | no_hairpin |
| undef | dlo-miR415 | 52 | 24 | AAGCAGAACGCAGAACAGAAGCAG | no_hairpin |
| undef | dlo-miR416 | 307 | 21 | GATGTCTACGTCCACTGTTCA | no_hairpin |
| undef | dlo-miR417 | 11 | 24 | AGAAGTAGTGGAATTTGTGTCGAG | no_hairpin |
| undef | dlo-miR419 | 184 | 21 | TGATAATGCTGAGATATGTTG | no_hairpin |
| undef | dlo-miR420 | 452 | 19 | GAAACAATCACGAAATGCG | no_hairpin |
| MIR437 | dlo-miR437c | 173 | 24 | AAGAGCTTAGAGAAGGTCTGGCTT | no_hairpin |
| MIR439 | dlo-miR439a | 9 | 20 | CGTCGAACCGTCGGTTTTCA | no_hairpin |
| undef | dlo-miR440 | 25 | 24 | AGTGTTCAACTGATGACGGGACAA | no_hairpin |
| MIR441 | dlo-miR446 | 14 | 24 | TCATCAATCGAAATGGAGAAATGG | no_hairpin |
|  | dlo-miR808.1 | 1 | 24 | ATGAGATCAGTGGGAAAAGAAGAA | no_hairpin |
|  | dlo-miR808.2 | 9 | 24 | ATGAATGGTGGGAGAAGTAAGATA | no_hairpin |
|  | dlo-miR809 | 8 | 24 | TGAAGATGAGAAGAATGTTAGAAT | no_hairpin |
|  | dlo-miR1126 | 5 | 24 | TCCATATGGACTGCAAAGGCGGAG | no_hairpin |
|  | dlo-miR1132 | 91 | 19 | CTATTATGGAAGGAAGGCA | no_hairpin |
| MIR444 | dlo-miR444b | 41 | 21 | TACAATTCTGTCGTCAAGCTT | no_hairpin |
| MIR445 | dlo-miR445a | 22 | 23 | TAAATTAGTTTTAAGACATCGGA | no_hairpin |
|  | dlo-miR2124a | 1 | 24 | ACTTTAAACTGTGTTGTCCTGTAT | no_hairpin |
| MIR478 | dlo-miR478d | 1 | 22 | TGACGTTCATCTATTTAGGAAT | no_hairpin |
| MIR480 | dlo-miR480a | 19 | 23 | ACTACAAATAATGACGTTGAACA | no_hairpin |
| MIR481 | dlo-miR481d | 6 | 24 | GGAAGACCTCACCTAACAGTGAAC | no_hairpin |
| MIR528 | dlo-miR528 | 25 | 20 | CTGGAAGTGGATGCAGAGGG | no_hairpin |
| MIR534 | dlo-miR534a | 6 | 24 | ATGTCCATTGTCCAGCATGCATAC | no_hairpin |
| undef | dlo-miR536 | 11 | 20 | TCGTGCAAGCTGTGGTGCAT | no_hairpin |
| MIR773 | dlo-miR773b* | 21 | 24 | AAATTTGTTATCGTCAAGGATATT | no_hairpin |
| MIR774 | dlo-miR774b | 174 | 24 | TAGAGATGGAGATTTGGATGGACA | no_hairpin |
| undef | dlo-miR778 | 119 | 19 | TGGCTTCGTTTTTGTACAC | no_hairpin |
| undef | dlo-miR780 | 353 | 18 | TCTGAAGCTGTTGAAGGT | no_hairpin |
| MIR806 | dlo-miR806a | 3 | 24 | ATGTTGCTAACAAAGTCAAACGGT | no_hairpin |
|  | dlo-miR2123a | 24 | 24 | TAAAAGGTCAACGGTTCAAATCAC | no_hairpin |
| MIR810 | dlo-miR810b | 10 | 23 | AAAGTGATTTTAATTATGACGGT | no_hairpin |
| MIR811 | dlo-miR811d | 13 | 24 | TAATCTTGGATGATGCATGGACGG | no_hairpin |
|  | dlo-miR2862 | 4 | 24 | TCCTCAACAGCTCTAGATCGTCCA | no_hairpin |
| MIR812 | dlo-miR812n | 34 | 24 | AGGATAAATCAGGCTGCACTTCAA | no_hairpin |
|  | dlo-miR1884b | 3 | 24 | ATGATGACGGTGTTGACTGTTATA | no_hairpin |
| MIR815 | dlo-miR815a | 39 | 19 | AAGCGGACTGGGAGATTGG | no_hairpin |
| undef | dlo-miR816 | 10 | 20 | AGGACATATTTTTACACAAC | no_hairpin |
| MIR818 | dlo-miR1436 | 36 | 19 | AGTTAGTGACGGAGGGAGT | no_hairpin |
|  | dlo-miR1442 | 79 | 18 | ATTCATAGTCTTCATGTG | no_hairpin |
| MIR820 | dlo-miR820a | 4 | 22 | TCGGCCTGTGGATGGCAAGCAG | no_hairpin |
| MIR821 | dlo-miR821d | 536 | 20 | AAGCTCAAGAACGAAAGTTG | no_hairpin |
| MIR823 | dlo-miR823 | 30 | 24 | TGGTGTAGGTGTCATATAAGCATT | no_hairpin |
| MIR825 | dlo-miR825 | 2 | 24 | TTCCAATGAAGTGTTGCATGAACC | no_hairpin |
| MIR829 | dlo-miR829 | 119 | 24 | AGCTCTGATACCAATTGATGTGAA | no_hairpin |
| undef | dlo-miR830 | 49 | 19 | AAGTATGTTGAGAAAAGTG | no_hairpin |
| MIR831 | dlo-miR831* | 34 | 24 | AGAATGGTAGAAGGAGATAGAGCA | no_hairpin |
| undef | dlo-miR832 | 97 | 24 | TGACTGCAGGAATCGGAATCGAAA | no_hairpin |
| MIR833 | dlo-miR833 | 3 | 24 | TAGTTATCTGAACCTCTGCCTAGT | no_hairpin |
| MIR835 | dlo-miR835 | 56 | 20 | TAGATGAAGATCGAAGAAAG | no_hairpin |
| undef | dlo-miR836 | 812 | 22 | TTGTGTTTCAATTTGATCGTGG | no_hairpin |
| MIR837 | dlo-miR837 | 56 | 24 | AAACTGAACAAACAAAGCTGACGG | no_hairpin |
| MIR838 | dlo-miR838* | 40 | 24 | GTGCAAGAATGTAGAGACGCAAAG | no_hairpin |
| MIR839 | dlo-miR839* | 21 | 24 | GATCGCATGAAGGTTTGGCTGGAA | no_hairpin |
| MIR840 | dlo-miR840 | 22 | 24 | ACACTGACAGGACCCAAACCAGAC | no_hairpin |
| MIR841 | dlo-miR841 | 19 | 23 | TAGAGCCACTTGAAGACTGACTA | no_hairpin |
| MIR842 | dlo-miR842* | 27 | 19 | AGATTGATCGTCCATGATG | no_hairpin |
| undef | dlo-miR843 | 13 | 20 | TCATAGTCGAGCTCATTGGA | no_hairpin |
| MIR844 | dlo-miR844 | 34 | 24 | AGTTTAAGGACTAGTAATGATGGT | no_hairpin |
| MIR845_1 | dlo-miR845a | 38 | 24 | CGAAGCTCTGATACCAATTGATGT | no_hairpin |
|  | dlo-miR845a | 16 | 24 | AAGCTCTGATACCAATTGATGTGA | no_hairpin |
|  | dlo-miR845b | 31 | 24 | ACTGCTCTGATACCAAAATGATGC | no_hairpin |
|  | dlo-miR845d | 57 | 24 | TTGGCTCTGATACCAAGTTGACGT | no_hairpin |
| MIR846 | dlo-miR846 | 1 | 24 | ATGAATGTGAAGTGGTCTTGAATT | no_hairpin |
| MIR847 | dlo-miR847* | 93 | 24 | TTTGAATGGACAGAGGAATGCGAA | no_hairpin |
| undef | dlo-miR850 | 26 | 24 | TAAATCCCGGACCCTACAACAAAT | no_hairpin |
| MIR851 | dlo-miR851 | 6 | 22 | TGTGGTGGCAAACAAGAGATAA | no_hairpin |
| MIR852 | dlo-miR852* | 8 | 24 | AGAACAAGAGGGCTTCATGTTATT | no_hairpin |
| MIR854 | dlo-miR854a | 358 | 18 | GAGAGAGAGGGAGGAGAG | no_hairpin |
| undef | dlo-miR855 | 7 | 24 | AGAAAAAGTAAGGAAATGAGGCAA | no_hairpin |
| MIR856 | dlo-miR856* | 5 | 24 | ATGATGGTTCATTTGTGGTGATAA | no_hairpin |
| MIR857 | dlo-miR857* | 6 | 24 | AAATCACTTCACCATACAAACATA | no_hairpin |
| undef | dlo-miR857 | 17 | 24 | TATTTGAATTTGAATAGTAGGCTT | no_hairpin |
| MIR859 | dlo-miR859* | 2 | 24 | ATGGATTTTACATAGATTAGATCA | no_hairpin |
| MIR860 | dlo-miR860 | 51 | 19 | TCAATCAGATTGGACATTT | no_hairpin |
| MIR861 | dlo-miR861 | 64 | 19 | ATGGTATATCTTTATGAAC | no_hairpin |
| MIR862 | dlo-miR862 | 28 | 19 | TCCAATAGGTCGGAATCTC | no_hairpin |
| undef | dlo-miR863 | 1 | 24 | ATATGAGAGCAATCAAGAACATAA | no_hairpin |
| undef | dlo-miR865 | 10 | 24 | ATCGAAATTTGTTGATCTAATTGA | no_hairpin |
| undef | dlo-miR866 | 98 | 18 | ACTAGATCGTCTTGAAGA | no_hairpin |
| undef | dlo-miR867 | 34 | 21 | TAGAAACGGATTTATTAGGAA | no_hairpin |
| MIR868 | dlo-miR868* | 20 | 24 | CTGTTCACGTTGCACTTGAGGAGT | no_hairpin |
| MIR869 | dlo-miR869 | 103 | 21 | TTCTGATGATGAGATAGTTGA | no_hairpin |
| undef | dlo-miR870 | 5 | 19 | AATTTGTGCTTCTTCGATT | no_hairpin |
| undef | dlo-miR897 | 20 | 24 | TTGACAAGTGGAAATCAGCAACAG | no_hairpin |
| MIR898 | dlo-miR898a* | 4 | 23 | GCGTGGAGCAGTGCACAGCAATA | no_hairpin |
| MIR899 | dlo-miR2083 | 5 | 24 | ATGGATATGACCGAGTGAAGAAGA | no_hairpin |
| undef | dlo-miR900 | 4 | 24 | TCTCCAGGTCAAGAACATCATGCA | no_hairpin |
| undef | dlo-miR906 | 2 | 24 | GTCCGAAAGATAGCTTCCCCCTGC | no_hairpin |
| undef | dlo-miR908 | 21 | 20 | TACTCGATCAATGAGGCAGC | no_hairpin |
| undef | dlo-miR913* | 160 | 21 | ACGACGTCGTAGGAGTGCAAG | no_hairpin |
| undef | dlo-miR916 | 6 | 18 | CCAAGGTCATCGTTGCCC | no_hairpin |
| MIR918 | dlo-miR918 | 40 | 21 | TGCCTTGTAGCGGACATCTTC | no_hairpin |
|  | dlo-miR919 | 279 | 24 | AATTCGAGATGACTGACATCGGAT | no_hairpin |
| MIR946 | dlo-miR946a* | 1114 | 23 | TGTGGATAGCAGAAGGCTTTGTC | no_hairpin |
| undef | dlo-miR948 | 18 | 20 | TCCAGACTGTGTGAGACCGG | no_hairpin |
| MIR951 | dlo-miR951 | 2 | 20 | TGTTCAGTGCGTTGGACCAC | no_hairpin |
| MIR952 | dlo-miR952b | 1084 | 18 | AACGAGGATCCATTGGAG | no_hairpin |
| MIR1023 | dlo-miR1023b | 24 | 24 | AGATTGTGAATTGAGAGAGTGCAT | no_hairpin |
| MIR1024 | dlo-miR1024a | 170 | 19 | TCTGGTTGGATATAGGCTA | no_hairpin |
| MIR1026 | dlo-miR1026a | 156 | 23 | TGAGACACTGACTTGAGAGGAGA | no_hairpin |
| MIR1027 | dlo-miR1027a | 2 | 21 | TTTCTCTTCTTTCTTCCACTC | no_hairpin |
| undef | dlo-miR1028c | 625 | 24 | TGGATCTGTAGGTTTAAACGAGCA | no_hairpin |
| undef | dlo-miR1036 | 13 | 20 | AGCTAATCAAGGATTACGAC | no_hairpin |
| undef | dlo-miR1037 | 7 | 24 | AGAACCTTAGTGTAGATTTGATGG | no_hairpin |
| undef | dlo-miR1038 | 4 | 23 | CATGGTGGAATCAGCGATGCAAG | no_hairpin |
| undef | dlo-miR1039 | 2 | 24 | ATACATTGTGGGTCTTTTCTCCTG | no_hairpin |
| undef | dlo-miR1040 | 73 | 23 | TGAACGTCAAGATGAACATTTCA | no_hairpin |
| undef | dlo-miR1041 | 43 | 19 | TTTTAAGGTAGAGTGGTCT | no_hairpin |
| undef | dlo-miR1043 | 13 | 23 | GTTGCACGTAGAGTTTGAAGGCT | no_hairpin |
| undef | dlo-miR1044 | 17 | 22 | TTTGTAGGTTCTTATTTGTTTT | no_hairpin |
| undef | dlo-miR1046 | 179 | 18 | TGGATTATATTTTTCAAA | no_hairpin |
| undef | dlo-miR1048 | 61 | 19 | TAGAACTGAGTGTAAGACA | no_hairpin |
| undef | dlo-miR1052 | 3 | 24 | ATTCCTTTTCATTAATTGGTGGTA | no_hairpin |
| undef | dlo-miR1056 | 3 | 24 | TCGGATCTTATGATGCATTAACCC | no_hairpin |
| undef | dlo-miR1058 | 30 | 21 | AAGCATTCCATCACAAAGCAA | no_hairpin |
| undef | dlo-miR1061 | 20 | 19 | TAGTTGTCAATGGAAAATG | no_hairpin |
| undef | dlo-miR1064 | 24 | 23 | CGATTGTGAGAATAGTCATCCAG | no_hairpin |
| undef | dlo-miR1067 | 29 | 19 | AAACTGAAGTTGGACGCCA | no_hairpin |
| undef | dlo-miR1069 | 22 | 20 | GAAAAGATCAAAGTGTCACT | no_hairpin |
| undef | dlo-miR1072 | 54 | 23 | TTCAAGTGTGTATTTGAGCTTGA | no_hairpin |
| undef | dlo-miR1074 | 42 | 20 | AGGTTGTTGGTTGGTATGAT | no_hairpin |
| undef | dlo-miR1077 | 3 | 24 | AGGGATTGAGATCGTATACTAGCC | no_hairpin |
| undef | dlo-miR1078 | 8 | 24 | TTTGGATGAATCTCAATTGTCGAG | no_hairpin |
| undef | dlo-miR1081 | 58 | 19 | TGAGGTTGAGTTTGATTTC | no_hairpin |
| undef | dlo-miR1085 | 36 | 22 | TTAGAATATGTCTTGTAGGTGA | no_hairpin |
| undef | dlo-miR1087 | 35 | 24 | ATACAGATTTGGTAGTTATGCTTT | no_hairpin |
| undef | dlo-miR1089 | 6 | 24 | AGAACTCAAGTCTAGGATTGTTGC | no_hairpin |
| undef | dlo-miR1092 | 3 | 23 | TGGACAGGTAATCGCTTGGTGTT | no_hairpin |
| undef | dlo-miR1097 | 35 | 24 | AGTCCATTGTTGATTTGTTGGAGA | no_hairpin |
| undef | dlo-miR1098 | 9 | 22 | TGATGACGTGTGTGATGATAAT | no_hairpin |
| undef | dlo-miR1100 | 9 | 24 | TGGTCACGGACAGCAAACCCCACT | no_hairpin |
| undef | dlo-miR1102 | 10 | 18 | AGCAGCCACCGTCAAGCA | no_hairpin |
| undef | dlo-miR1105 | 107 | 24 | ATTCGGATGATGAAGACTTCGCTG | no_hairpin |
| undef | dlo-miR1108 | 37 | 21 | TGACCTGGAGACCATAACCCT | no_hairpin |
| undef | dlo-miR1111 | 7 | 22 | TCATGACTAAGCAGGACATCAG | no_hairpin |
| undef | dlo-miR1112 | 3 | 21 | TGGATGCTGATAACAAGGTCA | no_hairpin |
| undef | dlo-miR1114 | 35 | 20 | AAAGAGACAAAACATTAGAG | no_hairpin |
| undef | dlo-miR1118 | 1 | 24 | ACTACAATATGGAATGGTGGGAGA | no_hairpin |
| MIR1120 | dlo-miR1120 | 3 | 23 | AAATTTCTTAATTATGGAACGGG | no_hairpin |
|  | dlo-miR1137 | 61 | 18 | TAGTACAAGTGTTTCATC | no_hairpin |
| MIR1122 | dlo-miR1122 | 34 | 19 | TAGTATACATGCATTTGGA | no_hairpin |
|  | dlo-miR1127 | 104 | 19 | AACTACTCCTCGTCTGATG | no_hairpin |
| MIR1139 | dlo-miR1139 | 25 | 20 | AAGTAACTACACTACTAACT | no_hairpin |
| undef | dlo-miR1145 | 2 | 23 | TGGCGTTGACCCTGACCGCTGGC | no_hairpin |
| undef | dlo-miR1146 | 6 | 20 | ATGGGTCGGATGGGAAGGTA | no_hairpin |
| undef | dlo-miR1147 | 64 | 19 | TCTCGGCAAGTGTGGACAG | no_hairpin |
| undef | dlo-miR1148 | 10 | 24 | ACAACGTGATCAGGGGAGACATGG | no_hairpin |
| undef | dlo-miR1150 | 12 | 23 | AGAGGTAGACATTGATGGCTGGC | no_hairpin |
| undef | dlo-miR1153* | 66 | 20 | AGTGATGCGTACAACTACAA | no_hairpin |
| undef | dlo-miR1154 | 4 | 20 | ATTAGTCAACCCAAAGGCAT | no_hairpin |
| undef | dlo-miR1160 | 102 | 23 | CTGACAATGAATGAAGAGCGGAT | no_hairpin |
| undef | dlo-miR1162* | 300 | 22 | CGGCTTAATTTGACTCAACACG | no_hairpin |
| undef | dlo-miR1169 | 43 | 20 | TGTGGAGTTGATTGCTTGAT | no_hairpin |
| undef | dlo-miR1170 | 18 | 18 | AATAGCCAAACAGGAAGG | no_hairpin |
| undef | dlo-miR1171 | 5 | 24 | TGTGAGTGGAGATGGACTGGACTG | no_hairpin |
| undef | dlo-miR1211* | 226 | 19 | AGGGAGGAGAGGTTTGAAG | no_hairpin |
| undef | dlo-miR1217 | 20 | 19 | AATTTGAGATATGTGCAAG | no_hairpin |
| MIR1220 | dlo-miR1220a | 9 | 24 | TTCCGGTGTTAAGAGAGAAGATAG | no_hairpin |
| MIR1222 | dlo-miR1222e | 22 | 24 | TTGAAGGAAGTTTCAAGTGGTCAT | no_hairpin |
| MIR1223 | dlo-miR1223j | 3 | 20 | TTGAGACGGCTGCACCTCTA | no_hairpin |
| undef | dlo-miR1310 | 190 | 20 | GGCATCGGGGGCGCAACGCC | no_hairpin |
| undef | dlo-miR1312 | 76 | 24 | ATTGGATAGAAAATGGACGGACAT | no_hairpin |
| undef | dlo-miR1313 | 38 | 20 | ACCATATGAAATATTGTTCG | no_hairpin |
| undef | dlo-miR1314 | 3 | 24 | TACGGCCTTGAATAGTTTGGTAGA | no_hairpin |
| undef | dlo-miR1316 | 18 | 19 | GTCCAACACAAACCATTGA | no_hairpin |
| MIR1318 | dlo-miR1318 | 40 | 19 | TCAGGAGAGATAGACACGT | no_hairpin |
| undef | dlo-miR1320 | 163 | 19 | TGTAGGAGGATTTTTATAG | no_hairpin |
| MIR1423 | dlo-miR1423 | 9 | 23 | AAGGCGATACACGTTGGGGCTCA | no_hairpin |
| undef | dlo-miR1424 | 230 | 18 | ATACACGGATGCTGATTG | no_hairpin |
| undef | dlo-miR1426 | 500 | 18 | AGAATATTGATGATATTA | no_hairpin |
| MIR1428 | dlo-miR1428e | 1 | 24 | AATTGCAGCAGGCCCTAATTTTGT | no_hairpin |
| undef | dlo-miR1429 | 38 | 20 | GTTGAGGGTTTGTATAGTTG | no_hairpin |
| undef | dlo-miR1431 | 5 | 21 | TTTTGAGTTGGCCGGACTTGC | no_hairpin |
| undef | dlo-miR1438 | 247 | 18 | AGGGTAATTTATTTTTTA | no_hairpin |
| MIR1444 | dlo-miR1444b | 9 | 20 | ATTCACCTTCGGTCATCTTC | no_hairpin |
| undef | dlo-miR1448 | 34 | 21 | CTTTCCAACACCTCCCATACC | longan-m0012 |
| MIR1508 | dlo-miR1508a | 711 | 18 | TAAAAGGAAGTAGCAGTG | no_hairpin |
| MIR1509 | dlo-miR1509b | 16 | 24 | TTAGAATCAATGAAATGCACGGTT | no_hairpin |
| MIR1510 | dlo-miR1510a | 72 | 20 | GGGAGGAATAGGTAAAACAA | no_hairpin |
|  | dlo-miR2089*-1 | 1 | 24 | AGGAGTGGTGAATAGGTACATGAA | no_hairpin |
|  | dlo-miR2089*-2 | 9 | 24 | AGGAGGTAGGCTGTAATAGTAAAA | no_hairpin |
|  | dlo-miR2089*-3 | 2 | 24 | AGGATGTGGTGTAATTCGGTTAAA | no_hairpin |
|  | dlo-miR2089*-4 | 1 | 24 | AGGATTGGTGGAACACGGTGAAAA | no_hairpin |
|  | dlo-miR2089*-5 | 1 | 23 | GTGGATTTGGTGTAATAGTAAAA | no_hairpin |
|  | dlo-miR2089*-6 | 1 | 24 | AGTGGATTTGGTGTAGTAGTAAAA | no_hairpin |
| undef | dlo-miR1512 | 145 | 18 | TAATAAAATTCTTAAAGT | no_hairpin |
| undef | dlo-miR1513 | 46 | 19 | TGGAGAAAGTCATGATTAG | no_hairpin |
| MIR1514 | dlo-miR1514b | 42 | 21 | GTTCATTTTAAAATAGACAGT | no_hairpin |
| undef | dlo-miR1517 | 5 | 23 | ATTCTTGGTAATGCGTTTGAATA | no_hairpin |
| undef | dlo-miR1519 | 37 | 18 | AGGTTGCAAAATAGTCAT | no_hairpin |
| MIR1520 | dlo-miR1520a | 23 | 24 | TATGAACATGAACACGCAGAGTCA | no_hairpin |
|  | dlo-miR1520d | 10 | 20 | ATCAGAACTGGTACGGACAA | no_hairpin |
|  | dlo-miR1520h | 2 | 24 | GAAAGTCCAATCAACGTCGACATG | no_hairpin |
|  | dlo-miR1520j | 6 | 24 | AAGAACGTGAACAAAGGAAATCAA | no_hairpin |
|  | dlo-miR1520p | 26 | 24 | ATTTGTTGATTGATGATGATGGTT | no_hairpin |
|  | dlo-miR1520q | 12 | 24 | ATTGACCAACAAACAATGACACAG | no_hairpin |
| MIR1521 | dlo-miR1521 | 65 | 20 | TTTAATGGAAATATTGTTGA | no_hairpin |
| undef | dlo-miR1522 | 23 | 21 | ATTTATGGCTAAAAATGAAAT | no_hairpin |
| undef | dlo-miR1523 | 15 | 24 | ATGGGAATGAAAATGTGAGTCTCA | no_hairpin |
| undef | dlo-miR1526 | 5 | 24 | CAGGAAGAGGAAAGATTAAAGCAT | no_hairpin |
| undef | dlo-miR1527 | 6 | 20 | AACTCAATTTTACAAAACCG | no_hairpin |
| undef | dlo-miR1528 | 15 | 24 | AGTAGATTTGTCAAGTATATTAGT | no_hairpin |
| undef | dlo-miR1533 | 118 | 19 | ATCAATCAAATAAAAATGA | no_hairpin |
| undef | dlo-miR1534 | 37 | 22 | TGATTCTTGTGTGAATAGTCAT | no_hairpin |
| undef | dlo-miR1535 | 15 | 20 | CATGTTTGTGGTGATGTTGT | no_hairpin |
| undef | dlo-miR1536 | 6 | 24 | ATAGCTGATGAACAAATGTGTTTA | no_hairpin |
| MIR1846 | dlo-miR1846e | 153 | 20 | CAGAGGAGGACCGGGACCAA | no_hairpin |
| undef | dlo-miR1847 | 13 | 21 | TGCAGTTTGAGTTGATGGACA | no_hairpin |
| undef | dlo-miR1849 | 12 | 19 | ATAGTATCTAGGTTAGTTT | no_hairpin |
| undef | dlo-miR1852 | 55 | 24 | ATATGGATTCACTGGAGATGCAGT | no_hairpin |
| undef | dlo-miR1853 | 8 | 24 | TAAACTTCGGGTGTTCGGTTGCTT | no_hairpin |
| undef | dlo-miR1854 | 10 | 22 | TCAGAATTTGGAGATTTCTGAT | no_hairpin |
| undef | dlo-miR1855 | 5 | 24 | AGCAACGGGAGGTAGCCAAGAGAT | no_hairpin |
| undef | dlo-miR1856 | 35 | 19 | TATTGAAAGACGGATTCGA | no_hairpin |
| undef | dlo-miR1860 | 19 | 22 | ATCTGAAATGCTACTTTTCTCT | no_hairpin |
| MIR1861 | dlo-miR1861n | 5 | 24 | GATCTAGTGGCAGTGAAGACTGAG | no_hairpin |
| undef | dlo-miR1862f | 5 | 24 | GTATGAAGGCTTGGTTTATTTTGG | no_hairpin |
| MIR1863 | dlo-miR1863c | 3 | 21 | AGAAACTGGTGAGCATTAACT | no_hairpin |
| MIR1863 | dlo-miR1863b | 4 | 24 | TAAGCTCTGATACCATGTTAACTT | no_hairpin |
| undef | dlo-miR1863 | 131 | 24 | AAGCTCTGATACCATGTTAGATAC | no_hairpin |
| undef | dlo-miR1863 | 26 | 24 | AAGCTCTGATACCATAGTAGATAT | no_hairpin |
| undef | dlo-miR1864 | 39 | 24 | ATTAGTTAACTGATGGTCAATGGT | no_hairpin |
| undef | dlo-miR1865 | 6 | 24 | TGTTAGTGATGGGATCTACTTGAC | no_hairpin |
| undef | dlo-miR1871 | 59 | 24 | AGGCTCTGATATCATGTTAGTTAT | no_hairpin |
| undef | dlo-miR1872 | 579 | 24 | GAACTGTAACTGAGACGGAGTCAA | no_hairpin |
| undef | dlo-miR1873 | 874 | 23 | TCTGACATGGTATTAGAGCTGGA | no_hairpin |
| undef | dlo-miR1875 | 7 | 24 | AAATGGAAGTAAGATGCAACATAA | no_hairpin |
| undef | dlo-miR1877 | 19 | 24 | AGAAGACATCTGAATGATAGGGAG | no_hairpin |
| MIR1878 | dlo-miR1878 | 8 | 24 | ATTTATATGTTAGATTGAGATATT | no_hairpin |
| undef | dlo-miR1879 | 8 | 24 | GTGTTTGGTGTAGGTGATGGGTGT | no_hairpin |
| undef | dlo-miR1881 | 9 | 24 | AATGTCATTGTAGCTGGTAGGTTT | no_hairpin |
| MIR1882 | dlo-miR1317 | 28 | 24 | AAATGTCTTGGACTGTAATACAAG | no_hairpin |
| undef | dlo-miR1885 | 18 | 24 | ACAGAATGAAAGGTATGATCTCAC | no_hairpin |
| undef | dlo-miR1886 | 9 | 21 | AGGATGGAAATCTATGATTGG | no_hairpin |
| MIR1887 | dlo-miR1887 | 147 | 19 | TACAAGTAGAGATTAAGAG | no_hairpin |
| undef | dlo-miR1888 | 8 | 24 | GAAGTTAAGTATTTGTAGAAGCAA | no_hairpin |
| undef | dlo-miR1917 | 173 | 21 | AATTAATAAAGGTGTTAAATT | no_hairpin |
| undef | dlo-miR1918 | 31 | 22 | TTTGGTTGAGAGTTGGATTCTT | no_hairpin |
| MIR1919 | dlo-miR1919a | 28 | 20 | AGGAGAGTATTGTTGACAGG | no_hairpin |
| undef | dlo-miR2079 | 35 | 24 | ATGATGGATTGATGTTGATGACGA | no_hairpin |
| undef | dlo-miR2081 | 27 | 21 | AGCTGATTTATGTGTCTAGTT | no_hairpin |
| undef | dlo-miR2086 | 14 | 24 | GACATGAATGTCAAACTTGTTGAA | no_hairpin |
| undef | dlo-miR2090 | 2 | 21 | AACTCGATTCAGCATTTTTGA | no_hairpin |
| undef | dlo-miR2091 | 16 | 20 | CATACATTGCATCTAGACTT | no_hairpin |
| undef | dlo-miR2092 | 23 | 20 | AACTGAAGTTCGGAGTTTCT | no_hairpin |
| undef | dlo-miR2093 | 113 | 18 | ACTATTCCAATTAACGAT | no_hairpin |
| undef | dlo-miR2094 | 7 | 23 | ACAAGCTGTTGGCATCCATCGTC | no_hairpin |
| undef | dlo-miR2095 | 11 | 25 | CGATAATGTTTACGAGTAGAATGAG | no_hairpin |
| undef | dlo-miR2096 | 55 | 24 | CCTCGAGAGGGAGATCGGCGTGGA | no_hairpin |
| undef | dlo-miR2097 | 42 | 24 | AGAAGATGAAGGACAGCAGGGAAG | no_hairpin |
| undef | dlo-miR2098 | 285 | 20 | CGGTTGCCAAGTGGAGTGCG | no_hairpin |
| undef | dlo-miR2099 | 68 | 18 | AAAATCTGTATCGTTATT | no_hairpin |
| undef | dlo-miR2101 | 42 | 21 | ATTGATACTCAAGTACATTGT | no_hairpin |
| undef | dlo-miR2103 | 1 | 24 | TTTCCCTCTTCCGGCGTCGGCTCC | no_hairpin |
| undef | dlo-miR2105 | 16 | 24 | TTGTGATGTTGAAATGTATTGCAT | no_hairpin |
| MIR2088 | dlo-miR2088a* | 39 | 18 | CCATGTATCTAGGTCATA | no_hairpin |
| MIR2108 | dlo-miR2108a | 39 | 19 | TTATGTGTTGGTTTTTGGG | no_hairpin |
| MIR2111 | dlo-miR2111a* | 17 | 18 | GCCTCGGGATACGGATTA | no_hairpin |
| MIR2118 | dlo-miR1859 | 3 | 24 | TCTCGCTATGACGTCAAGTTCCAA | no_hairpin |
|  | dlo-miR1869 | 9 | 23 | TAGAAAACAATAGGATGGAGGGA | no_hairpin |
|  | dlo-miR2118e | 1418 | 24 | GTTCCGTGATCGTTTCCATTCCTA | no_hairpin |
| MIR2119 | dlo-miR2199 | 29 | 21 | TCTAAAGAGAATTGTAGGGGA | no_hairpin |
| undef | dlo-miR2199 | 13997 | 19 | TGATAACTCGACGGATCGC | no_hairpin |
| MIR2275 | dlo-miR2275a | 59 | 24 | AAGAGTTGGAGAAAAGCATAACAC | no_hairpin |
| MIR2592 | dlo-miR2592s | 7 | 23 | GAAAATGCTTGAGTTATGATGTT | no_hairpin |
| MIR2593 | dlo-miR2593a | 13 | 24 | GTTAAATTGTAGATGAACCAGAAT | no_hairpin |
| MIR2594 | dlo-miR2594a | 25 | 24 | ACATGGACCAAGGAATGACCAGAG | no_hairpin |
| undef | dlo-miR2595 | 4 | 22 | TATATTTTCTTCTTTGACTGTT | no_hairpin |
| undef | dlo-miR2596 | 3 | 23 | TTTATTTCATTATTATCCACACA | no_hairpin |
| undef | dlo-miR2597 | 19 | 20 | TATGGTATTGTCAGATTTGA | no_hairpin |
| undef | dlo-miR2604 | 10 | 21 | TAATTTTTTTGTTGGGATGCT | no_hairpin |
| undef | dlo-miR2607 | 79 | 19 | AGAGATAATGGATAAGTGT | no_hairpin |
| undef | dlo-miR2608 | 4 | 21 | GTTGTACATAATACTTAATCT | no_hairpin |
| MIR2610 | dlo-miR2610a | 31 | 20 | AAATTAGACTTGTTGGCATT | no_hairpin |
| undef | dlo-miR2611 | 32 | 24 | ATTGTAGATCAAGTGTTTGATGAA | no_hairpin |
| MIR2612 | dlo-miR2612a | 167 | 18 | TGAGGTTCAACTAGTAAG | no_hairpin |
| undef | dlo-miR2613 | 54 | 22 | CGTCGCCGGTCGGACCAATGGT | no_hairpin |
| MIR2615 | dlo-miR2615a | 1 | 22 | GCTGATCGCATTTTTAAAAGGC | no_hairpin |
| undef | dlo-miR2616 | 82 | 24 | ATTGAGTGTTTGGTTCGGGTCGGT | no_hairpin |
| MIR2617 | dlo-miR2617a | 13 | 20 | TGATTCTAGCATGCGCCGTT | no_hairpin |
| undef | dlo-miR2619 | 9 | 24 | ACATAGGAGGCTGGATTTGCTATT | no_hairpin |
| undef | dlo-miR2620 | 28 | 24 | GCTTCTGATAGACATCCGGCTTGT | no_hairpin |
| undef | dlo-miR2621 | 9 | 24 | AGACTTGTGGTTAGGATTTGTGCT | no_hairpin |
| undef | dlo-miR2622 | 5 | 21 | TTTGTGCCATCGTCGATCTTA | no_hairpin |
| undef | dlo-miR2628 | 452 | 21 | TATGGATAAGATGATGAGTAA | no_hairpin |
| undef | dlo-miR2634 | 54 | 19 | TTTATTTCAGTTTGATGTG | no_hairpin |
| undef | dlo-miR2639 | 3 | 20 | TAGTCGGCTTACCCTCCCTT | no_hairpin |
| undef | dlo-miR2641 | 31 | 18 | GTTTGATCCTTGAGGTTA | no_hairpin |
| undef | dlo-miR2642 | 41 | 19 | AACTTTCATCAAATCATGT | no_hairpin |
| undef | dlo-miR2643 | 31 | 19 | TTTGGGACAGAGATTGGAA | no_hairpin |
| MIR2629 | dlo-miR2087 | 6 | 22 | GAATGTAAAGAACTCGGTGAAG | no_hairpin |
| MIR2644 | dlo-miR2644a | 31 | 19 | CTTCGATATGATGGTGTGT | no_hairpin |
| MIR2646 | dlo-miR2646a | 9 | 21 | ACATTAATTTCGTGATGATGT | no_hairpin |
| undef | dlo-miR2648 | 9 | 20 | TGCCGATGGGAATAGACAGT | no_hairpin |
| undef | dlo-miR2651 | 9 | 20 | TTTGATTGGATGCACTGCTA | no_hairpin |
| MIR2652 | dlo-miR2652a | 2 | 23 | TAGGCTAGTGTGCATGAAGGATT | no_hairpin |
| MIR2653 | dlo-miR2653a | 58 | 21 | TCGACGTTGCTGTGAACAAGT | no_hairpin |
| undef | dlo-miR2654 | 19 | 20 | ATTTACGGACAAAGATGTGG | no_hairpin |
| MIR2657 | dlo-miR2657a | 146 | 22 | TGTTCTTCCAGTCGATTTTGTT | no_hairpin |
| undef | dlo-miR2658 | 259 | 18 | GTGGACCTTGTAGATGAC | no_hairpin |
| undef | dlo-miR2661 | 107 | 19 | TAGGTTTGGAGAATGGTCA | no_hairpin |
| undef | dlo-miR2662 | 2 | 24 | GAGTATGAATACATGGAACCGAAT | no_hairpin |
| undef | dlo-miR2663 | 44 | 20 | TCGAGTGAGGGCTTACAATT | no_hairpin |
| undef | dlo-miR2666 | 15 | 21 | GAAAGTGAGGAACTCAAGGAA | no_hairpin |
| undef | dlo-miR2667 | 10 | 18 | CCTTGATCTGAGGTTATC | no_hairpin |
| undef | dlo-miR2669 | 3 | 24 | AAGAGTTCAGGTCTTACATTTATC | no_hairpin |
| MIR2670 | dlo-miR2670a | 3 | 20 | AAAAAGGTTGCTACTATCTT | no_hairpin |
| MIR2655 | dlo-miR2671j | 7 | 21 | TCAACAAGGTTCGTTTCGTCC | no_hairpin |
| undef | dlo-miR2672 | 1 | 24 | ATAAATCGACTCAAGTGGGTACTA | no_hairpin |
| MIR2673 | dlo-miR2673a | 4 | 20 | CTCTTCCTCTTCCTCGTCTC | no_hairpin |
| MIR2675 | dlo-miR1525 | 71 | 19 | AGGGTAATTAATTTTTTAG | no_hairpin |
| MIR2676 | dlo-miR2676a | 140 | 20 | ATTATTTGGATAATGAATTT | no_hairpin |
| undef | dlo-miR2677 | 5 | 22 | TTTGATTGATATTTTAGTAGAT | no_hairpin |
| undef | dlo-miR2678 | 29 | 21 | TGAAATTGTTGAAGATGTCCT | no_hairpin |
| MIR2863 | dlo-miR2863b | 20 | 21 | TTTGATTTATTTGGACAGAGA | no_hairpin |
| undef | dlo-miR2864 | 467 | 20 | TATGTTTTGATTGATGGGTA | no_hairpin |
| undef | dlo-miR2868 | 38 | 22 | TTCGGTTTTTTATAGTAGAAGA | no_hairpin |
| undef | dlo-miR2870 | 43 | 19 | TAAAGATTGGTGAGACAAA | no_hairpin |
| MIR2873 | dlo-miR2873b | 7 | 22 | TTGAGACTTGAGATTGGTCAGG | no_hairpin |
| undef | dlo-miR2877 | 21 | 23 | TCTGATATCTGCAGTTTGGGCCT | no_hairpin |
| undef | dlo-miR2878 | 9 | 23 | AACATCTATAAAATTTAGGATCG | no_hairpin |
| undef | dlo-miR2905 | 4 | 24 | TACATGCTCTGTGGCACAAAGGCA | no_hairpin |
| undef | dlo-miR2911 | 11279 | 18 | GCCGGGGGACGGACTGGG | no_hairpin |
| undef | dlo-miR2913 | 4 | 23 | GAGGTTGGGCTATTGCAAGTGAG | no_hairpin |
| undef | dlo-miR2916 | 7853 | 24 | GTTGGGGGCTCGAAGACGATCAGA | no_hairpin |
| undef | dlo-miR2920 | 1 | 24 | AATACTAACAATTAACATTTGCAA | no_hairpin |
| undef | dlo-miR2921 | 26 | 24 | AAGAGACTTAATAATTACTTTAAC | no_hairpin |
| undef | dlo-miR2922 | 7 | 20 | AATAAGTTTTTACGCGAATT | no_hairpin |
| undef | dlo-miR2923 | 10 | 24 | AGACAAACAATATGAGCATAACAA | no_hairpin |
| undef | dlo-miR2928 | 655 | 23 | ATGAAGACGACAATTCTGTGTTG | no_hairpin |
| undef | dlo-miR2930 | 12 | 24 | CTTCTCTCTCTCTCTCTCGTGGCC | no_hairpin |
| undef | dlo-miR2931 | 46 | 19 | CTTTATGGTTTGTCAAAAG | no_hairpin |
| MIR2933 | dlo-miR2933a | 54 | 19 | GAAATGGAGAGGAAATTTG | no_hairpin |
| undef | dlo-miR2936 | 39 | 24 | CTTGAGAGAGAGACAGAGACAGAG | no_hairpin |
| undef | dlo-miR2937 | 36 | 21 | ATAGAGAGCTGTAAGGAGGTC | no_hairpin |
| undef | dlo-miR2938 | 224 | 21 | ATCTTCTGAGAAGGGTTCGAG | no_hairpin |
| MIR2934 | dlo-miR782 | 1 | 23 | ACAAGCAACTCTTGGATCTTCTT | no_hairpin |
| undef | dlo-miR3433 | 24 | 20 | TCAAGTCTTGCTATACCCAC | no_hairpin |
| MIR3434 | dlo-miR3434 | 3 | 24 | TCAAAATCAGAAGATGTCAACCTA | no_hairpin |
| undef | dlo-miR3435* | 14 | 19 | TGGCATATTTCTTACTTCC | no_hairpin |
| undef | dlo-miR3436* | 19 | 25 | ACAACAACAAGTTTTTGGCGCCGTT | no_hairpin |
| undef | dlo-miR3437 | 9 | 19 | AAAAAACAAGGAGTCAGGA | no_hairpin |
| undef | dlo-miR3438 | 17 | 21 | TCAAAGATATAAGTATGAAGC | no_hairpin |
| undef | dlo-miR3439* | 6 | 21 | TTGGGTTTGGAAAATCAAGAA | no_hairpin |
| MIR3440 | dlo-miR3440b | 77 | 18 | TAATTGGTCAAGGAAGCT | no_hairpin |
| undef | dlo-miR3441* | 433 | 22 | CAAGAAATTGATAGTCGTTTGT | no_hairpin |
| undef | dlo-miR3442 | 106 | 18 | TTCGGATCAAAATGGTGT | no_hairpin |
| MIR3444 | dlo-miR3444 | 23 | 19 | TTGGGAGACGATGGATCTA | no_hairpin |
| undef | dlo-miR3445* | 1 | 24 | GGTCTTTTCTCCACTATGCAAGCA | no_hairpin |
| undef | dlo-miR3447 | 48 | 23 | ATTTCGATTGTGTTTGGTATGAA | no_hairpin |
| undef | dlo-miR3448 | 19 | 24 | TATCGATGGATTTTGTTTTGGGTC | no_hairpin |
| undef | dlo-miR3449* | 9 | 24 | AGATAGAGAAAGACAATTTGGATA | no_hairpin |
| undef | dlo-miR3451* | 1 | 24 | TGGTAGACACTTGCCTTCCGCAAA | no_hairpin |
| undef | dlo-miR3452* | 15 | 19 | TTCCTATGATGATCCATGT | no_hairpin |
| undef | dlo-miR3453 | 4 | 21 | TCTCTCTCGATCGCCGCCATG | no_hairpin |
| MIR3454 | dlo-miR3454a* | 56 | 19 | CACCATCGGATCTAGGTAG | no_hairpin |
| undef | dlo-miR3458 | 17 | 19 | GCTCCAAAAGATCGTTCCT | no_hairpin |
| undef | dlo-miR3465* | 36 | 23 | CAGCTGAAGATTCTATCCATGAC | no_hairpin |
| undef | dlo-miR3467* | 3 | 21 | TGCTGGACTAGCCAAGACCTA | no_hairpin |
| undef | dlo-miR3509 | 3 | 24 | ATACTATGGAGAGCCTTAGAAGGA | no_hairpin |
| undef | dlo-miR3510 | 4 | 24 | ATTAACCATCTTGAGAGTCACTGA | no_hairpin |
| undef | dlo-miR3512 | 62 | 21 | GCAAGTGATGACAAAATAGAC | no_hairpin |
| undef | dlo-miR3513 | 7 | 24 | TTGATAAGATATGAAATTGGATAA | no_hairpin |
| undef | dlo-miR3515 | 68 | 18 | CATGTAGAAAAGCGGTAT | no_hairpin |
| undef | dlo-miR3516 | 4 | 24 | AGCTGGGTGAATTGGACAAGCAAG | no_hairpin |
| undef | dlo-miR3519 | 22 | 21 | TAATGAACTGACAGCTTTTCA | no_hairpin |
| undef | dlo-miR3520 | 110 | 24 | AGGTGGATGGATGGTATCTTAGTC | no_hairpin |
| MIR3522 | dlo-miR3522a | 24 | 23 | AGAGACCAAATGAAGCCAGATGA | no_hairpin |
| undef | dlo-miR3623* | 2 | 24 | TTCACAAGTTCGTTCAAGCACCAC | no_hairpin |
| undef | dlo-miR3624* | 48 | 20 | ATATATGCTGCAGTCTTTAG | no_hairpin |
| undef | dlo-miR3625 | 26 | 23 | CGGGAGCATGACTAGTAGAAGCC | no_hairpin |
| undef | dlo-miR3627 | 521 | 22 | TCTGTCGCAAGAGAGATGGCAC | no_hairpin |
| undef | dlo-miR3629a | 50 | 24 | GAGCTGCTGGAGAGAAAATGAGGA | no_hairpin |
| undef | dlo-miR3630* | 43 | 19 | GGGAATCTCTCTGATGCAC | no_hairpin |
| MIR3631 | dlo-miR3631a* | 17 | 24 | TATGATGATGAGATGATGTAACAA | no_hairpin |
| undef | dlo-miR3632* | 6 | 24 | GTAGAATTGAGGGCGATGGAAAGG | no_hairpin |
| undef | dlo-miR3633a | 23 | 20 | GAAATGGATGGTAGGAGATC | no_hairpin |
| undef | dlo-miR3636 | 105 | 23 | GCCATGTCGGACAAGCAAGTGGA | no_hairpin |
| undef | dlo-miR3637* | 11 | 24 | AGTTCATTATTTTGTTTTGTCGGA | no_hairpin |
| undef | dlo-miR3639 | 30 | 22 | ATTACTCTAAAGGCTACAAGCT | no_hairpin |
| undef | dlo-miR3702 | 4 | 24 | AATTCTTGGTGTACATTATTCTGC | no_hairpin |
| undef | dlo-miR3704 | 2 | 24 | GGTCATAAGTGAGTTGGAAAGAAA | no_hairpin |
| undef | dlo-miR3705 | 363 | 21 | GTAAGGTGATTTGATCTGGAT | no_hairpin |
| undef | dlo-miR3706 | 138 | 22 | ATTGGGAGAAAATGGATAAAGA | no_hairpin |
| undef | dlo-miR3707 | 4 | 21 | TTGAGTCTGCCATTATGCTTG | no_hairpin |
| undef | dlo-miR3711 | 44 | 24 | TGGCGCTAGAAGGAGGGGCTCATC | no_hairpin |
| undef | dlo-miR3712 | 9 | 24 | TTTGATCAAGATCAGCTACTCATT | no_hairpin |
| MIR3932 | dlo-miR3932a | 27 | 21 | AACTTTGGAAGACAATCGTAG | no_hairpin |
| undef | dlo-miR3933 | 1 | 21 | AGAAGCAAAAATGCAGATCGG | no_hairpin |
| undef | dlo-miR3933 | 1 | 21 | AGTAAGCAAAAAACAACTCGG | no_hairpin |
| undef | dlo-miR3946 | 94 | 24 | TGGTAGAGAAAGTAGAGAGGAGAC | no_hairpin |
| undef | dlo-miR3948 | 8 | 22 | TGGTGGGAGTGTGGATAAGGTG | no_hairpin |
| undef | dlo-miR3949 | 133 | 19 | TGATGTTTGGAAAAATGAG | no_hairpin |
| undef | dlo-miR3951 | 18 | 24 | AAGATAAAGATGACGAAGAAAGAA | no_hairpin |
| undef | dlo-miR3954 | 305540 | 22 | TTGGACAGAAAAATCACGGTCA | no_hairpin |
| undef | dlo-miR3979 | 5 | 20 | ATCCTCATCTCCCTTAAGGC | no_hairpin |
| undef | dlo-miR3981 | 30 | 18 | AGTATTAGATACGTCTTG | no_hairpin |
| undef | dlo-miR3982 | 3 | 22 | AGCTTGCCTACATGGAGCACCA | no_hairpin |
| MIR4208 | dlo-miR4248a | 12 | 20 | AATTTTATTTTGTGGCATCT | no_hairpin |
| MIR4221 | dlo-miR4221 | 24 | 24 | AAGGGTCAACAAGTAGTGAAAGTA | no_hairpin |
| undef | dlo-miR4223 | 2 | 24 | ACGGTAATTTGATAATACTAAACA | no_hairpin |
| undef | dlo-miR4232 | 6 | 24 | TGGAAACTTTATTAGGATGTGTGC | no_hairpin |
| undef | dlo-miR4233 | 4 | 22 | GACATCATCAATCATCAATCCA | no_hairpin |
| undef | dlo-miR4234 | 31 | 24 | CAACTTAAAAATCATTGGGCTAAT | no_hairpin |
| undef | dlo-miR4235 | 6 | 24 | TATCTGACAAGACACTACAACCCT | no_hairpin |
| undef | dlo-miR4237 | 52 | 22 | CAAACGTAAACGTTAAAATCGA | no_hairpin |
| undef | dlo-miR4246 | 4 | 24 | AATCTAATTTTCATTGGCTATAAT | no_hairpin |
| undef | dlo-miR4249 | 12 | 23 | TGAATTTGTTGAAGATTGAGTTA | no_hairpin |
| undef | dlo-miR4342 | 13 | 24 | AATCGACTGAAGAATGTGAGGTGG | no_hairpin |
| MIR4343 | dlo-miR4343a | 21 | 24 | GAAAAACTTACTTGGATCGTTGAT | no_hairpin |
| undef | dlo-miR4344 | 8 | 21 | AAAACATTGTAAGACGTTGCT | no_hairpin |
| undef | dlo-miR4345 | 40 | 20 | TAAGACGGAATTCACAGATC | no_hairpin |
| undef | dlo-miR4346 | 3 | 24 | AAAGACCAGACGAGCGAAGCTGCT | no_hairpin |
| undef | dlo-miR4347 | 1 | 24 | AACTACTTGACGGATCAGTTGATA | no_hairpin |
| undef | dlo-miR4348 | 326 | 24 | TAACTCTGATAAGATGGTTGACTT | no_hairpin |
| MIR4349 | dlo-miR4349 | 10 | 24 | TTATGTGGCTGAGAAAGACAAGGA | no_hairpin |
|  | dlo-miR4379 | 14 | 23 | AGAGTTTGTACTGTGATGAGGCC | no_hairpin |
| undef | dlo-miR4350 | 6 | 24 | TAACAATGAATTTTGTCTCGTTGG | no_hairpin |
| undef | dlo-miR4351 | 5 | 24 | ATGGGATTTCACTTATGGAGTTGG | no_hairpin |
| undef | dlo-miR4352b | 64 | 22 | GTGAAAGTAGACTTCAAGACGG | no_hairpin |
| MIR4359 | dlo-miR4359a | 2 | 23 | AGACAGTGACTCTCACAGCGGTT | no_hairpin |
| undef | dlo-miR4361 | 16 | 21 | GAAGAACTTGACGGATCAACT | no_hairpin |
| undef | dlo-miR4362 | 17 | 20 | CCTTAGGACAACAGCATTAG | no_hairpin |
| undef | dlo-miR4365 | 1 | 23 | AAGAACTGTCTTCTCCAGATGCA | no_hairpin |
| undef | dlo-miR4366 | 25 | 24 | TACGTTAAGGTAGAGATTTGTTGA | no_hairpin |
| undef | dlo-miR4369 | 14 | 23 | GGATCAACTGACCAGTAGATGGA | no_hairpin |
| MIR4371 | dlo-miR4371a | 21 | 24 | AGTGATGACATACAAGTAGAATGT | no_hairpin |
| MIR4372 | dlo-miR4372 | 78 | 22 | TAAAAACGTGACATGTGACATA | no_hairpin |
|  | dlo-miR4381 | 67 | 24 | TTTGACGGTAAACGGTGAAAAGAC | no_hairpin |
| undef | dlo-miR4373 | 5 | 24 | AAGTTGACGTTACGTACTTATTGA | no_hairpin |
| MIR4374 | dlo-miR4374a | 4561 | 23 | TAAGACGTGTACGATGATGAGCA | no_hairpin |
| undef | dlo-miR4377 | 227 | 24 | ACGTCATGTTGAATTGGAAGACGA | no_hairpin |
| MIR4378 | dlo-miR4378a | 397 | 21 | ATGGAATGCTTAGAATGGTGA | longan-m0006 |
| MIR4380 | dlo-miR4380b | 5 | 24 | TTGGTCAGACCGGATTGTTCGATC | no_hairpin |
| undef | dlo-miR4382 | 3 | 24 | GATATGTTAACTGATGCATGGATA | no_hairpin |
| undef | dlo-miR4383 | 41 | 24 | TGATTGGATTCTCAGTGAACGGTT | no_hairpin |
| undef | dlo-miR4384 | 1 | 24 | AATCAAACTGCATTCAAAGTATCA | no_hairpin |
| undef | dlo-miR4385 | 13 | 24 | ACGATGTAGCAAAGTAGATTCGGT | no_hairpin |
| undef | dlo-miR4386 | 10 | 22 | TGAAGCTTCTGGAGGACATGCA | no_hairpin |
| MIR4387 | dlo-miR4387d | 14 | 24 | ATGTCACTGTACTTAGCATGATAA | no_hairpin |
| undef | dlo-miR4387b | 2 | 24 | AAGGTGTTGATGGCATGAGACTTG | no_hairpin |
| undef | dlo-miR4388 | 31 | 23 | AATTTAAGGACTCAAATTGAAGA | no_hairpin |
| undef | dlo-miR4389 | 14 | 24 | CGATCGGACGCGATCCATCGGAAA | no_hairpin |
| undef | dlo-miR4391 | 6 | 24 | TTGGCTAAAGAACTAAGCAAGAAG | no_hairpin |
| undef | dlo-miR4393b | 11 | 23 | TGGAAAAGGTAAGAGATGAAGCC | no_hairpin |
| undef | dlo-miR4397 | 339 | 20 | TGTCAAAGATTGGCGGATCA | no_hairpin |
| undef | dlo-miR4398 | 115 | 22 | TGTAGTGGAGGATGAAGACGAA | no_hairpin |
| undef | dlo-miR4399 | 11 | 24 | TTAACGAAGAAAGGCTGAAAAGAC | no_hairpin |
| undef | dlo-miR4400 | 11 | 23 | TAGAAAAATTCTGGAAGACGTTA | no_hairpin |
| undef | dlo-miR4401 | 36 | 23 | AAACTGTCGTGGAAGTAGGCATT | no_hairpin |
| undef | dlo-miR4403 | 59 | 24 | ACGACCCGAACACGACACGATAAC | no_hairpin |
| undef | dlo-miR4404 | 7 | 24 | AATTAGTAGGAAGATGCGGATCAA | no_hairpin |
| undef | dlo-miR4406 | 5 | 24 | ATGATTTCTGAGAGACGGGTGTTA | no_hairpin |
| undef | dlo-miR4407 | 6 | 23 | AGTAGGAAAGCAGCATTGTAACC | no_hairpin |
| undef | dlo-miR4411 | 2 | 24 | TTCCTGTGAACTAATTTGTCGGTG | no_hairpin |
| undef | dlo-miR4413 | 141 | 18 | ATAAATTGTAAGTCAATG | no_hairpin |
| undef | dlo-miR4414 | 1418 | 21 | AGCTGCTGAGTCGTTGGTTCA | no_hairpin |
| undef | dlo-miR4415 | 3 | 24 | AAGTTTGATGAGAATGCAATGTGG | no_hairpin |
| undef | dlo-miR5012 | 2 | 22 | TTTTTACTGCTACTGTTTGTCC | no_hairpin |
| undef | dlo-miR5014 | 704 | 18 | TTGACAAATTTGAGGACG | no_hairpin |
| MIR5015 | dlo-miR5015b | 47 | 21 | TGTGTTGTGTTGGTGATAGTG | no_hairpin |
| undef | dlo-miR5016 | 2 | 24 | TTCATTGTAGCTGATTCTTGGAAA | no_hairpin |
| undef | dlo-miR5018 | 2 | 24 | TTAAGAGCTTCACCATGACTCAAT | no_hairpin |
| MIR5020 | dlo-miR5020a | 70 | 19 | TGGAAAGATGAGACTTGCT | no_hairpin |
| undef | dlo-miR5021 | 222 | 23 | TGGGAGAAGAAGAACAAGAAGAA | no_hairpin |
| undef | dlo-miR5023 | 11 | 21 | ATTGGTTAGTGGAAAGGTGAC | no_hairpin |
| undef | dlo-miR5025 | 52 | 24 | ACTGTATATTGTGATAGTCAGACA | no_hairpin |
| undef | dlo-miR5026 | 64 | 21 | GACGTTAAGATCGTGACACGT | no_hairpin |
| undef | dlo-miR5028 | 155 | 21 | AAATGGTGTTTATGCTACAGT | longan-m0001 |
| undef | dlo-miR5029 | 7 | 24 | AATGAGATAGAAGCACCTGCAAAA | no_hairpin |
| undef | dlo-miR5048 | 16 | 24 | ATTTAGGCATGTTTTGAGGTCTAA | no_hairpin |
| MIR5067 | dlo-miR5049 | 145 | 21 | TCTTAAATACTGTAGTTGAGG | no_hairpin |
|  | dlo-miR5067 | 20 | 21 | TCAGGACAACTTCTATGGCAT | no_hairpin |
|  | dlo-miR5175a | 21 | 24 | AAAGAATTTAGGAACGAGGTAGAA | no_hairpin |
| undef | dlo-miR5052 | 462 | 21 | ACAGTCATGGACGTAGGCATA | no_hairpin |
| undef | dlo-miR5054 | 344 | 20 | GTTCCCCACAGACGGCGCCA | no_hairpin |
| undef | dlo-miR5056 | 9 | 23 | AGGAAGAACCGGCCTATAAGACA | no_hairpin |
| undef | dlo-miR5057 | 18 | 19 | AATTTCAAATCGTCTTGCA | no_hairpin |
| undef | dlo-miR5058 | 16 | 24 | AAAGATTGAGAGGCATGAAAACCA | no_hairpin |
| undef | dlo-miR5059 | 284 | 22 | TCGTTCCTGGGCAGCAACACCA | no_hairpin |
| undef | dlo-miR5066 | 7 | 19 | AATGTTAACTTGAGTGCCT | no_hairpin |
| undef | dlo-miR5069 | 21 | 23 | TGGGTTATTGAACTTTTACCAAC | no_hairpin |
| undef | dlo-miR5071 | 2 | 21 | TCAAGACATCATATGTGTTCA | no_hairpin |
| undef | dlo-miR5072 | 7 | 21 | CGTTCCCCAGTGGAGTCGCCA | no_hairpin |
| undef | dlo-miR5074 | 34 | 20 | GAAAGCCACCGTGGGATGCT | no_hairpin |
| undef | dlo-miR5077 | 1055 | 18 | TTCACGTCGGGTTCACCA | no_hairpin |
| undef | dlo-miR5078 | 2 | 19 | GCTCGTCCGACGTGGCATT | no_hairpin |
| undef | dlo-miR5079 | 180 | 19 | TTTGGATTGTTTTTGGTAC | no_hairpin |
| undef | dlo-miR5081 | 12 | 24 | AAATGTTGTAGCAAGTTGATATGT | no_hairpin |
| undef | dlo-miR5083 | 39 | 21 | AGACTACAATTATCTGATCTA | no_hairpin |
| undef | dlo-miR5085 | 22 | 19 | TAGGAATGTTTTGTGGCTG | no_hairpin |
| undef | dlo-miR5137 | 38 | 19 | AGCGAGAGAAGAACGGGCT | no_hairpin |
| undef | dlo-miR5138 | 16 | 19 | ATAGAAATCGTTTGGCGCT | no_hairpin |
| undef | dlo-miR5139 | 208 | 21 | CGTAACCTGGCTCTGATACCA | no_hairpin |
| undef | dlo-miR5140 | 22 | 20 | GATGGTGAAGATTCTGGTAG | no_hairpin |
| undef | dlo-miR5141 | 17 | 24 | AGACCCACGACAACGACAGATGAA | no_hairpin |
| undef | dlo-miR5142 | 26 | 20 | AAATTGTGATTGATAAGTGT | no_hairpin |
| undef | dlo-miR5163 | 19 | 20 | TTAGGTATTTGGTTTGAGTG | no_hairpin |
| undef | dlo-miR5166 | 1 | 21 | TGCCACCAGGTTTCGATCTCT | no_hairpin |
| undef | dlo-miR5168 | 28 | 19 | TGTGGACATCTGACCAGGC | no_hairpin |
| undef | dlo-miR5169 | 19 | 24 | TTTGAGGACAAGTTTGTAGAGACA | no_hairpin |
| undef | dlo-miR5170 | 146 | 19 | TCAACAAGTTGGTGAACGT | no_hairpin |
| undef | dlo-miR5171 | 20 | 19 | ACATAATATGGGAGGATAA | no_hairpin |
| undef | dlo-miR5172 | 19 | 24 | GATGCTACTGAGACTCCTCGGCAT | no_hairpin |
| undef | dlo-miR5176 | 90 | 24 | TGTTGATTGATTGGCAGATAGAAT | no_hairpin |
| undef | dlo-miR5177 | 3 | 21 | TGTAGCATGTAAAACAGACGT | no_hairpin |
| undef | dlo-miR5182 | 46 | 21 | ATGATGATCTTTGGAACACGC | no_hairpin |
| MIR5185 | dlo-miR5185a | 24 | 20 | ATCTATTCATTTTTCAAGAT | no_hairpin |
| undef | dlo-miR5198 | 3 | 23 | GGGTAAAAAAGAAGTTGAGGGAG | no_hairpin |
| undef | dlo-miR5200 | 6 | 21 | TGAAGATGACGCTCAAGGCTT | no_hairpin |
| undef | dlo-miR5201 | 10 | 23 | AGGGCAAGGCAAACTGCATCACA | no_hairpin |
| undef | dlo-miR5202 | 51 | 21 | TTACGTGAGTTGATTGTCTGA | no_hairpin |
| undef | dlo-miR5203 | 5 | 24 | ACTTATTTGGACTGACGAGAGGGA | no_hairpin |
| undef | dlo-miR5300 | 27 | 24 | TCCCCAGACCTCGGACATTCCAAC | no_hairpin |
| undef | dlo-miR5301 | 193 | 24 | TGTGCGTTGGGGTGGAAAAGCATT | no_hairpin |
| **non-conserved longan miRNA** | | | | | |
| family | gene name | count(total) | length | small RNA sequence | hairpin |
| MIR161 | dlo-miR161.2 | 2 | 21 | CAATTGCATTGAAAGCTGCTA | no_hairpin |
| MIR163 | dlo-miR163 | 32 | 24 | TTGAAGAAGACTGTGTAATTCGAT | no_hairpin |
| MIR171 | dlo-miR170* | 9 | 21 | TATTGGCCTGGTTCACTCAGA | no_hairpin |
| undef | dlo-miR418 | 73 | 24 | TGAGATGTGATGGTGACACTGACC | no_hairpin |
| undef | dlo-miR443 | 14 | 24 | ATCACAATACAATAGACATCTGCT | no_hairpin |
| MIR447 | dlo-miR447a | 40 | 22 | TTGTGAAGAAATTGATAGTAGT | no_hairpin |
| undef | dlo-miR472a | 108 | 21 | TTCCCTACTCCACCCATGCCA | no_hairpin |
| MIR476 | dlo-miR476a | 14 | 20 | AGAAGCTCTTCTTTGCAAAG | no_hairpin |
| MIR477 | dlo-miR473a | 3 | 20 | ATCTCCTCAAGAGCTTCTCA | no_hairpin |
|  | dlo-miR477a | 3 | 19 | ATAGCCTTTATGGAGAGGA | no_hairpin |
| MIR482 | dlo-miR482 | 13 | 21 | TTGCCAATTCCACCCATGCCA | no_hairpin |
|  | dlo-miR482a | 6 | 22 | TCTTTCCAACACCTCCCATACC | longan-m0012 |
|  | dlo-miR482b | 43 | 22 | TCTTCCCTACTCCACCCATGCC | no_hairpin |
|  | dlo-miR2948 | 362 | 20 | TGTGGGAGAATTGGACAGAA | no_hairpin |
| undef | dlo-miR529 | 881 | 21 | ACAACGAGAGAGAGCACGCTT | no_hairpin |
| MIR529 | dlo-miR529d | 6 | 18 | AGAAGAGAGTGAGCACAC | longan-m0005 |
| MIR535 | dlo-miR535a | 9232 | 21 | TGACAACGAGAGAGAGCACGC | no_hairpin |
| MIR827_3 | dlo-miR827 | 753 | 21 | TTAGATGACCATCAACAAACG | no_hairpin |
|  | dlo-miR827* | 18 | 18 | TTGTTGATTGCCATCTAC | no_hairpin |
| MIR828 | dlo-miR828* | 6 | 24 | AGATTTCATTTGATTAGCAAGCAA | no_hairpin |
| MIR858 | dlo-miR858 | 46 | 21 | TTCGTTGTCTGTTCGACCTTG | no_hairpin |
| undef | dlo-miR894 | 6039 | 20 | GTTTCACGTCGGGTTCACCA | no_hairpin |
| MIR902 | dlo-miR902 | 123 | 24 | AGAAGATCTTGACAACTCATATAC | no_hairpin |
| undef | dlo-miR1134 | 5 | 24 | CGTCGAACAACAACAAGATGAAGG | no_hairpin |
| MIR1507 | dlo-miR1507a | 6 | 24 | CTCTAATTCCAATACCATCGTCGA | no_hairpin |
|  | dlo-miR1507* | 8 | 24 | AGAAGATTGTATGGAAGAGAAAAT | no_hairpin |
| MIR1858 | dlo-miR1858a | 3 | 24 | GAGGGAGGAACGGAGTGAGGGATC | no_hairpin |
