# Supplementary material for: Comparative Analysis Reveals Dynamic Changes in miRNAs and Their Targets and Expression during Somatic Embryogenesis in Longan (Dimocarpus longan Lour.)
Source: PLoS One. 2013 Apr 11;8(4):e60337. doi: 10.1371/journal.pone.0060337 (PMC3623967; doi:10.1371/journal.pone.0060337)
Supplement: Supporting Information S1 File containing the following supporting information tables — Table S1. Coserved and non-coserved longan miRNAs. Table S2. Longan-known miRNA targets by predicition method. Table S3. Identifiction targets for longan miRNAs by Degradome sequencing. Table S4. The consensus targets for longan miRNAs obtaining from prediction and degradome analysis. Table S5. Primer sequences. Table S6. The miRNA names, primer sequences. (ZIP) [file pone.0060337.s001.zip › Supporting Information files/Table S2 Longan-known miRNA targets by predicition method.doc]

**Table S2** Longan-known miRNA targets by predicition method

| miRNA | Numbers | targets |
| --- | --- | --- |
| miR156 | 3 | Unigene9849_Longan-RNA[]~~Unigene62419_Longan-RNA[]~~Unigene67754_Longan-RNA[] |
| miR157 | 4 | Unigene21263_Longan-RNA[]~~Unigene41938_Longan-RNA[]~~Unigene55714_Longan-RNA[]~~Unigene67858_Longan-RNA[] |
| miR158 | 6 | Unigene2775_Longan-RNA[]~~Unigene3557_Longan-RNA[]~~Unigene46063_Longan-RNA[]~~Unigene64335_Longan-RNA[]~~Unigene66240_Longan-RNA[]~~Unigene68923_Longan-RNA[] |
| miR159 | 4 | Unigene8850_Longan-RNA[]~~Unigene43023_Longan-RNA[]~~Unigene66155_Longan-RNA[]~~Unigene2751_Longan-RNA[] |
| miR161 | 8 | Unigene7379_Longan-RNA[]~~Unigene43723_Longan-RNA[]~~Unigene51088_Longan-RNA[]~~Unigene54297_Longan-RNA[]~~Unigene66512_Longan-RNA[]~~Unigene67135_Longan-RNA[]~~Unigene67292_Longan-RNA[]~~Unigene68705_Longan-RNA[] |
| miR162 | 2 | Unigene56610_Longan-RNA[]~~Unigene67554_Longan-RNA[] |
| miR164 | 4 | Unigene49599_Longan-RNA[]~~Unigene59956_Longan-RNA[]~~Unigene61337_Longan-RNA[]~~Unigene67399_Longan-RNA[] |
| miR165 | 9 | Unigene10011_Longan-RNA[]~~Unigene12408_Longan-RNA[]~~Unigene14044_Longan-RNA[]~~Unigene15041_Longan-RNA[]~~Unigene18169_Longan-RNA[]~~Unigene61230_Longan-RNA[]~~Unigene61545_Longan-RNA[]~~Unigene62266_Longan-RNA[]~~Unigene66693_Longan-RNA[] |
| miR166 | 5 | Unigene1648_Longan-RNA[]~~Unigene2445_Longan-RNA[]~~Unigene12408_Longan-RNA[]~~Unigene14044_Longan-RNA[]~~Unigene67189_Longan-RNA[] |
| miR167 | 3 | Unigene62109_Longan-RNA[]~~Unigene62178_Longan-RNA[]~~Unigene65933_Longan-RNA[] |
| miR169 | 2 | Unigene13937_Longan-RNA[]~~Unigene51073_Longan-RNA[] |
| miR171 | 2 | Unigene371_Longan-RNA[]~~Unigene68246_Longan-RNA[] |
| miR172 | 4 | Unigene28760_Longan-RNA[]~~Unigene33814_Longan-RNA[]~~Unigene55590_Longan-RNA[]~~Unigene58780_Longan-RNA[] |
| miR319 | 4 | Unigene8850_Longan-RNA[]~~Unigene12714_Longan-RNA[]~~Unigene13760_Longan-RNA[]~~Unigene66155_Longan-RNA[] |
| miR390 | 5 | Unigene47434_Longan-RNA[]~~Unigene59323_Longan-RNA[]~~Unigene59838_Longan-RNA[]~~Unigene68460_Longan-RNA[]~~Unigene68460_Longan-RNA[] |
| miR393 | 2 | Unigene67838_Longan-RNA[]~~Unigene68515_Longan-RNA[] |
| miR394 | 4 | Unigene63526_Longan-RNA[]~~Unigene63687_Longan-RNA[]~~Unigene64668_Longan-RNA[]~~Unigene67240_Longan-RNA[] |
| miR396 | 1 | Unigene62142_Longan-RNA[] |
| miR397 | 8 | Unigene3015_Longan-RNA[]~~Unigene3137_Longan-RNA[]~~Unigene15055_Longan-RNA[]~~Unigene26626_Longan-RNA[]~~Unigene52662_Longan-RNA[]~~Unigene66334_Longan-RNA[]~~Unigene67636_Longan-RNA[]~~Unigene68183_Longan-RNA[] |
| miR400 | 10 | Unigene14756_Longan-RNA[]~~Unigene26909_Longan-RNA[]~~Unigene60348_Longan-RNA[]~~Unigene60949_Longan-RNA[]~~Unigene61550_Longan-RNA[]~~Unigene62595_Longan-RNA[]~~Unigene64133_Longan-RNA[]~~Unigene66597_Longan-RNA[]~~Unigene68759_Longan-RNA[]~~Unigene24279_Longan-RNA[] |
| miR403 | 2 | Unigene38235_Longan-RNA[]~~Unigene60084_Longan-RNA[] |
| miR406 | 1 | Unigene63438_Longan-RNA[] |
| miR408 | 2 | Unigene49825_Longan-RNA[]~~Unigene65686_Longan-RNA[] |
| miR413 | 1 | Unigene64813_Longan-RNA[] |
| miR418 | 4 | Unigene14024_Longan-RNA[]~~Unigene15260_Longan-RNA[]~~Unigene50116_Longan-RNA[]~~Unigene63792_Longan-RNA[] |
| miR420 | 5 | Unigene646_Longan-RNA[]~~Unigene13889_Longan-RNA[]~~Unigene46654_Longan-RNA[]~~Unigene62728_Longan-RNA[]~~Unigene37072_Longan-RNA[] |
| miR472 | 9 | Unigene315_Longan-RNA[]~~Unigene4977_Longan-RNA[]~~Unigene10592_Longan-RNA[]~~Unigene14207_Longan-RNA[]~~Unigene31200_Longan-RNA[]~~Unigene38673_Longan-RNA[]~~Unigene64103_Longan-RNA[]~~Unigene66192_Longan-RNA[]~~Unigene68540_Longan-RNA[] |
| miR473 | 16 | Unigene10372_Longan-RNA[]~~Unigene11847_Longan-RNA[]~~Unigene47326_Longan-RNA[]~~Unigene51348_Longan-RNA[]~~Unigene57913_Longan-RNA[]~~Unigene58435_Longan-RNA[]~~Unigene59219_Longan-RNA[]~~Unigene62228_Longan-RNA[]~~Unigene63278_Longan-RNA[]~~Unigene65290_Longan-RNA[]~~Unigene66230_Longan-RNA[]~~Unigene66372_Longan-RNA[]~~Unigene66820_Longan-RNA[]~~Unigene68764_Longan-RNA[]~~Unigene68866_Longan-RNA[]~~Unigene68892_Longan-RNA[] |
| miR476 | 16 | Unigene5250_Longan-RNA[]~~Unigene6831_Longan-RNA[]~~Unigene8799_Longan-RNA[]~~Unigene14150_Longan-RNA[]~~Unigene14199_Longan-RNA[]~~Unigene33669_Longan-RNA[]~~Unigene49694_Longan-RNA[]~~Unigene56690_Longan-RNA[]~~Unigene58489_Longan-RNA[]~~Unigene59143_Longan-RNA[]~~Unigene59311_Longan-RNA[]~~Unigene60033_Longan-RNA[]~~Unigene66302_Longan-RNA[]~~Unigene67198_Longan-RNA[]~~Unigene68004_Longan-RNA[]~~Unigene68337_Longan-RNA[] |
| miR477 | 8 | Unigene50052_Longan-RNA[]~~Unigene52324_Longan-RNA[]~~Unigene55353_Longan-RNA[]~~Unigene55935_Longan-RNA[]~~Unigene56008_Longan-RNA[]~~Unigene59832_Longan-RNA[]~~Unigene62688_Longan-RNA[]~~Unigene68771_Longan-RNA[] |
| miR482 | 5 | Unigene4977_Longan-RNA[]~~Unigene5428_Longan-RNA[]~~Unigene38673_Longan-RNA[]~~Unigene41724_Longan-RNA[]~~Unigene68540_Longan-RNA[] |
| miR528 | 6 | Unigene9336_Longan-RNA[]~~Unigene9461_Longan-RNA[]~~Unigene60491_Longan-RNA[]~~Unigene64344_Longan-RNA[]~~Unigene67749_Longan-RNA[]~~Unigene68243_Longan-RNA[] |
| miR536 | 7 | Unigene14962_Longan-RNA[]~~Unigene15207_Longan-RNA[]~~Unigene36448_Longan-RNA[]~~Unigene62979_Longan-RNA[]~~Unigene63354_Longan-RNA[]~~Unigene68234_Longan-RNA[]~~Unigene68544_Longan-RNA[] |
| miR773 | 1 | Unigene27416_Longan-RNA[] |
| miR778 | 6 | Unigene392_Longan-RNA[]~~Unigene9720_Longan-RNA[]~~Unigene9845_Longan-RNA[]~~Unigene13255_Longan-RNA[]~~Unigene59216_Longan-RNA[]~~Unigene64750_Longan-RNA[] |
| miR780 | 51 | Unigene5221_Longan-RNA[]~~Unigene5636_Longan-RNA[]~~Unigene5922_Longan-RNA[]~~Unigene6134_Longan-RNA[]~~Unigene7338_Longan-RNA[]~~Unigene7371_Longan-RNA[]~~Unigene7887_Longan-RNA[]~~Unigene8397_Longan-RNA[]~~Unigene8503_Longan-RNA[]~~Unigene9387_Longan-RNA[]~~Unigene11859_Longan-RNA[]~~Unigene12849_Longan-RNA[]~~Unigene13027_Longan-RNA[]~~Unigene14028_Longan-RNA[]~~Unigene15260_Longan-RNA[]~~Unigene21772_Longan-RNA[]~~Unigene26832_Longan-RNA[]~~Unigene27060_Longan-RNA[]~~Unigene28510_Longan-RNA[]~~Unigene33767_Longan-RNA[]~~Unigene34995_Longan-RNA[]~~Unigene36600_Longan-RNA[]~~Unigene36811_Longan-RNA[]~~Unigene39071_Longan-RNA[]~~Unigene44677_Longan-RNA[]~~Unigene46104_Longan-RNA[]~~Unigene47823_Longan-RNA[]~~Unigene51655_Longan-RNA[]~~Unigene51976_Longan-RNA[]~~Unigene53415_Longan-RNA[]~~Unigene54921_Longan-RNA[]~~Unigene55536_Longan-RNA[]~~Unigene58191_Longan-RNA[]~~Unigene58536_Longan-RNA[]~~Unigene58567_Longan-RNA[]~~Unigene59636_Longan-RNA[]~~Unigene61952_Longan-RNA[]~~Unigene62052_Longan-RNA[]~~Unigene62118_Longan-RNA[]~~Unigene62221_Longan-RNA[]~~Unigene63022_Longan-RNA[]~~Unigene64849_Longan-RNA[]~~Unigene65144_Longan-RNA[]~~Unigene65418_Longan-RNA[]~~Unigene66097_Longan-RNA[]~~Unigene67616_Longan-RNA[]~~Unigene68159_Longan-RNA[]~~Unigene68315_Longan-RNA[]~~Unigene68499_Longan-RNA[]~~Unigene68757_Longan-RNA[]~~Unigene68773_Longan-RNA[] |
| miR815 | 1 | Unigene13515_Longan-RNA[] |
| miR816 | 1 | Unigene9379_Longan-RNA[] |
| miR827 | 3 | Unigene8283_Longan-RNA[]~~Unigene10155_Longan-RNA[]~~Unigene61988_Longan-RNA[] |
| miR830 | 9 | Unigene14587_Longan-RNA[]~~Unigene19047_Longan-RNA[]~~Unigene30067_Longan-RNA[]~~Unigene55607_Longan-RNA[]~~Unigene62137_Longan-RNA[]~~Unigene64755_Longan-RNA[]~~Unigene67499_Longan-RNA[]~~Unigene67829_Longan-RNA[]~~Unigene68876_Longan-RNA[] |
| miR835 | 3 | Unigene10870_Longan-RNA[]~~Unigene24500_Longan-RNA[]~~Unigene31979_Longan-RNA[] |
| miR837 | 1 | Unigene11461_Longan-RNA[] |
| miR842 | 5 | Unigene10334_Longan-RNA[]~~Unigene14225_Longan-RNA[]~~Unigene15017_Longan-RNA[]~~Unigene45717_Longan-RNA[]~~Unigene50144_Longan-RNA[] |
| miR843 | 4 | Unigene54090_Longan-RNA[]~~Unigene64067_Longan-RNA[]~~Unigene64507_Longan-RNA[]~~Unigene68179_Longan-RNA[] |
| miR854 | 118 | Unigene743_Longan-RNA[]~~Unigene766_Longan-RNA[]~~Unigene1229_Longan-RNA[]~~Unigene2788_Longan-RNA[]~~Unigene2974_Longan-RNA[]~~Unigene3111_Longan-RNA[]~~Unigene4169_Longan-RNA[]~~Unigene5140_Longan-RNA[]~~Unigene5396_Longan-RNA[]~~Unigene5396_Longan-RNA[]~~Unigene5396_Longan-RNA[]~~Unigene5396_Longan-RNA[]~~Unigene5441_Longan-RNA[]~~Unigene6315_Longan-RNA[]~~Unigene7819_Longan-RNA[]~~Unigene7991_Longan-RNA[]~~Unigene9282_Longan-RNA[]~~Unigene9290_Longan-RNA[]~~Unigene9709_Longan-RNA[]~~Unigene10309_Longan-RNA[]~~Unigene10309_Longan-RNA[]~~Unigene10543_Longan-RNA[]~~Unigene10936_Longan-RNA[]~~Unigene11129_Longan-RNA[]~~Unigene11191_Longan-RNA[]~~Unigene11415_Longan-RNA[]~~Unigene11500_Longan-RNA[]~~Unigene11517_Longan-RNA[]~~Unigene11631_Longan-RNA[]~~Unigene12335_Longan-RNA[]~~Unigene12356_Longan-RNA[]~~Unigene12714_Longan-RNA[]~~Unigene12918_Longan-RNA[]~~Unigene13534_Longan-RNA[]~~Unigene13882_Longan-RNA[]~~Unigene13984_Longan-RNA[]~~Unigene14219_Longan-RNA[]~~Unigene14773_Longan-RNA[]~~Unigene17226_Longan-RNA[]~~Unigene17384_Longan-RNA[]~~Unigene19647_Longan-RNA[]~~Unigene21323_Longan-RNA[]~~Unigene24637_Longan-RNA[]~~Unigene28714_Longan-RNA[]~~Unigene32764_Longan-RNA[]~~Unigene34176_Longan-RNA[]~~Unigene34370_Longan-RNA[]~~Unigene37438_Longan-RNA[]~~Unigene39935_Longan-RNA[]~~Unigene41945_Longan-RNA[]~~Unigene43181_Longan-RNA[]~~Unigene43385_Longan-RNA[]~~Unigene44027_Longan-RNA[]~~Unigene44036_Longan-RNA[]~~Unigene45314_Longan-RNA[]~~Unigene45314_Longan-RNA[]~~Unigene46189_Longan-RNA[]~~Unigene47363_Longan-RNA[]~~Unigene47434_Longan-RNA[]~~Unigene48887_Longan-RNA[]~~Unigene49058_Longan-RNA[]~~Unigene49058_Longan-RNA[]~~Unigene50924_Longan-RNA[]~~Unigene51529_Longan-RNA[]~~Unigene52373_Longan-RNA[]~~Unigene52421_Longan-RNA[]~~Unigene54551_Longan-RNA[]~~Unigene54773_Longan-RNA[]~~Unigene54984_Longan-RNA[]~~Unigene55302_Longan-RNA[]~~Unigene55415_Longan-RNA[]~~Unigene55639_Longan-RNA[]~~Unigene55896_Longan-RNA[]~~Unigene56025_Longan-RNA[]~~Unigene56316_Longan-RNA[]~~Unigene56548_Longan-RNA[]~~Unigene56760_Longan-RNA[]~~Unigene57046_Longan-RNA[]~~Unigene57121_Longan-RNA[]~~Unigene59380_Longan-RNA[]~~Unigene59390_Longan-RNA[]~~Unigen59472_Longan-RNA[]~~Unigene59634_Longan-RNA[]~~Unigene59676_Longan-RNA[]~~Unigene60021_Longan-RNA[]~~Unigene60037_Longan-RNA[]~~Unigene60546_Longan-RNA[]~~Unigene61195_Longan-RNA[]~~Unigene61326_Longan-RNA[]~~Unigene61857_Longan-RNA[]~~Unigene62027_Longan-RNA[]~~Unigene63095_Longan-RNA[]~~Unigene64116_Longan-RNA[]~~Unigene64439_Longan-RNA[]~~Unigene64856_Longan-RNA[]~~Unigene64941_Longan-RNA[]~~Unigene65097_Longan-RNA[]~~Unigene65649_Longan-RNA[]~~Unigene66040_Longan-RNA[]~~Unigene66156_Longan-RNA[]~~Unigene66873_Longan-RNA[]~~Unigene67021_Longan-RNA[]~~Unigene67073_Longan-RNA[]~~Unigene67567_Longan-RNA[]~~Unigene67576_Longan-RNA[]~~Unigene67772_Longan-RNA[]~~Unigene67922_Longan-RNA[]~~Unigene68062_Longan-RNA[]~~Unigene68337_Longan-RNA[]~~Unigene68349_Longan-RNA[]~~Unigene68382_Longan-RNA[]~~Unigene68382_Longan-RNA[]~~Unigene68437_Longan-RNA[]~~Unigene68647_Longan-RNA[]~~Unigene68690_Longan-RNA[]~~Unigene68746_Longan-RNA[]~~Unigene68817_Longan-RNA[]~~Unigene52132_Longan-RNA[] |
| miR857 | 1 | Unigene64767_Longan-RNA[] |
| miR858 | 3 | Unigene49867_Longan-RNA[]~~Unigene61908_Longan-RNA[]~~Unigene65909_Longan-RNA[] |
| miR860 | 7 | Unigene2132_Longan-RNA[]~~Unigene11374_Longan-RNA[]~~Unigene62865_Longan-RNA[]~~Unigene65821_Longan-RNA[]~~Unigene67257_Longan-RNA[]~~Unigene67744_Longan-RNA[]~~Unigene68740_Longan-RNA[] |
| miR861 | 3 | Unigene6986_Longan-RNA[]~~Unigene43929_Longan-RNA[]~~Unigene66576_Longan-RNA[] |
| miR862 | 6 | Unigene12924_Longan-RNA[]~~Unigene16915_Longan-RNA[]~~Unigene29313_Longan-RNA[]~~Unigene47830_Longan-RNA[]~~Unigene52622_Longan-RNA[]~~Unigene56563_Longan-RNA[] |
| miR866 | 20 | Unigene7119_Longan-RNA[]~~Unigene25954_Longan-RNA[]~~Unigene33998_Longan-RNA[]~~Unigene42854_Longan-RNA[]~~Unigene60791_Longan-RNA[]~~Unigene61011_Longan-RNA[]~~Unigene61817_Longan-RNA[]~~Unigene64340_Longan-RNA[]~~Unigene65027_Longan-RNA[]~~Unigene65439_Longan-RNA[]~~Unigene65772_Longan-RNA[]~~Unigene65782_Longan-RNA[]~~Unigene67023_Longan-RNA[]~~Unigene67400_Longan-RNA[]~~Unigene67469_Longan-RNA[]~~Unigene67790_Longan-RNA[]~~Unigene67953_Longan-RNA[]~~Unigene68235_Longan-RNA[]~~Unigene68236_Longan-RNA[]~~Unigene62726_Longan-RNA[] |
| miR869 | 3 | Unigene53291_Longan-RNA[]~~Unigene64500_Longan-RNA[]~~Unigene65825_Longan-RNA[] |
| miR870 | 38 | Unigene837_Longan-RNA[]~~Unigene1740_Longan-RNA[]~~Unigene3261_Longan-RNA[]~~Unigene6823_Longan-RNA[]~~Unigene8664_Longan-RNA[]~~Unigene8915_Longan-RNA[]~~Unigene13472_Longan-RNA[]~~Unigene14181_Longan-RNA[]~~Unigene30341_Longan-RNA[]~~Unigene36489_Longan-RNA[]~~Unigene45473_Longan-RNA[]~~Unigene47733_Longan-RNA[]~~Unigene48336_Longan-RNA[]~~Unigene49090_Longan-RNA[]~~Unigene50749_Longan-RNA[]~~Unigene51433_Longan-RNA[]~~Unigene51546_Longan-RNA[]~~Unigene51664_Longan-RNA[]~~Unigene55174_Longan-RNA[]~~Unigene57409_Longan-RNA[]~~Unigene58061_Longan-RNA[]~~Unigene58692_Longan-RNA[]~~Unigene59153_Longan-RNA[]~~Unigene60351_Longan-RNA[]~~Unigene61165_Longan-RNA[]~~Unigene62315_Longan-RNA[]~~Unigene63515_Longan-RNA[]~~Unigene63834_Longan-RNA[]~~Unigene65729_Longan-RNA[]~~Unigene66528_Longan-RNA[]~~Unigene67128_Longan-RNA[]~~Unigene67147_Longan-RNA[]~~Unigene67663_Longan-RNA[]~~Unigene68072_Longan-RNA[]~~Unigene68176_Longan-RNA[]~~Unigene68723_Longan-RNA[]~~Unigene68922_Longan-RNA[]~~Unigene41740_Longan-RNA[] |
| miR898 | 1 | Unigene67508_Longan-RNA[] |
| miR900 | 1 | Unigene63919_Longan-RNA[] |
| miR908 | 2 | Unigene58489_Longan-RNA[]~~Unigene68032_Longan-RNA[] |
| miR913 | 1 | Unigene54885_Longan-RNA[] |
| miR916 | 13 | Unigene634_Longan-RNA[]~~Unigene5763_Longan-RNA[]~~Unigene6002_Longan-RNA[]~~Unigene10960_Longan-RNA[]~~Unigene11859_Longan-RNA[]~~Unigene12104_Longan-RNA[]~~Unigene13059_Longan-RNA[]~~Unigene46921_Longan-RNA[]~~Unigene48548_Longan-RNA[]~~Unigene62867_Longan-RNA[]~~Unigene65813_Longan-RNA[]~~Unigene66166_Longan-RNA[]~~Unigene66511_Longan-RNA[] |
| miR918 | 1 | Unigene59449_Longan-RNA[] |
| miR948 | 1 | Unigene62305_Longan-RNA[] |
| miR951 | 1 | Unigene51351_Longan-RNA[] |
| miR952 | 23 | Unigene6094_Longan-RNA[]~~Unigene11979_Longan-RNA[]~~Unigene14684_Longan-RNA[]~~Unigene14848_Longan-RNA[]~~Unigene15248_Longan-RNA[]~~Unigene15248_Longan-RNA[]~~Unigene18089_Longan-RNA[]~~Unigene35479_Longan-RNA[]~~Unigene50027_Longan-RNA[]~~Unigene53301_Longan-RNA[]~~Unigene54718_Longan-RNA[]~~Unigene55251_Longan-RNA[]~~Unigene57302_Longan-RNA[]~~Unigene63234_Longan-RNA[]~~Unigene65133_Longan-RNA[]~~Unigene66408_Longan-RNA[]~~Unigene66877_Longan-RNA[]~~Unigene67591_Longan-RNA[]~~Unigene68246_Longan-RNA[]~~Unigene68788_Longan-RNA[]~~Unigene68894_Longan-RNA[]~~Unigene68906_Longan-RNA[]~~Unigene46526_Longan-RNA[] |
| miR1024 | 5 | Unigene12696_Longan-RNA[]~~Unigene32962_Longan-RNA[]~~Unigene40661_Longan-RNA[]~~Unigene66622_Longan-RNA[]~~Unigene13708_Longan-RNA[] |
| miR1027 | 27 | Unigene695_Longan-RNA[]~~Unigene6385_Longan-RNA[]~~Unigene6385_Longan-RNA[]~~Unigene6385_Longan-RNA[]~~Unigene9160_Longan-RNA[]~~Unigene9248_Longan-RNA[]~~Unigene17487_Longan-RNA[]~~Unigene18621_Longan-RNA[]~~Unigene22391_Longan-RNA[]~~Unigene22505_Longan-RNA[]~~Unigene24554_Longan-RNA[]~~Unigene31583_Longan-RNA[]~~Unigene46547_Longan-RNA[]~~Unigene47279_Longan-RNA[]~~Unigene54584_Longan-RNA[]~~Unigene59468_Longan-RNA[]~~Unigene60100_Longan-RNA[]~~Unigene61194_Longan-RNA[]~~Unigene64337_Longan-RNA[]~~Unigene65812_Longan-RNA[]~~Unigene66352_Longan-RNA[]~~Unigene67471_Longan-RNA[]~~Unigene67637_Longan-RNA[]~~Unigene68020_Longan-RNA[]~~Unigene68496_Longan-RNA[]~~Unigene19134_Longan-RNA[]~~Unigene46877_Longan-RNA[] |
| miR1040 | 1 | Unigene10871_Longan-RNA[] |
| miR1041 | 2 | Unigene5772_Longan-RNA[]~~Unigene62738_Longan-RNA[] |
| miR1044 | 2 | Unigene50991_Longan-RNA[]~~Unigene64814_Longan-RNA[] |
| miR1046 | 42 | Unigene241_Longan-RNA[]~~Unigene1123_Longan-RNA[]~~Unigene2123_Longan-RNA[]~~Unigene3394_Longan-RNA[]~~Unigene3697_Longan-RNA[]~~Unigene5440_Longan-RNA[]~~Unigene5896_Longan-RNA[]~~Unigene7721_Longan-RNA[]~~Unigene7821_Longan-RNA[]~~Unigene8119_Longan-RNA[]~~Unigene9599_Longan-RNA[]~~Unigene10760_Longan-RNA[]~~Unigene11056_Longan-RNA[]~~Unigene12392_Longan-RNA[]~~Unigene14735_Longan-RNA[]~~Unigene15158_Longan-RNA[]~~Unigene24820_Longan-RNA[]~~Unigene25204_Longan-RNA[]~~Unigene41133_Longan-RNA[]~~Unigene46713_Longan-RNA[]~~Unigene50541_Longan-RNA[]~~Unigene50966_Longan-RNA[]~~Unigene54909_Longan-RNA[]~~Unigene56387_Longan-RNA[]~~Unigene56559_Longan-RNA[]~~Unigene57437_Longan-RNA[]~~Unigene58816_Longan-RNA[]~~Unigene58858_Longan-RNA[]~~Unigene61357_Longan-RNA[]~~Unigene62047_Longan-RNA[]~~Unigene62276_Longan-RNA[]~~Unigene62659_Longan-RNA[]~~Unigene63358_Longan-RNA[]~~Unigene64144_Longan-RNA[]~~Unigene64483_Longan-RNA[]~~Unigene66377_Longan-RNA[]~~Unigene66905_Longan-RNA[]~~Unigene67172_Longan-RNA[]~~Unigene67438_Longan-RNA[]~~Unigene68193_Longan-RNA[]~~Unigene68808_Longan-RNA[]~~Unigene2958_Longan-RNA[] |
| miR1058 | 5 | Unigene55994_Longan-RNA[]~~Unigene66848_Longan-RNA[]~~Unigene67809_Longan-RNA[]~~Unigene68568_Longan-RNA[]~~Unigene68846_Longan-RNA[] |
| miR1061 | 7 | Unigene10828_Longan-RNA[]~~Unigene12979_Longan-RNA[]~~Unigene42058_Longan-RNA[]~~Unigene62032_Longan-RNA[]~~Unigene68094_Longan-RNA[]~~Unigene68308_Longan-RNA[]~~Unigene8552_Longan-RNA[] |
| miR1064 | 1 | Unigene12175_Longan-RNA[] |
| miR1067 | 7 | Unigene5913_Longan-RNA[]~~Unigene13276_Longan-RNA[]~~Unigene41967_Longan-RNA[]~~Unigene48735_Longan-RNA[]~~Unigene62900_Longan-RNA[]~~Unigene67552_Longan-RNA[]~~Unigene68070_Longan-RNA[] |
| miR1069 | 4 | Unigene1674_Longan-RNA[]~~Unigene3532_Longan-RNA[]~~Unigene12678_Longan-RNA[]~~Unigene68721_Longan-RNA[] |
| miR1074 | 6 | Unigene23589_Longan-RNA[]~~Unigene41253_Longan-RNA[]~~Unigene59495_Longan-RNA[]~~Unigene60420_Longan-RNA[]~~Unigene63112_Longan-RNA[]~~Unigene65529_Longan-RNA[] |
| miR1081 | 12 | Unigene8084_Longan-RNA[]~~Unigene15098_Longan-RNA[]~~Unigene23264_Longan-RNA[]~~Unigene33802_Longan-RNA[]~~Unigene45227_Longan-RNA[]~~Unigene50757_Longan-RNA[]~~Unigene55714_Longan-RNA[]~~Unigene63220_Longan-RNA[]~~Unigene66793_Longan-RNA[]~~Unigene66879_Longan-RNA[]~~Unigene68246_Longan-RNA[]~~Unigene68478_Longan-RNA[] |
| miR1098 | 1 | Unigene48163_Longan-RNA[] |
| miR1102 | 10 | Unigene7887_Longan-RNA[]~~Unigene8305_Longan-RNA[]~~Unigene13183_Longan-RNA[]~~Unigene33956_Longan-RNA[]~~Unigene50198_Longan-RNA[]~~Unigene58645_Longan-RNA[]~~Unigene65823_Longan-RNA[]~~Unigene65936_Longan-RNA[]~~Unigene67820_Longan-RNA[]~~Unigene57613_Longan-RNA[] |
| miR1105 | 1 | Unigene14501_Longan-RNA[] |
| miR1108 | 1 | Unigene65314_Longan-RNA[] |
| miR1114 | 4 | Unigene5783_Longan-RNA[]~~Unigene7170_Longan-RNA[]~~Unigene14989_Longan-RNA[]~~Unigene49033_Longan-RNA[] |
| miR1122 | 5 | Unigene14184_Longan-RNA[]~~Unigene14274_Longan-RNA[]~~Unigene27365_Longan-RNA[]~~Unigene45355_Longan-RNA[]~~Unigene67175_Longan-RNA[] |
| miR1127 | 7 | Unigene8292_Longan-RNA[]~~Unigene12785_Longan-RNA[]~~Unigene12858_Longan-RNA[]~~Unigene54532_Longan-RNA[]~~Unigene59033_Longan-RNA[]~~Unigene63807_Longan-RNA[]~~Unigene66050_Longan-RNA[] |
| miR1132 | 5 | Unigene8659_Longan-RNA[]~~Unigene40306_Longan-RNA[]~~Unigene45523_Longan-RNA[]~~Unigene65009_Longan-RNA[]~~Unigene65165_Longan-RNA[] |
| miR1137 | 18 | Unigene6901_Longan-RNA[]~~Unigene8415_Longan-RNA[]~~Unigene11860_Longan-RNA[]~~Unigene12746_Longan-RNA[]~~Unigene14638_Longan-RNA[]~~Unigene15199_Longan-RNA[]~~Unigene27936_Longan-RNA[]~~Unigene31073_Longan-RNA[]~~Unigene33126_Longan-RNA[]~~Unigene48734_Longan-RNA[]~~Unigene55003_Longan-RNA[]~~Unigene59694_Longan-RNA[]~~Unigene65162_Longan-RNA[]~~Unigene65233_Longan-RNA[]~~Unigene65239_Longan-RNA[]~~Unigene67856_Longan-RNA[]~~Unigene68922_Longan-RNA[]~~Unigene29745_Longan-RNA[] |
| miR1139 | 7 | Unigene6327_Longan-RNA[]~~Unigene11552_Longan-RNA[]~~Unigene12097_Longan-RNA[]~~Unigene62622_Longan-RNA[]~~Unigene66511_Longan-RNA[]~~Unigene67170_Longan-RNA[]~~Unigene68371_Longan-RNA[] |
| miR1146 | 2 | Unigene13949_Longan-RNA[]~~Unigene60084_Longan-RNA[] |
| miR1147 | 3 | Unigene14918_Longan-RNA[]~~Unigene46416_Longan-RNA[]~~Unigene49088_Longan-RNA[] |
| miR1150 | 3 | Unigene15009_Longan-RNA[]~~Unigene16079_Longan-RNA[]~~Unigene55859_Longan-RNA[] |
| miR1153 | 1 | Unigene15031_Longan-RNA[] |
| miR1154 | 2 | Unigene5160_Longan-RNA[]~~Unigene36724_Longan-RNA[] |
| miR1169 | 5 | Unigene11181_Longan-RNA[]~~Unigene14704_Longan-RNA[]~~Unigene50451_Longan-RNA[]~~Unigene56281_Longan-RNA[]~~Unigene67068_Longan-RNA[] |
| miR1170 | 11 | Unigene9366_Longan-RNA[]~~Unigene12242_Longan-RNA[]~~Unigene22095_Longan-RNA[]~~Unigene22869_Longan-RNA[]~~Unigene53962_Longan-RNA[]~~Unigene54036_Longan-RNA[]~~Unigene60538_Longan-RNA[]~~Unigene60653_Longan-RNA[]~~Unigene61129_Longan-RNA[]~~Unigene62355_Longan-RNA[]~~Unigene65423_Longan-RNA[] |
| miR1211 | 24 | Unigene7819_Longan-RNA[]~~Unigene8320_Longan-RNA[]~~Unigene12642_Longan-RNA[]~~Unigene17226_Longan-RNA[]~~Unigene25733_Longan-RNA[]~~Unigene30773_Longan-RNA[]~~Unigene35326_Longan-RNA[]~~Unigene43385_Longan-RNA[]~~Unigene46575_Longan-RNA[]~~Unigene47434_Longan-RNA[]~~Unigene47557_Longan-RNA[]~~Unigene47832_Longan-RNA[]~~Unigene50686_Longan-RNA[]~~Unigene51501_Longan-RNA[]~~Unigene52315_Longan-RNA[]~~Unigene54147_Longan-RNA[]~~Unigene60279_Longan-RNA[]~~Unigene62027_Longan-RNA[]~~Unigene64294_Longan-RNA[]~~Unigene65904_Longan-RNA[]~~Unigene67816_Longan-RNA[]~~Unigene67841_Longan-RNA[]~~Unigene68062_Longan-RNA[]~~Unigene68720_Longan-RNA[] |
| miR1217 | 6 | Unigene7974_Longan-RNA[]~~Unigene12498_Longan-RNA[]~~Unigene52609_Longan-RNA[]~~Unigene65171_Longan-RNA[]~~Unigene66435_Longan-RNA[]~~Unigene67915_Longan-RNA[] |
| miR1223 | 8 | Unigene1146_Longan-RNA[]~~Unigene12598_Longan-RNA[]~~Unigene13811_Longan-RNA[]~~Unigene14922_Longan-RNA[]~~Unigene14992_Longan-RNA[]~~Unigene57619_Longan-RNA[]~~Unigene65565_Longan-RNA[]~~Unigene66739_Longan-RNA[] |
| miR1313 | 2 | Unigene13865_Longan-RNA[]~~Unigene43457_Longan-RNA[] |
| miR1316 | 11 | Unigene8329_Longan-RNA[]~~Unigene11139_Longan-RNA[]~~Unigene14311_Longan-RNA[]~~Unigene15037_Longan-RNA[]~~Unigene48401_Longan-RNA[]~~Unigene56927_Longan-RNA[]~~Unigene59655_Longan-RNA[]~~Unigene59843_Longan-RNA[]~~Unigene62085_Longan-RNA[]~~Unigene66898_Longan-RNA[]~~Unigene67924_Longan-RNA[] |
| miR1317 | 1 | Unigene60504_Longan-RNA[] |
| miR1318 | 7 | Unigene695_Longan-RNA[]~~Unigene9587_Longan-RNA[]~~Unigene15083_Longan-RNA[]~~Unigene63381_Longan-RNA[]~~Unigene64107_Longan-RNA[]~~Unigene64485_Longan-RNA[]~~Unigene68138_Longan-RNA[] |
| miR1320 | 12 | Unigene4052_Longan-RNA[]~~Unigene5149_Longan-RNA[]~~Unigene12564_Longan-RNA[]~~Unigene15055_Longan-RNA[]~~Unigene15209_Longan-RNA[]~~Unigene28971_Longan-RNA[]~~Unigene30783_Longan-RNA[]~~Unigene48818_Longan-RNA[]~~Unigene56760_Longan-RNA[]~~Unigene65431_Longan-RNA[]~~Unigene65560_Longan-RNA[]~~Unigene68058_Longan-RNA[] |
| miR1424 | 7 | Unigene7862_Longan-RNA[]~~Unigene14921_Longan-RNA[]~~Unigene39304_Longan-RNA[]~~Unigene53967_Longan-RNA[]~~Unigene64231_Longan-RNA[]~~Unigene64509_Longan-RNA[]~~Unigene16375_Longan-RNA[] |
| miR1426 | 35 | Unigene3662_Longan-RNA[]~~Unigene3865_Longan-RNA[]~~Unigene5250_Longan-RNA[]~~Unigene6218_Longan-RNA[]~~Unigene7379_Longan-RNA[]~~Unigene8945_Longan-RNA[]~~Unigene10587_Longan-RNA[]~~Unigene11601_Longan-RNA[]~~Unigene12639_Longan-RNA[]~~Unigene13912_Longan-RNA[]~~Unigene14144_Longan-RNA[]~~Unigene14517_Longan-RNA[]~~Unigene24730_Longan-RNA[]~~Unigene25987_Longan-RNA[]~~Unigene41212_Longan-RNA[]~~Unigene46875_Longan-RNA[]~~Unigene48436_Longan-RNA[]~~Unigene49756_Longan-RNA[]~~Unigene51508_Longan-RNA[]~~Unigene53255_Longan-RNA[]~~Unigene55192_Longan-RNA[]~~Unigene58692_Longan-RNA[]~~Unigene63798_Longan-RNA[]~~Unigene63834_Longan-RNA[]~~Unigene64463_Longan-RNA[]~~Unigene64663_Longan-RNA[]~~Unigene66519_Longan-RNA[]~~Unigene67601_Longan-RNA[]~~Unigene67893_Longan-RNA[]~~Unigene68209_Longan-RNA[]~~Unigene68471_Longan-RNA[]~~Unigene68548_Longan-RNA[]~~Unigene68621_Longan-RNA[]~~Unigene68698_Longan-RNA[]~~Unigene68748_Longan-RNA[] |
| miR1429 | 3 | Unigene41032_Longan-RNA[]~~Unigene56571_Longan-RNA[]~~Unigene61358_Longan-RNA[] |
| miR1431 | 2 | Unigene67781_Longan-RNA[]~~Unigene68322_Longan-RNA[] |
| miR1436 | 3 | Unigene5226_Longan-RNA[]~~Unigene10293_Longan-RNA[]~~Unigene66909_Longan-RNA[] |
| miR1438 | 9 | Unigene1014_Longan-RNA[]~~Unigene6342_Longan-RNA[]~~Unigene10312_Longan-RNA[]~~Unigene19410_Longan-RNA[]~~Unigene20567_Longan-RNA[]~~Unigene61899_Longan-RNA[]~~Unigene64584_Longan-RNA[]~~Unigene66995_Longan-RNA[]~~Unigene68757_Longan-RNA[] |
| miR1442 | 48 | Unigene715_Longan-RNA[]~~Unigene953_Longan-RNA[]~~Unigene1353_Longan-RNA[]~~Unigene1421_Longan-RNA[]~~Unigene2771_Longan-RNA[]~~Unigene2911_Longan-RNA[]~~Unigene3846_Longan-RNA[]~~Unigene10263_Longan-RNA[]~~Unigene11072_Longan-RNA[]~~Unigene13779_Longan-RNA[]~~Unigene13805_Longan-RNA[]~~Unigene14063_Longan-RNA[]~~Unigene14776_Longan-RNA[]~~Unigene14872_Longan-RNA[]~~Unigene15109_Longan-RNA[]~~Unigene15248_Longan-RNA[]~~Unigene15489_Longan-RNA[]~~Unigene40727_Longan-RNA[]~~Unigene44695_Longan-RNA[]~~Unigene49492_Longan-RNA[]~~Unigene51514_Longan-RNA[]~~Unigene55037_Longan-RNA[]~~Unigene55115_Longan-RNA[]~~Unigene55961_Longan-RNA[]~~Unigene58816_Longan-RNA[]~~Unigene58882_Longan-RNA[]~~Unigene59891_Longan-RNA[]~~Unigene60967_Longan-RNA[]~~Unigene63091_Longan-RNA[]~~Unigene63842_Longan-RNA[]~~Unigene64069_Longan-RNA[]~~Unigene64091_Longan-RNA[]~~Unigene64163_Longan-RNA[]~~Unigene64285_Longan-RNA[]~~Unigene64328_Longan-RNA[]~~Unigene64583_Longan-RNA[]~Unigene64773_Longan-RNA[]~~Unigene67186_Longan-RNA[]~~Unigene67359_Longan-RNA[]~~Unigene67477_Longan-RNA[]~~Unigene67797_Longan-RNA[]~~Unigene68013_Longan-RNA[]~~Unigene68022_Longan-RNA[]~~Unigene68111_Longan-RNA[]~~Unigene68287_Longan-RNA[]~~Unigene68623_Longan-RNA[]~~Unigene68849_Longan-RNA[]~~Unigene56889_Longan-RNA[] |
| miR1444 | 1 | Unigene14907_Longan-RNA[] |
| miR1448 | 10 | Unigene1651_Longan-RNA[]~~Unigene1879_Longan-RNA[]~~Unigene12828_Longan-RNA[]~~Unigene30192_Longan-RNA[]~~Unigene52285_Longan-RNA[]~~Unigene64357_Longan-RNA[]~~Unigene65265_Longan-RNA[]~~Unigene67878_Longan-RNA[]~~Unigene68886_Longan-RNA[]~~Unigene18453_Longan-RNA[] |
| miR1507 | 1 | Unigene14484_Longan-RNA[] |
| miR1508 | 12 | Unigene1507_Longan-RNA[]~~Unigene2012_Longan-RNA[]~~Unigene10818_Longan-RNA[]~~Unigene12996_Longan-RNA[]~~Unigene13839_Longan-RNA[]~~Unigene14301_Longan-RNA[]~~Unigene39717_Longan-RNA[]~~Unigene55113_Longan-RNA[]~~Unigene65450_Longan-RNA[]~~Unigene66956_Longan-RNA[]~~Unigene68357_Longan-RNA[]~~Unigene53748_Longan-RNA[] |
| miR1510 | 5 | Unigene11977_Longan-RNA[]~~Unigene14520_Longan-RNA[]~~Unigene66297_Longan-RNA[]~~Unigene66459_Longan-RNA[]~~Unigene68636_Longan-RNA[] |
| miR1512 | 14 | Unigene2355_Longan-RNA[]~~Unigene3881_Longan-RNA[]~~Unigene27008_Longan-RNA[]~~Unigene39798_Longan-RNA[]~~Unigene48176_Longan-RNA[]~~Unigene48858_Longan-RNA[]~~Unigene49588_Longan-RNA[]~~Unigene56476_Longan-RNA[]~~Unigene56556_Longan-RNA[]~~Unigene61517_Longan-RNA[]~~Unigene62631_Longan-RNA[]~~Unigene64078_Longan-RNA[]~~Unigene66022_Longan-RNA[]~~Unigene22233_Longan-RNA[] |
| miR1513 | 5 | Unigene6858_Longan-RNA[]~~Unigene9689_Longan-RNA[]~~Unigene30420_Longan-RNA[]~~Unigene54850_Longan-RNA[]~~Unigene66274_Longan-RNA[] |
| miR1517 | 1 | Unigene11056_Longan-RNA[] |
| miR1519 | 33 | Unigene95_Longan-RNA[]~~Unigene2141_Longan-RNA[]~~Unigene2685_Longan-RNA[]~~Unigene7992_Longan-RNA[]~~Unigene13031_Longan-RNA[]~~Unigene15080_Longan-RNA[]~~Unigene23692_Longan-RNA[]~~Unigene32555_Longan-RNA[]~~Unigene33634_Longan-RNA[]~~Unigene40913_Longan-RNA[]~~Unigene41004_Longan-RNA[]~~Unigene44714_Longan-RNA[]~~Unigene52695_Longan-RNA[]~~Unigene55891_Longan-RNA[]~~Unigene56098_Longan-RNA[]~~Unigene56924_Longan-RNA[]~~Unigene57983_Longan-RNA[]~~Unigene60459_Longan-RNA[]~~Unigene61289_Longan-RNA[]~~Unigene64561_Longan-RNA[]~~Unigene65580_Longan-RNA[]~~Unigene65810_Longan-RNA[]~~Unigene66482_Longan-RNA[]~~Unigene66544_Longan-RNA[]~~Unigene67483_Longan-RNA[]~~Unigene67801_Longan-RNA[]~~Unigene68342_Longan-RNA[]~~Unigene68474_Longan-RNA[]~~Unigene68585_Longan-RNA[]~~Unigene68692_Longan-RNA[]~~Unigene68763_Longan-RNA[]~~Unigene68821_Longan-RNA[]~~Unigene68883_Longan-RNA[] |
| miR1520 | 2 | Unigene62142_Longan-RNA[]~~Unigene43087_Longan-RNA[] |
| miR1521 | 8 | Unigene580_Longan-RNA[]~~Unigene4032_Longan-RNA[]~~Unigene10910_Longan-RNA[]~~Unigene54804_Longan-RNA[]~~Unigene60633_Longan-RNA[]~~Unigene66435_Longan-RNA[]~~Unigene67599_Longan-RNA[]~~Unigene67849_Longan-RNA[] |
| miR1522 | 1 | Unigene11769_Longan-RNA[] |
| miR1527 | 2 | Unigene11441_Longan-RNA[]~~Unigene47371_Longan-RNA[] |
| miR1533 | 6 | Unigene2293_Longan-RNA[]~~Unigene12255_Longan-RNA[]~~Unigene24355_Longan-RNA[]~~Unigene26964_Longan-RNA[]~~Unigene39522_Longan-RNA[]~~Unigene65089_Longan-RNA[] |
| miR1535 | 11 | Unigene5924_Longan-RNA[]~~Unigene10904_Longan-RNA[]~~Unigene41479_Longan-RNA[]~~Unigene58714_Longan-RNA[]~~Unigene59801_Longan-RNA[]~~Unigene61048_Longan-RNA[]~~Unigene63476_Longan-RNA[]~~Unigene66096_Longan-RNA[]~~Unigene68401_Longan-RNA[]~~Unigene68817_Longan-RNA[]~~Unigene36703_Longan-RNA[] |
| miR1846 | 3 | Unigene26363_Longan-RNA[]~~Unigene46052_Longan-RNA[]~~Unigene68713_Longan-RNA[] |
| miR1847 | 6 | Unigene11860_Longan-RNA[]~~Unigene13614_Longan-RNA[]~~Unigene26605_Longan-RNA[]~~Unigene56642_Longan-RNA[]~~Unigene68465_Longan-RNA[]~~Unigene68916_Longan-RNA[] |
| miR1849 | 4 | Unigene2609_Longan-RNA[]~~Unigene57942_Longan-RNA[]~~Unigene68602_Longan-RNA[]~~Unigene68884_Longan-RNA[] |
| miR1854 | 2 | Unigene61477_Longan-RNA[]~~Unigene63457_Longan-RNA[] |
| miR1856 | 11 | Unigene3557_Longan-RNA[]~~Unigene11700_Longan-RNA[]~~Unigene12907_Longan-RNA[]~~Unigene14045_Longan-RNA[]~~Unigene36604_Longan-RNA[]~~Unigene55149_Longan-RNA[]~~Unigene56144_Longan-RNA[]~~Unigene57247_Longan-RNA[]~~Unigene62949_Longan-RNA[]~~Unigene66401_Longan-RNA[]~~Unigene56506_Longan-RNA[] |
| miR1860 | 2 | Unigene4132_Longan-RNA[]~~Unigene53674_Longan-RNA[] |
| miR1886 | 1 | Unigene68492_Longan-RNA[] |
| miR1887 | 2 | Unigene4162_Longan-RNA[]~~Unigene14271_Longan-RNA[] |
| miR1917 | 18 | Unigene10_Longan-RNA[]~~Unigene8792_Longan-RNA[]~~Unigene12498_Longan-RNA[]~~Unigene12640_Longan-RNA[]~~Unigene12888_Longan-RNA[]~~Unigene13036_Longan-RNA[]~~Unigene15009_Longan-RNA[]~~Unigene25194_Longan-RNA[]~~Unigene48145_Longan-RNA[]~~Unigene59713_Longan-RNA[]~~Unigene61185_Longan-RNA[]~~Unigene61213_Longan-RNA[]~~Unigene61785_Longan-RNA[]~~Unigene62191_Longan-RNA[]~~Unigene62694_Longan-RNA[]~~Unigene66882_Longan-RNA[]~~Unigene68648_Longan-RNA[]~~Unigene68909_Longan-RNA[] |
| miR1918 | 1 | Unigene9704_Longan-RNA[] |
| miR1919 | 6 | Unigene11599_Longan-RNA[]~~Unigene13392_Longan-RNA[]~~Unigene14561_Longan-RNA[]~~Unigene56430_Longan-RNA[]~~Unigene62891_Longan-RNA[]~~Unigene66738_Longan-RNA[] |
| miR2088 | 8 | Unigene9819_Longan-RNA[]~~Unigene11231_Longan-RNA[]~~Unigene43455_Longan-RNA[]~~Unigene55884_Longan-RNA[]~~Unigene60741_Longan-RNA[]~~Unigene65715_Longan-RNA[]~~Unigene67086_Longan-RNA[]~~Unigene68557_Longan-RNA[] |
| miR2091 | 5 | Unigene911_Longan-RNA[]~~Unigene59312_Longan-RNA[]~~Unigene59890_Longan-RNA[]~~Unigene16446_Longan-RNA[]~~Unigene23003_Longan-RNA[] |
| miR2092 | 5 | Unigene6554_Longan-RNA[]~~Unigene14312_Longan-RNA[]~~Unigene51494_Longan-RNA[]~~Unigene61271_Longan-RNA[]~~Unigene68697_Longan-RNA[] |
| miR2093 | 15 | Unigene2721_Longan-RNA[]~~Unigene9457_Longan-RNA[]~~Unigene10733_Longan-RNA[]~~Unigene13905_Longan-RNA[]~~Unigene14405_Longan-RNA[]~~Unigene15232_Longan-RNA[]~~Unigene19554_Longan-RNA[]~~Unigene36885_Longan-RNA[]~~Unigene61177_Longan-RNA[]~~Unigene62792_Longan-RNA[]~~Unigene67524_Longan-RNA[]~~Unigene67583_Longan-RNA[]~~Unigene67940_Longan-RNA[]~~Unigene68743_Longan-RNA[]~~Unigene11349_Longan-RNA[] |
| miR2097 | 2 | Unigene67266_Longan-RNA[]~~Unigene67552_Longan-RNA[] |
| miR2098 | 2 | Unigene15136_Longan-RNA[]~~Unigene62961_Longan-RNA[] |
| miR2099 | 26 | Unigene5002_Longan-RNA[]~~Unigene11860_Longan-RNA[]~~Unigene12199_Longan-RNA[]~~Unigene14219_Longan-RNA[]~~Unigene32842_Longan-RNA[]~~Unigene40923_Longan-RNA[]~~Unigene41105_Longan-RNA[]~~Unigene42345_Longan-RNA[]~~Unigene46304_Longan-RNA[]~~Unigene49578_Longan-RNA[]~~Unigene50004_Longan-RNA[]~~Unigene51901_Longan-RNA[]~~Unigene55243_Longan-RNA[]~~Unigene57502_Longan-RNA[]~~Unigene59296_Longan-RNA[]~~Unigene59355_Longan-RNA[]~~Unigene59399_Longan-RNA[]~~Unigene60294_Longan-RNA[]~~Unigene64044_Longan-RNA[]~~Unigene65025_Longan-RNA[]~~Unigene65345_Longan-RNA[]~~Unigene65821_Longan-RNA[]~~Unigene67316_Longan-RNA[]~~Unigene68780_Longan-RNA[]~~Unigene6881_Longan-RNA[]~~Unigene62211_Longan-RNA[] |
| miR2101 | 3 | Unigene57808_Longan-RNA[]~~Unigene64583_Longan-RNA[]~~Unigene68851_Longan-RNA[] |
| miR2108 | 8 | Unigene1398_Longan-RNA[]~~Unigene4229_Longan-RNA[]~~Unigene6499_Longan-RNA[]~~Unigene27936_Longan-RNA[]~~Unigene61298_Longan-RNA[]~~Unigene65609_Longan-RNA[]~~Unigene65807_Longan-RNA[]~~Unigene68744_Longan-RNA[] |
| miR2111 | 4 | Unigene2104_Longan-RNA[]~~Unigene60034_Longan-RNA[]~~Unigene66146_Longan-RNA[]~~Unigene66348_Longan-RNA[] |
| miR2118 | 1 | Unigene20455_Longan-RNA[] |
| miR2119 | 2 | Unigene5023_Longan-RNA[]~~Unigene10477_Longan-RNA[] |
| miR2595 | 3 | Unigene26394_Longan-RNA[]~~Unigene64720_Longan-RNA[]~~Unigene66150_Longan-RNA[] |
| miR2597 | 2 | Unigene10703_Longan-RNA[]~~Unigene34379_Longan-RNA[] |
| miR2604 | 1 | Unigene14561_Longan-RNA[] |
| miR2607 | 11 | Unigene9543_Longan-RNA[]~~Unigene11957_Longan-RNA[]~~Unigene24367_Longan-RNA[]~~Unigene32241_Longan-RNA[]~~Unigene53958_Longan-RNA[]~~Unigene61327_Longan-RNA[]~~Unigene63856_Longan-RNA[]~~Unigene64762_Longan-RNA[]~~Unigene64957_Longan-RNA[]~~Unigene68071_Longan-RNA[]~~Unigene68493_Longan-RNA[] |
| miR2610 | 4 | Unigene50830_Longan-RNA[]~~Unigene60395_Longan-RNA[]~~Unigene66039_Longan-RNA[]~~Unigene66263_Longan-RNA[] |
| miR2612 | 8 | Unigene911_Longan-RNA[]~~Unigene15036_Longan-RNA[]~~Unigene44314_Longan-RNA[]~~Unigene62707_Longan-RNA[]~~Unigene63172_Longan-RNA[]~~Unigene67885_Longan-RNA[]~~Unigene68767_Longan-RNA[]~~Unigene68849_Longan-RNA[] |
| miR2617 | 2 | Unigene15170_Longan-RNA[]~~Unigene60869_Longan-RNA[] |
| miR2620 | 1 | Unigene48229_Longan-RNA[] |
| miR2628 | 169 | Unigene766_Longan-RNA[]~~Unigene897_Longan-RNA[]~~Unigene1468_Longan-RNA[]~~Unigene1549_Longan-RNA[]~~Unigene1632_Longan-RNA[]~~Unigene2785_Longan-RNA[]~~Unigene2820_Longan-RNA[]~~Unigene2936_Longan-RNA[]~~Unigene4001_Longan-RNA[]~~Unigene4302_Longan-RNA[]~~Unigene4380_Longan-RNA[]~~Unigene5247_Longan-RNA[]~~Unigene6040_Longan-RNA[]~~Unigene6040_Longan-RNA[]~~Unigene6466_Longan-RNA[]~~Unigene6466_Longan-RNA[]~~Unigene6848_Longan-RNA[]~~Unigene7707_Longan-RNA[]~~Unigene8174_Longan-RNA[]~~Unigene8597_Longan-RNA[]~~Unigene10043_Longan-RNA[]~~Unigene10484_Longan-RNA[]~~Unigene11297_Longan-RNA[]~~Unigene11415_Longan-RNA[]~~Unigene11757_Longan-RNA[]~~Unigene11996_Longan-RNA[]~~Unigene11996_Longan-RNA[]~~Unigene12179_Longan-RNA[]~~Unigene12638_Longan-RNA[]~~Unigene12672_Longan-RNA[]~~Unigene12737_Longan-RNA[]~~Unigene13338_Longan-RNA[]~~Unigene13338_Longan-RNA[]~~Unigene13338_Longan-RNA[]~~Unigene13338_Longan-RNA[]~~Unigene13338_Longan-RNA[]~~Unigene13526_Longan-RNA[]~~Unigene13673_Longan-RNA[]~~Unigene13687_Longan-RNA[]~~Unigene13721Longan-RNA[]~~Unigene13946_Longan-RNA[]~~Unigene13984_Longan-RNA[]~~Unigene14144_Longan-RNA[]~~Unigene14232_Longan-RNA[]~~Unigene14925_Longan-RNA[]~~Unigene14955_Longan-RNA[]~~Unigene15045_Longan-RNA[]~~Unigene19043_Longan-RNA[]~~Unigene19357_Longan-RNA[]~~Unigene22736_Longan-RNA[]~~Unigene23533_Longan-RNA[]~~Unigene23533_Longan-RNA[]~~Unigene23533_Longan-RNA[]~~Unigene23533_Longan-RNA[]~~Unigene24236_Longan-RNA[]~~Unigene29020_Longan-RNA[]~~Unigene31031_Longan-RNA[]~~Unigene33617_Longan-RNA[]~~Unigene34561_Longan-RNA[]~~Unigene35578_Longan-RNA[]~~Unigene35833_Longan-RNA[]~~Unigene37672_Longan-RNA[]~~Unigene38183_Longan-RNA[]~~Unigene43292_Longan-RNA[]~~Unigene43292_Longan-RNA[]~~Unigene44386_Longan-RNA[]~~Unigene44564_Longan-RNA[]~~Unigene44737_Longan-RNA[]~~Unigene45105_Longan-RNA[]~~Unigene45931_Longan-RNA[]~~Unigene47398_Longan-RNA[]~~Unigene48146_Longan-RNA[]~~Unigene48163_Longan-RNA[]~~Unigene48457_Longan-RNA[]~~Unigene51319_Longan-RNA[]~~Unigene51572_Longan-RNA[]~~Unigene51927_Longan-RNA[]~~Unigene51927_Longan-RNA[]~~Unigene52434_Longan-RNA[]~~Unigene52635_Longan-RNA[]~~Unigene52904_Longan-RNA[]~~Unigene53141_Longan-RNA[]~~Unigene53483_Longan-RNA[]~~Unigene53959_Longan-RNA[]~~Unigene54739_Longan-RNA[]~~Unigene55701_Longan-RNA[]~~Unigene55859_Longan-RNA[]~~Unigene56085_Longan-RNA[]~~Unigene56183_Longan-RNA[]~~Unigene56604_Longan-RNA[]~~Unigene57179_Longan-RNA[]~~Unigene57401_Longan-RNA[]~~Unigene57632_Longan-RNA[]~~Unigene57747_Longan-RNA[]~~Unigene57807_Longan-RNA[]~~Unigene57952_Longan-RNA[]~~Unigene58219_Longan-RNA[]~~Unigene59015_Longan-RNA[]~~Unigene59059_Longan-RNA[]~~Unigene59126_Longan-RNA[]~~Unigene59526_Longan-RNA[]~~Unigene59587_Longan-RNA[]~~Unigene59608_Longan-RNA[]~~Unigene59982_Longan-RNA[]~~Unigene60062_Longan-RNA[]~~Unigene60365_Longan-RNA[]~~Unigene60491_Longan-RNA[]~~Unigene60504_Longan-RNA[]~~Unigene60927_Longan-RNA[]~~Unigene61041_Longan-RNA[]~~Unigene61297_Longan-RNA[]~~Unigene61401_Longan-RNA[]~~Unigene61429_Longan-RNA[]~~Unigene61467_Longan-RNA[]~~Unigene61849_Longan-RNA[]~~Unigene61960_Longan-RNA[]~~Unigene62000_Longan-RNA[]~~Unigene62201_Longan-RNA[]~~Unigene62746_Longan-RNA[]~~Unigene62795_Longan-RNA[]~~Unigene62997_Longan-RNA[]~~Unigene63669_Longan-RNA[]~~Unigene63858_Longan-RNA[]~~Unigene63979_Longan-RNA[]~~Unigene64211_Longan-RNA[]~~Unigene64667_Longan-RNA[]~~Unigene64855_Longan-RNA[]~~Unigene64931_Longan-RNA[]~~Unigene65092_Longan-RNA[]~~Unigene65215_Longan-RNA[]~~Unigene65330_Longan-RNA[]~~Unigene65372_Longan-RNA[]~~Unigene65627_Longan-RNA[]~~Unigene65650_Longan-RNA[]~~Unigene65850_Longan-RNA[]~~Unigene66156_Longan-RNA[]~~Unigene66184_Longan-RNA[]~~Unigene66203_Longan-RNA[]~~Unigene66264_Longan-RNA[]~~Unigene66338_Longan-RNA[]~~Unigene66432_Longan-RNA[]~~Unigene66881_Longan-RNA[]~~Unigene66915_Longan-RNA[]~~Unigene66946_Longan-RNA[]~~Unigene67012_Longan-RNA[]~~Unigene67066_Longan-RNA[]~~Unigene67154_Longan-RNA[]~~Unigene67327_Longan-RNA[]~~Unigene67360_Longan-RNA[]~~Unigene67456_Longan-RNA[]~~Unigene67495_Longan-RNA[]~~Unigene67579_Longan-RNA[]~~Unigene67618_Longan-RNA[]~~Unigene67737_Longan-RNA[]~~Unigene67785_Longan-RNA[]~~Unigene67790_Longan-RNA[]~~Unigene68119_Longan-RNA[]~~Unigene68246_Longan-RNA[]~~Unigene68269_Longan-RNA[]~~Unigene68437_Longan-RNA[]~~Unigene68532_Longan-RNA[]~~Unigene68590_Longan-RNA[]~~Unigene68617_Longan-RNA[]~~Unigene68709_Longan-RNA[]~~Unigene68788_Longan-RNA[]~~Unigene20053_Longan-RNA[]~~Unigene23300_Longan-RNA[]~~Unigene26378_Longan-RNA[]~~Unigene27389_Longan-RNA[] |
| miR2634 | 4 | Unigene17473_Longan-RNA[]~~Unigene64507_Longan-RNA[]~~Unigene68295_Longan-RNA[]~~Unigene43090_Longan-RNA[] |
| miR2641 | 28 | Unigene77_Longan-RNA[]~~Unigene8945_Longan-RNA[]~~Unigene12387_Longan-RNA[]~~Unigene12809_Longan-RNA[]~~Unigene23329_Longan-RNA[]~~Unigene41976_Longan-RNA[]~~Unigene45411_Longan-RNA[]~~Unigene46431_Longan-RNA[]~~Unigene47971_Longan-RNA[]~~Unigene49657_Longan-RNA[]~~Unigene55012_Longan-RNA[]~~Unigene56676_Longan-RNA[]~~Unigene60923_Longan-RNA[]~~Unigene60930_Longan-RNA[]~~Unigene62990_Longan-RNA[]~~Unigene63762_Longan-RNA[]~~Unigene64089_Longan-RNA[]~~Unigene64239_Longan-RNA[]~~Unigene64306_Longan-RNA[]~~Unigene64693_Longan-RNA[]~~Unigene66092_Longan-RNA[]~~Unigene66726_Longan-RNA[]~~Unigene67337_Longan-RNA[]~~Unigene67399_Longan-RNA[]~~Unigene67466_Longan-RNA[]~~Unigene68466_Longan-RNA[]~~Unigene11248_Longan-RNA[]~~Unigene22587_Longan-RNA[] |
| miR2642 | 21 | Unigene10566_Longan-RNA[]~~Unigene10970_Longan-RNA[]~~Unigene11544_Longan-RNA[]~~Unigene12295_Longan-RNA[]~~Unigene13248_Longan-RNA[]~~Unigene13937_Longan-RNA[]~~Unigene50114_Longan-RNA[]~~Unigene51134_Longan-RNA[]~~Unigene54388_Longan-RNA[]~~Unigene59532_Longan-RNA[]~~Unigene62885_Longan-RNA[]~~Unigene64783_Longan-RNA[]~~Unigene65219_Longan-RNA[]~~Unigene67677_Longan-RNA[]~~Unigene68031_Longan-RNA[]~~Unigene68033_Longan-RNA[]~~Unigene68055_Longan-RNA[]~~Unigene68068_Longan-RNA[]~~Unigene68227_Longan-RNA[]~~Unigene68575_Longan-RNA[]~~Unigene36103_Longan-RNA[] |
| miR2643 | 26 | Unigene3532_Longan-RNA[]~~Unigene6419_Longan-RNA[]~~Unigene9581_Longan-RNA[]~~Unigene9649_Longan-RNA[]~~Unigene11934_Longan-RNA[]~~Unigene12290_Longan-RNA[]~~Unigene16032_Longan-RNA[]~~Unigene45825_Longan-RNA[]~~Unigene52089_Longan-RNA[]~~Unigene52529_Longan-RNA[]~~Unigene53530_Longan-RNA[]~~Unigene56644_Longan-RNA[]~~Unigene57010_Longan-RNA[]~~Unigene57914_Longan-RNA[]~~Unigene59067_Longan-RNA[]~~Unigene59820_Longan-RNA[]~~Unigene63478_Longan-RNA[]~~Unigene63513_Longan-RNA[]~~Unigene63599_Longan-RNA[]~~Unigene64997_Longan-RNA[]~~Unigene66771_Longan-RNA[]~~Unigene66928_Longan-RNA[]~~Unigene67813_Longan-RNA[]~~Unigene67961_Longan-RNA[]~~Unigene67986_Longan-RNA[]~~Unigene1616_Longan-RNA[] |
| miR2644 | 4 | Unigene11167_Longan-RNA[]~~Unigene57046_Longan-RNA[]~~Unigene65239_Longan-RNA[]~~Unigene68391_Longan-RNA[] |
| miR2651 | 8 | Unigene2277_Longan-RNA[]~~Unigene4052_Longan-RNA[]~~Unigene5160_Longan-RNA[]~~Unigene52292_Longan-RNA[]~~Unigene54162_Longan-RNA[]~~Unigene59291_Longan-RNA[]~~Unigene65087_Longan-RNA[]~~Unigene65797_Longan-RNA[] |
| miR2653 | 1 | Unigene37_Longan-RNA[] |
| miR2657 | 1 | Unigene40789_Longan-RNA[] |
| miR2658 | 15 | Unigene930_Longan-RNA[]~~Unigene4277_Longan-RNA[]~~Unigene10421_Longan-RNA[]~~Unigene20620_Longan-RNA[]~~Unigene22518_Longan-RNA[]~~Unigene32675_Longan-RNA[]~~Unigene51674_Longan-RNA[]~~Unigene56713_Longan-RNA[]~~Unigene56837_Longan-RNA[]~~Unigene56897_Longan-RNA[]~~Unigene66945_Longan-RNA[]~~Unigene67443_Longan-RNA[]~~Unigene67481_Longan-RNA[]~~Unigene68152_Longan-RNA[]~~Unigene68426_Longan-RNA[] |
| miR2661 | 6 | Unigene550_Longan-RNA[]~~Unigene13639_Longan-RNA[]~~Unigene63490_Longan-RNA[]~~Unigene64875_Longan-RNA[]~~Unigene68626_Longan-RNA[]~~Unigene68826_Longan-RNA[] |
| miR2663 | 1 | Unigene15111_Longan-RNA[] |
| miR2667 | 17 | Unigene5588_Longan-RNA[]~~Unigene13302_Longan-RNA[]~~Unigene13441_Longan-RNA[]~~Unigene14046_Longan-RNA[]~~Unigene19862_Longan-RNA[]~~Unigene33192_Longan-RNA[]~~Unigene33454_Longan-RNA[]~~Unigene54598_Longan-RNA[]~~Unigene55091_Longan-RNA[]~~Unigene58741_Longan-RNA[]~~Unigene60994_Longan-RNA[]~~Unigene61361_Longan-RNA[]~~Unigene64419_Longan-RNA[]~~Unigene64609_Longan-RNA[]~~Unigene65061_Longan-RNA[]~~Unigene68332_Longan-RNA[]~~Unigene68489_Longan-RNA[] |
| miR2670 | 2 | Unigene38316_Longan-RNA[]~~Unigene62449_Longan-RNA[] |
| miR2673 | 118 | Unigene2021_Longan-RNA[]~~Unigene2549_Longan-RNA[]~~Unigene2620_Longan-RNA[]~~Unigene3881_Longan-RNA[]~~Unigene5211_Longan-RNA[]~~Unigene5424_Longan-RNA[]~~Unigene5728_Longan-RNA[]~~Unigene6804_Longan-RNA[]~~Unigene6998_Longan-RNA[]~~Unigene8611_Longan-RNA[]~~Unigene8639_Longan-RNA[]~~Unigene8639_Longan-RNA[]~~Unigene9976_Longan-RNA[]~~Unigene10690_Longan-RNA[]~~Unigene11031_Longan-RNA[]~~Unigene11085_Longan-RNA[]~~Unigene11676_Longan-RNA[]~~Unigene11682_Longan-RNA[]~~Unigene12324_Longan-RNA[]~~Unigene12483_Longan-RNA[]~~Unigene12825_Longan-RNA[]~~Unigene12941_Longan-RNA[]~~Unigene14203_Longan-RNA[]~~Unigene14376_Longan-RNA[]~~Unigene14472_Longan-RNA[]~~Unigene14550_Longan-RNA[]~~Unigene14791_Longan-RNA[]~~Unigene15006_Longan-RNA[]~~Unigene15184_Longan-RNA[]~~Unigene15499_Longan-RNA[]~~Unigene16930_Longan-RNA[]~~Unigene17505_Longan-RNA[]~~Unigene18621_Longan-RNA[]~~Unigene26040_Longan-RNA[]~~Unigene26965_Longan-RNA[]~~Unigene29256_Longan-RNA[]~~Unigene29510_Longan-RNA[]~~Unigene31583_Longan-RNA[]~~Unigene32941_Longan-RNA[]~~Unigene40014_Longan-RNA[]~~Unigene40331_Longan-RNA[]~~Unigene41544_Longan-RNA[]~~Unigene42912_Longan-RNA[]~~Unigene43431_Longan-RNA[]~~Unigene43911_Longan-RNA[]~~Unigene45135_Longan-RNA[]~~Unigene46314_Longan-RNA[]~~Unigene48589_Longan-RNA[]~~Unigene49256_Longan-RNA[]~~Unigene49629_Longan-RNA[]~~Unigene50280_Longan-RNA[]~~Unigene50867_Longan-RNA[]~~Unigene51675_Longan-RNA[]~~Unigene52213_Longan-RNA[]~~Unigene52884_Longan-RNA[]~~Unigene52905_Longan-RNA[]~~Unigene53192_Longan-RNA[]~~Unigene53293_Longan-RNA[]~~Unigene53324_Longan-RNA[]~~Unigene53340_Longan-RNA[]~~Unigene54104_Longan-RNA[]~~Unigene54142_Longan-RNA[]~~Unigene54770_Longan-RNA[]~~Unigene57309_Longan-RNA[]~~Unigene58533_Longan-RNA[]~~Unigene58789_Longan-RNA[]~~Unigene59395_Longan-RNA[]~~Unigene59626_Longan-RNA[]~~Unigene59739_Longan-RNA[]~~Unigene59876_Longan-RNA[]~~Unigene60784_Longan-RNA[]~~Unigene61029_Longan-RNA[]~~Unigene61396_Longan-RNA[]~~Unigene61784_Longan-RNA[]~~Unigene61988_Longan-RNA[]~~Unigene62009_Longan-RNA[]~~Unigene62405_Longan-RNA[]~~Unigene62826_Longan-RNA[]~~Unigene63072_Longan-RNA[]~~Unigene63119_Longan-RNA[]~~Unigene63538_Longan-RNA[]~~Unigene63600_Longan-RNA[]~~Unigene63621_Longan-RNA[]~~Unigene64659_Longan-RNA[]~~Unigene64724_Longan-RNA[]~~Unigene64841_Longan-RNA[]~~Unigene65012_Longan-RNA[]~~Unigene65086_Longan-RNA[]~~Unigene65692_Longan-RNA[]~~Unigene66424_Longan-RNA[]~~Unigene66425_Longan-RNA[]~~Unigene66454_Longan-RNA[]~~Unigene66512_Longan-RNA[]~~Unigene66947_Longan-RNA[]~~Unigene66979_Longan-RNA[]~~Unigene66991_Longan-RNA[]~~Unigene67034_Longan-RNA[]~~Unigene67235_Longan-RNA[]~~Unigene67563_Longan-RNA[]~~Unigene67649_Longan-RNA[]~~Unigene67910_Longan-RNA[]~~Unigene67954_Longan-RNA[]~~Unigene68084_Longan-RNA[]~~Unigene68296_Longan-RNA[]~~Unigene68371_Longan-RNA[]~~Unigene68461_Longan-RNA[]~~Unigene68462_Longan-RNA[]~~Unigene68631_Longan-RNA[]~~Unigene68818_Longan-RNA[]~~Unigene68910_Longan-RNA[]~~Unigene16010_Longan-RNA[]~~Unigene16010_Longan-RNA[]~~Unigene19879_Longan-RNA[]~~Unigene26777_Longan-RNA[]~~Unigene32363_Longan-RNA[]~~Unigene40788_Longan-RNA[]~~Unigene51225_Longan-RNA[]~~Unigene51632_Longan-RNA[] |
| miR2676 | 4 | Unigene14487_Longan-RNA[]~~Unigene63825_Longan-RNA[]~~Unigene64037_Longan-RNA[]~~Unigene67297_Longan-RNA[] |
| miR2678 | 10 | Unigene10773_Longan-RNA[]~~Unigene26824_Longan-RNA[]~~Unigene39177_Longan-RNA[]~~Unigene52859_Longan-RNA[]~~Unigene55839_Longan-RNA[]~~Unigene62106_Longan-RNA[]~~Unigene62558_Longan-RNA[]~~Unigene63824_Longan-RNA[]~~Unigene67893_Longan-RNA[]~~Unigene68341_Longan-RNA[] |
| miR2863 | 1 | Unigene67083_Longan-RNA[] |
| miR2864 | 3 | Unigene5413_Longan-RNA[]~~Unigene52745_Longan-RNA[]~~Unigene59847_Longan-RNA[] |
| miR2870 | 4 | Unigene7063_Longan-RNA[]~~Unigene44100_Longan-RNA[]~~Unigene46174_Longan-RNA[]~~Unigene59498_Longan-RNA[] |
| miR2911 | 3 | Unigene11953_Longan-RNA[]~~Unigene42867_Longan-RNA[]~~Unigene47204_Longan-RNA[] |
| miR2922 | 1 | Unigene44699_Longan-RNA[] |
| miR2931 | 10 | Unigene11233_Longan-RNA[]~~Unigene11370_Longan-RNA[]~~Unigene12335_Longan-RNA[]~~Unigene12437_Longan-RNA[]~~Unigene14146_Longan-RNA[]~~Unigene17954_Longan-RNA[]~~Unigene54934_Longan-RNA[]~~Unigene62995_Longan-RNA[]~~Unigene66722_Longan-RNA[]~~Unigene67105_Longan-RNA[] |
| miR2933 | 16 | Unigene8142_Longan-RNA[]~~Unigene8152_Longan-RNA[]~~Unigene11548_Longan-RNA[]~~Unigene11886_Longan-RNA[]~~Unigene12631_Longan-RNA[]~~Unigene41800_Longan-RNA[]~~Unigene48697_Longan-RNA[]~~Unigene48800_Longan-RNA[]~~Unigene58797_Longan-RNA[]~~Unigene59891_Longan-RNA[]~~Unigene62628_Longan-RNA[]~~Unigene63485_Longan-RNA[]~~Unigene64248_Longan-RNA[]~~Unigene64646_Longan-RNA[]~~Unigene65361_Longan-RNA[]~~Unigene39833_Longan-RNA[] |
| miR2936 | 1 | Unigene14730_Longan-RNA[] |
| miR2937 | 1 | Unigene10779_Longan-RNA[] |
| miR2938 | 2 | Unigene9619_Longan-RNA[]~~Unigene57903_Longan-RNA[] |
| miR2948 | 2 | Unigene4442_Longan-RNA[]~~Unigene5320_Longan-RNA[] |
| miR3433 | 4 | Unigene8452_Longan-RNA[]~~Unigene12494_Longan-RNA[]~~Unigene45993_Longan-RNA[]~~Unigene68924_Longan-RNA[] |
| miR3435 | 8 | Unigene14585_Longan-RNA[]~~Unigene14754_Longan-RNA[]~~Unigene46518_Longan-RNA[]~~Unigene47155_Longan-RNA[]~~Unigene52962_Longan-RNA[]~~Unigene63137_Longan-RNA[]~~Unigene68217_Longan-RNA[]~~Unigene31912_Longan-RNA[] |
| miR3437 | 9 | Unigene2056_Longan-RNA[]~~Unigene2926_Longan-RNA[]~~Unigene52877_Longan-RNA[]~~Unigene58541_Longan-RNA[]~~Unigene60529_Longan-RNA[]~~Unigene62264_Longan-RNA[]~~Unigene64408_Longan-RNA[]~~Unigene65561_Longan-RNA[]~~Unigene66091_Longan-RNA[] |
| miR3439 | 4 | Unigene4052_Longan-RNA[]~~Unigene8174_Longan-RNA[]~~Unigene29326_Longan-RNA[]~~Unigene68804_Longan-RNA[] |
| miR3440 | 17 | Unigene445_Longan-RNA[]~~Unigene4663_Longan-RNA[]~~Unigene7952_Longan-RNA[]~~Unigene10477_Longan-RNA[]~~Unigene13336_Longan-RNA[]~~Unigene14529_Longan-RNA[]~~Unigene14587_Longan-RNA[]~~Unigene15001_Longan-RNA[]~~Unigene15161_Longan-RNA[]~~Unigene31903_Longan-RNA[]~~Unigene53268_Longan-RNA[]~~Unigene54235_Longan-RNA[]~~Unigene61562_Longan-RNA[]~~Unigene62338_Longan-RNA[]~~Unigene64998_Longan-RNA[]~~Unigene66133_Longan-RNA[]~~Unigene66802_Longan-RNA[] |
| miR3441 | 2 | Unigene34935_Longan-RNA[]~~Unigene57053_Longan-RNA[] |
| miR3442 | 18 | Unigene307_Longan-RNA[]~~Unigene4102_Longan-RNA[]~~Unigene5300_Longan-RNA[]~~Unigene11013_Longan-RNA[]~~Unigene12828_Longan-RNA[]~~Unigene13680_Longan-RNA[]~~Unigene13871_Longan-RNA[]~~Unigene15084_Longan-RNA[]~~Unigene15133_Longan-RNA[]~~Unigene51597_Longan-RNA[]~~Unigene57388_Longan-RNA[]~~Unigene58931_Longan-RNA[]~~Unigene62531_Longan-RNA[]~~Unigene65294_Longan-RNA[]~~Unigene66605_Longan-RNA[]~~Unigene68493_Longan-RNA[]~~Unigene68516_Longan-RNA[]~~Unigene68615_Longan-RNA[] |
| miR3444 | 8 | Unigene14972_Longan-RNA[]~~Unigene49338_Longan-RNA[]~~Unigene53124_Longan-RNA[]~~Unigene62741_Longan-RNA[]~~Unigene64239_Longan-RNA[]~~Unigene65958_Longan-RNA[]~~Unigene66758_Longan-RNA[]~~Unigene68488_Longan-RNA[] |
| miR3452 | 7 | Unigene13597_Longan-RNA[]~~Unigene43742_Longan-RNA[]~~Unigene56816_Longan-RNA[]~~Unigene62658_Longan-RNA[]~~Unigene65489_Longan-RNA[]~~Unigene67129_Longan-RNA[]~~Unigene68326_Longan-RNA[] |
| miR3453 | 1 | Unigene43713_Longan-RNA[] |
| miR3454 | 2 | Unigene14603_Longan-RNA[]~~Unigene58632_Longan-RNA[] |
| miR3458 | 9 | Unigene518_Longan-RNA[]~~Unigene1422_Longan-RNA[]~~Unigene7822_Longan-RNA[]~~Unigene10962_Longan-RNA[]~~Unigene52671_Longan-RNA[]~~Unigene54061_Longan-RNA[]~~Unigene57181_Longan-RNA[]~~Unigene57841_Longan-RNA[]~~Unigene58544_Longan-RNA[] |
| miR3512 | 2 | Unigene15148_Longan-RNA[]~~Unigene68608_Longan-RNA[] |
| miR3515 | 12 | Unigene1117_Longan-RNA[]~~Unigene14821_Longan-RNA[]~~Unigene14850_Longan-RNA[]~~Unigene32638_Longan-RNA[]~~Unigene33641_Longan-RNA[]~~Unigene53890_Longan-RNA[]~~Unigene54687_Longan-RNA[]~~Unigene55211_Longan-RNA[]~~Unigene63887_Longan-RNA[]~~Unigene65763_Longan-RNA[]~~Unigene67470_Longan-RNA[]~~Unigene67913_Longan-RNA[] |
| miR3519 | 2 | Unigene53382_Longan-RNA[]~~Unigene54625_Longan-RNA[] |
| miR3522 | 1 | Unigene68779_Longan-RNA[] |
| miR3623 | 3 | Unigene1117_Longan-RNA[]~~Unigene11219_Longan-RNA[]~~Unigene48517_Longan-RNA[] |
| miR3624 | 4 | Unigene61262_Longan-RNA[]~~Unigene66339_Longan-RNA[]~~Unigene67615_Longan-RNA[]~~Unigene68175_Longan-RNA[] |
| miR3625 | 1 | Unigene14278_Longan-RNA[] |
| miR3629 | 1 | Unigene60021_Longan-RNA[] |
| miR3630 | 7 | Unigene51595_Longan-RNA[]~~Unigene55685_Longan-RNA[]~~Unigene63169_Longan-RNA[]~~Unigene65824_Longan-RNA[]~~Unigene68851_Longan-RNA[]~~Unigene68909_Longan-RNA[]~~Unigene65378_Longan-RNA[] |
| miR3633 | 2 | Unigene28914_Longan-RNA[]~~Unigene64605_Longan-RNA[] |
| miR3639 | 1 | Unigene13983_Longan-RNA[] |
| miR3640 | 1 | Unigene68181_Longan-RNA[] |
| miR3699 | 2 | Unigene43919_Longan-RNA[]~~Unigene62319_Longan-RNA[] |
| miR3705 | 3 | Unigene12595_Longan-RNA[]~~Unigene65299_Longan-RNA[]~~Unigene68919_Longan-RNA[] |
| miR3933 | 1 | Unigene14793_Longan-RNA[] |
| miR3946 | 1 | Unigene39935_Longan-RNA[] |
| miR3949 | 7 | Unigene3945_Longan-RNA[]~~Unigene5529_Longan-RNA[]~~Unigene41622_Longan-RNA[]~~Unigene44161_Longan-RNA[]~~Unigene50852_Longan-RNA[]~~Unigene53793_Longan-RNA[]~~Unigene68471_Longan-RNA[] |
| miR3979 | 5 | Unigene13869_Longan-RNA[]~~Unigene15236_Longan-RNA[]~~Unigene21347_Longan-RNA[]~~Unigene33600_Longan-RNA[]~~Unigene57474_Longan-RNA[] |
| miR3981 | 5 | Unigene14246_Longan-RNA[]~~Unigene14697_Longan-RNA[]~~Unigene47192_Longan-RNA[]~~Unigene50182_Longan-RNA[]~~Unigene64740_Longan-RNA[] |
| miR4233 | 1 | Unigene56450_Longan-RNA[] |
| miR4244 | 6 | Unigene3394_Longan-RNA[]~~Unigene7379_Longan-RNA[]~~Unigene9935_Longan-RNA[]~~Unigene11588_Longan-RNA[]~~Unigene62166_Longan-RNA[]~~Unigene20235_Longan-RNA[] |
| miR4248 | 3 | Unigene41964_Longan-RNA[]~~Unigene49921_Longan-RNA[]~~Unigene68875_Longan-RNA[] |
| miR4345 | 1 | Unigene53423_Longan-RNA[] |
| miR4361 | 2 | Unigene12824_Longan-RNA[]~~Unigene66902_Longan-RNA[] |
| miR4362 | 5 | Unigene36336_Longan-RNA[]~~Unigene38765_Longan-RNA[]~~Unigene54159_Longan-RNA[]~~Unigene67273_Longan-RNA[]~~Unigene67606_Longan-RNA[] |
| miR4365 | 3 | Unigene9443_Longan-RNA[]~~Unigene10998_Longan-RNA[]~~Unigene61936_Longan-RNA[] |
| miR4371 | 1 | Unigene53814_Longan-RNA[] |
| miR4378 | 1 | Unigene63528_Longan-RNA[] |
| miR4386 | 3 | Unigene14550_Longan-RNA[]~~Unigene53615_Longan-RNA[]~~Unigene68353_Longan-RNA[] |
| miR4393 | 1 | Unigene66023_Longan-RNA[] |
| miR4397 | 2 | Unigene12122_Longan-RNA[]~~Unigene64173_Longan-RNA[] |
| miR4398 | 2 | Unigene9083_Longan-RNA[]~~Unigene14955_Longan-RNA[] |
| miR4413 | 12 | Unigene2794_Longan-RNA[]~~Unigene5682_Longan-RNA[]~~Unigene9587_Longan-RNA[]~~Unigene14191_Longan-RNA[]~~Unigene14215_Longan-RNA[]~~Unigene14497_Longan-RNA[]~~Unigene54959_Longan-RNA[]~~Unigene57052_Longan-RNA[]~~Unigene60829_Longan-RNA[]~~Unigene61639_Longan-RNA[]~~Unigene61920_Longan-RNA[]~~Unigene66976_Longan-RNA[] |
| miR5012 | 3 | Unigene35276_Longan-RNA[]~~Unigene42631_Longan-RNA[]~~Unigene53019_Longan-RNA[] |
| miR5014 | 24 | Unigene363_Longan-RNA[]~~Unigene5677_Longan-RNA[]~~Unigene6712_Longan-RNA[]~~Unigene9581_Longan-RNA[]~~Unigene12400_Longan-RNA[]~~Unigene12567_Longan-RNA[]~~Unigene15052_Longan-RNA[]~~Unigene41191_Longan-RNA[]~~Unigene44645_Longan-RNA[]~~Unigene44943_Longan-RNA[]~~Unigene49196_Longan-RNA[]~~Unigene53356_Longan-RNA[]~~Unigene65686_Longan-RNA[]~~Unigene66808_Longan-RNA[]~~Unigene66951_Longan-RNA[]~~Unigene67177_Longan-RNA[]~~Unigene67212_Longan-RNA[]~~Unigene67732_Longan-RNA[]~~Unigene68116_Longan-RNA[]~~Unigene68227_Longan-RNA[]~~Unigene3556_Longan-RNA[]~~Unigene23443_Longan-RNA[]~~Unigene36834_Longan-RNA[]~~Unigene66250_Longan-RNA[] |
| miR5015 | 1 | Unigene11508_Longan-RNA[] |
| miR5020 | 9 | Unigene15049_Longan-RNA[]~~Unigene15161_Longan-RNA[]~~Unigene42319_Longan-RNA[]~~Unigene49403_Longan-RNA[]~~Unigene51649_Longan-RNA[]~~Unigene54927_Longan-RNA[]~~Unigene62364_Longan-RNA[]~~Unigene62926_Longan-RNA[]~~Unigene65158_Longan-RNA[] |
| miR5021 | 28 | Unigene162_Longan-RNA[]~~Unigene798_Longan-RNA[]~~Unigene1117_Longan-RNA[]~~Unigene6382_Longan-RNA[]~~Unigene6566_Longan-RNA[]~~Unigene11129_Longan-RNA[]~~Unigene11640_Longan-RNA[]~~Unigene14282_Longan-RNA[]~~Unigene14874_Longan-RNA[]~~Unigene16595_Longan-RNA[]~~Unigene16595_Longan-RNA[]~~Unigene24510_Longan-RNA[]~~Unigene35578_Longan-RNA[]~~Unigene39941_Longan-RNA[]~~Unigene45907_Longan-RNA[]~~Unigene48514_Longan-RNA[]~~Unigene57560_Longan-RNA[]~~Unigene59927_Longan-RNA[]~~Unigene63406_Longan-RNA[]~~Unigene64605_Longan-RNA[]~~Unigene66036_Longan-RNA[]~~Unigene66579_Longan-RNA[]~~Unigene67808_Longan-RNA[]~~Unigene68514_Longan-RNA[]~~Unigene68593_Longan-RNA[]~~Unigene68717_Longan-RNA[]~~Unigene16949_Longan-RNA[]~~Unigene36638_Longan-RNA[] |
| miR5023 | 1 | Unigene33744_Longan-RNA[] |
| miR5028 | 1 | Unigene12478_Longan-RNA[] |
| miR5052 | 1 | Unigene61327_Longan-RNA[] |
| miR5054 | 1 | Unigene65129_Longan-RNA[] |
| miR5057 | 8 | Unigene6700_Longan-RNA[]~~Unigene46891_Longan-RNA[]~~Unigene48422_Longan-RNA[]~~Unigene63273_Longan-RNA[]~~Unigene65400_Longan-RNA[]~~Unigene66837_Longan-RNA[]~~Unigene67108_Longan-RNA[]~~Unigene67258_Longan-RNA[] |
| miR5066 | 6 | Unigene5231_Longan-RNA[]~~Unigene5901_Longan-RNA[]~~Unigene10316_Longan-RNA[]~~Unigene14903_Longan-RNA[]~~Unigene15249_Longan-RNA[]~~Unigene56517_Longan-RNA[] |
| miR5071 | 4 | Unigene929_Longan-RNA[]~~Unigene2094_Longan-RNA[]~~Unigene67250_Longan-RNA[]~~Unigene67294_Longan-RNA[] |
| miR5074 | 3 | Unigene44999_Longan-RNA[]~~Unigene61376_Longan-RNA[]~~Unigene68351_Longan-RNA[] |
| miR5077 | 12 | Unigene11074_Longan-RNA[]~~Unigene12400_Longan-RNA[]~~Unigene14494_Longan-RNA[]~~Unigene21628_Longan-RNA[]~~Unigene23937_Longan-RNA[]~~Unigene47263_Longan-RNA[]~~Unigene60539_Longan-RNA[]~~Unigene62162_Longan-RNA[]~~Unigene66794_Longan-RNA[]~~Unigene68245_Longan-RNA[]~~Unigene68748_Longan-RNA[]~~Unigene68852_Longan-RNA[] |
| miR5078 | 5 | Unigene6449_Longan-RNA[]~~Unigene30875_Longan-RNA[]~~Unigene38743_Longan-RNA[]~~Unigene62446_Longan-RNA[]~~Unigene68246_Longan-RNA[] |
| miR5079 | 22 | Unigene3399_Longan-RNA[]~~Unigene8631_Longan-RNA[]~~Unigene11671_Longan-RNA[]~~Unigene12465_Longan-RNA[]~~Unigene15171_Longan-RNA[]~~Unigene25224_Longan-RNA[]~~Unigene31501_Longan-RNA[]~~Unigene35206_Longan-RNA[]~~Unigene38130_Longan-RNA[]~~Unigene39008_Longan-RNA[]~~Unigene39339_Longan-RNA[]~~Unigene41457_Longan-RNA[]~~Unigene41736_Longan-RNA[]~~Unigene46146_Longan-RNA[]~~Unigene54839_Longan-RNA[]~~Unigene55962_Longan-RNA[]~~Unigene59337_Longan-RNA[]~~Unigene59552_Longan-RNA[]~~Unigene59708_Longan-RNA[]~~Unigene60138_Longan-RNA[]~~Unigene60895_Longan-RNA[]~~Unigene63618_Longan-RNA[] |
| miR5085 | 10 | Unigene14613_Longan-RNA[]~~Unigene44401_Longan-RNA[]~~Unigene57130_Longan-RNA[]~~Unigene58122_Longan-RNA[]~~Unigene64957_Longan-RNA[]~~Unigene67244_Longan-RNA[]~~Unigene67940_Longan-RNA[]~~Unigene68150_Longan-RNA[]~~Unigene68917_Longan-RNA[]~~Unigene31146_Longan-RNA[] |
| miR5137 | 6 | Unigene6004_Longan-RNA[]~~Unigene11599_Longan-RNA[]~~Unigene43827_Longan-RNA[]~~Unigene50360_Longan-RNA[]~~Unigene53214_Longan-RNA[]~~Unigene56126_Longan-RNA[] |
| miR5138 | 2 | Unigene38442_Longan-RNA[]~~Unigene59745_Longan-RNA[] |
| miR5139 | 1 | Unigene38481_Longan-RNA[] |
| miR5140 | 8 | Unigene6798_Longan-RNA[]~~Unigene13968_Longan-RNA[]~~Unigene37818_Longan-RNA[]~~Unigene51169_Longan-RNA[]~~Unigene52491_Longan-RNA[]~~Unigene59143_Longan-RNA[]~~Unigene59204_Longan-RNA[]~~Unigene67793_Longan-RNA[] |
| miR5142 | 4 | Unigene23466_Longan-RNA[]~~Unigene44134_Longan-RNA[]~~Unigene58560_Longan-RNA[]~~Unigene67301_Longan-RNA[] |
| miR5163 | 2 | Unigene65128_Longan-RNA[]~~Unigene65529_Longan-RNA[] |
| miR5166 | 1 | Unigene65302_Longan-RNA[] |
| miR5168 | 3 | Unigene13401_Longan-RNA[]~~Unigene57057_Longan-RNA[]~~Unigene63706_Longan-RNA[] |
| miR5169 | 1 | Unigene14227_Longan-RNA[] |
| miR5170 | 17 | Unigene6676_Longan-RNA[]~~Unigene10808_Longan-RNA[]~~Unigene13916_Longan-RNA[]~~Unigene15175_Longan-RNA[]~~Unigene47966_Longan-RNA[]~~Unigene48519_Longan-RNA[]~~Unigene52811_Longan-RNA[]~~Unigene57725_Longan-RNA[]~~Unigene58146_Longan-RNA[]~~Unigene59421_Longan-RNA[]~~Unigene59733_Longan-RNA[]~~Unigene60263_Longan-RNA[]~~Unigene61204_Longan-RNA[]~~Unigene64608_Longan-RNA[]~~Unigene65570_Longan-RNA[]~~Unigene66476_Longan-RNA[]~~Unigene68626_Longan-RNA[] |
| miR5171 | 8 | Unigene224_Longan-RNA[]~~Unigene7441_Longan-RNA[]~~Unigene7800_Longan-RNA[]~~Unigene64585_Longan-RNA[]~~Unigene66921_Longan-RNA[]~~Unigene68330_Longan-RNA[]~~Unigene68682_Longan-RNA[]~~Unigene68874_Longan-RNA[] |
| miR5177 | 1 | Unigene64731_Longan-RNA[] |
| miR5182 | 3 | Unigene51381_Longan-RNA[]~~Unigene51985_Longan-RNA[]~~Unigene62555_Longan-RNA[] |
| miR5185 | 3 | Unigene6919_Longan-RNA[]~~Unigene33839_Longan-RNA[]~~Unigene67927_Longan-RNA[] |
| miR5198 | 1 | Unigene52143_Longan-RNA[] |
| miR5202 | 1 | Unigene6747_Longan-RNA[] |
| miR5300 | 1 | Unigene63704_Longan-RNA[] |
