# Supplementary material for: Comparative Analysis Reveals Dynamic Changes in miRNAs and Their Targets and Expression during Somatic Embryogenesis in Longan (Dimocarpus longan Lour.)
Source: PLoS One. 2013 Apr 11;8(4):e60337. doi: 10.1371/journal.pone.0060337 (PMC3623967; doi:10.1371/journal.pone.0060337)
Supplement: Supporting Information S1 File containing the following supporting information tables — Table S1. Coserved and non-coserved longan miRNAs. Table S2. Longan-known miRNA targets by predicition method. Table S3. Identifiction targets for longan miRNAs by Degradome sequencing. Table S4. The consensus targets for longan miRNAs obtaining from prediction and degradome analysis. Table S5. Primer sequences. Table S6. The miRNA names, primer sequences. (ZIP) [file pone.0060337.s001.zip › Supporting Information files/Table S3 Identifiction targets for longan miRNAs by Degradome sequencing.doc]

| **Table S3 Identifiction targets for longan miRNAs by Degradome sequencing** | | | | |
| --- | --- | --- | --- | --- |
| miRNAs | Target genes | splice sites | value of normalization | Description |
| dlo-miR1 | Unigene67560_Longan-RNA:1:1406:+ | 349 | 15.3812 | alpha-biotin transcarboxylase subunit |
|  |  | 1276 | 12.5846 |  |
|  | Unigene2488_Longan-RNA:1:480:+ | 122 | 10.4872 | conserved hypothetical protein |
|  |  | 128 | 16.0803 |  |
|  |  | 135 | 12.5846 |  |
|  | Unigene57312_Longan-RNA:1:485:+ | 175 | 46.8426 | non-race specific disease resistance protein 1-like protein b |
|  |  | 176 | 26.2179 |  |
| dlo-miR2 | Unigene57180_Longan-RNA:1:481:+ | 418 | 12.235 | hydroxyproline-rich glycoprotein family protein |
|  |  | 420 | 69.2152 |  |
|  |  | 421 | 12.235 |  |
| dlo-miR5 | Unigene50565_Longan-RNA:1:356:+ | 168 | 19.925 | ribophorin II family protein |
| miR157 | Unigene11990_Longan-RNA:1:1171:+ | 211 | 19.2265 | transcription factor C2H2 |
|  | Unigene62013_Longan-RNA:1:663:+ | 472 | 12.5846 | f-box family protein |
| miR157,miR156 | Unigene58663_Longan-RNA:1:524:+ | 351 | 25.1692 | DNA-binding bromodomain-containing protein |
| miR158 | Unigene57300_Longan-RNA:1:485:+ | 1 | 19.9256 | F1N21.5 |
| miR159 | Unigene12638_Longan-RNA:1:2078:+ | 204 | 18.5273 | At4g17430 |
| miR167 | Unigene4432_Longan-RNA:1:211:+ | 119 | 23.4213 |  |
|  |  | 120 | 20.2752 |  |
| miR390 | Unigene47404_Longan-RNA:1:316:+ | 185 | 15.7307 | RKF3 (RECEPTOR-LIKE KINASE IN IN FLOWERS 3); kinase/ receptor signaling protein serine/threonine kinase |
| miR393 | Unigene39139_Longan-RNA:1:250:+ | 62 | 29.364 | glycosyltransferase, CAZy family GT8 |
| miR396 | Unigene68412_Longan-RNA:1:1903:+ | 802 | 35.656 | Protein FAR1-RELATED SEQUENCE 9 |
|  | Unigene8340_Longan-RNA:1:1645:+ | 1447 | 12.5846 |  |
| miR397 | Unigene66334_Longan-RNA:1:1082:+ | 636 | 89.84 | sister-chromatide cohesion protein 3 |
| miR400 | Unigene7614_Longan-RNA:1:248:+ | 218 | 11.8854 | anaphase-promoting complex/cyclosome 8 |
|  | Unigene1548_Longan-RNA:1:629:+ | 462 | 29.364 | chloroplast bromodomain-containing protein |
|  | Unigene59111_Longan-RNA:1:539:+ | 217 | 28.6649 | dehydration-responsive protein-related |
|  |  | 218 | 11.1863 |  |
|  | Unigene7685_Longan-RNA:1:390:+ | 199 | 12.9342 | PAD4 |
|  | Unigene36879_Longan-RNA:1:237:+ | 113 | 17.4786 | ACRE 276-like protein |
|  |  | 114 | 15.7307 |  |
|  | Unigene54361_Longan-RNA:1:417:+ | 331 | 33.9085 | nucleotide-binding protein 1 |
|  | Unigene22478_Longan-RNA:1:173:+ | 124 | 33.5589 | Ketol-acid reductoisomerase, chloroplastic |
| miR477 | Unigene65404_Longan-RNA:1:941:+ | 666 | 11.8854 | chromatin remodeling complex subunit |
| miR1448,miR482 | Unigene39669_Longan-RNA:1:254:+ | 30 | 12.9342 |  |
| miR778 | Unigene41221_Longan-RNA:1:263:+ | 71 | 14.682 | nucleoporin 98 |
|  |  | 73 | 22.023 |  |
| miR778,miR2658 | Unigene59620_Longan-RNA:1:556:+ | 299 | 11.5359 | cyclophilin |
| miR780 | Unigene65144_Longan-RNA:1:910:+ | 336 | 68.8657 | similar to protein kinase |
|  |  | 337 | 30.0632 |  |
|  | Unigene7371_Longan-RNA:1:2463:+ | 2237 | 22.3726 | T2H3.2 |
|  | Unigene62118_Longan-RNA:1:669:+ | 134 | 31.1119 | Mo25 family protein |
|  | Unigene67271_Longan-RNA:1:1305:+ | 621 | 45.4443 | auxin response factor 2 |
|  |  | 622 | 98.5793 |  |
|  | Unigene52519_Longan-RNA:1:385:+ | 47 | 14.3324 | hydroxyacylglutathione hydrolase cytoplasmic-like |
|  | Unigene60720_Longan-RNA:1:601:+ | 244 | 10.4872 | ZIGA4 (ARF GAP-like zinc finger-containing protein ZiGA4); ARF GTPase activator/DNA binding / zinc ion binding |
|  | Unigene12047_Longan-RNA:1:945:+ | 689 | 21.3239 | At1g67840/F12A21_3 |
|  |  | 695 | 13.9829 |  |
|  | Unigene51163_Longan-RNA:1:364:+ | 327 | 13.6333 | cell division cycle protein |
|  |  | 330 | 27.6162 |  |
|  | Unigene60287_Longan-RNA:1:583:+ | 335 | 17.4786 | glycosyltransferase UGT95A1 |
|  |  | 361 | 90.8887 |  |
|  | Unigene38696_Longan-RNA:1:248:+ | 118 | 23.4213 | protein phosphatase 2A catalytic subunit |
|  | Unigene66996_Longan-RNA:1:1233:+ | 866 | 29.7136 | auxin efflux carrier family protein |
|  | Unigene61262_Longan-RNA:1:627:+ | 247 | 12.235 | GRAS family transcription factor |
|  |  | 248 | 15.0316 |  |
|  | Unigene14859_Longan-RNA:1:1461:+ | 1041 | 29.364 | glutamate dehydrogenase 2 |
| miR780,miR2628 | Unigene39676_Longan-RNA:1:254:+ | 140 | 10.8367 | kinase interacting protein-related |
|  |  | 145 | 15.3812 |  |
| miR815 | Unigene63029_Longan-RNA:1:722:+ | 454 | 19.576 | At4g14840 |
| miR830 | Unigene61578_Longan-RNA:1:641:+ | 186 | 25.5187 | serine-rich protein |
|  |  | 187 | 26.2179 |  |
|  | Unigene68876_Longan-RNA:1:3287:+ | 1710 | 22.7222 | 4-alpha-glucanotransferas |
|  | Unigene48367_Longan-RNA:1:327:+ | 156 | 17.4786 | Peptidase S26A, signal peptidase I; WD40-like |
| miR843 | Unigene46823_Longan-RNA:1:310:+ | 154 | 55.9315 | global transcription factor group |
| miR854 | Unigene5700_Longan-RNA:1:1501:+ | 94 | 350.6206 | kinase family protein |
|  |  | 95 | 199.6056 |  |
|  |  | 96 | 350.6206 |  |
|  |  | 97 | 199.6056 |  |
|  |  | 98 | 350.6206 |  |
|  |  | 99 | 199.6056 |  |
|  |  | 100 | 350.6206 |  |
|  |  | 101 | 199.6056 |  |
|  |  | 102 | 350.6206 |  |
|  |  | 103 | 199.6056 |  |
|  |  | 104 | 350.6206 |  |
|  |  | 106 | 212.5397 |  |
|  | Unigene9391_Longan-RNA:1:1547:+ | 1488 | 32.8598 | kinase family protein |
|  | Unigene9361_Longan-RNA:1:2135:+ | 1716 | 41.5991 | pentatricopeptide (PPR) repeat protein-like |
|  | Unigene10562_Longan-RNA:1:2555:+ | 145 | 350.6206 | At2g27090 |
|  |  | 146 | 199.6056 |  |
|  |  | 147 | 350.6206 |  |
|  |  | 148 | 199.6056 |  |
|  |  | 149 | 350.6206 |  |
|  |  | 150 | 199.6056 |  |
|  |  | 151 | 350.6206 |  |
|  |  | 152 | 198.2073 |  |
|  |  | 153 | 212.5397 |  |
|  |  | 154 | 42.6478 |  |
|  | Unigene798_Longan-RNA:1:1522:+ | 286 | 11.8854 | cysteine synthase |
|  |  | 287 | 10.4872 |  |
|  | Unigene8642_Longan-RNA:1:267:+ | 116 | 350.6206 | unnamed protein product |
|  |  | 117 | 199.6056 |  |
|  |  | 118 | 350.6206 |  |
|  |  | 119 | 199.6056 |  |
|  |  | 120 | 350.6206 |  |
|  |  | 121 | 199.6056 |  |
|  |  | 122 | 350.6206 |  |
|  |  | 123 | 198.2073 |  |
|  |  | 124 | 210.4423 |  |
|  |  | 125 | 55.2324 |  |
|  |  | 126 | 16.4299 |  |
|  | Unigene11296_Longan-RNA:1:1285:+ | 15 | 33.9085 | BAF06936.1\| Os01g0885200 |
|  | Unigene65640_Longan-RNA:1:971:+ | 161 | 14.682 | At3g52110 |
|  |  | 163 | 210.4423 |  |
|  |  | 164 | 55.2324 |  |
|  |  | 165 | 16.4299 |  |
|  |  | 166 | 17.129 |  |
|  | Unigene46886_Longan-RNA:1:310:+ | 70 | 14.682 | choline kinase GmCK2p-like protein |
|  | Unigene5323_Longan-RNA:1:1267:+ | 21 | 11.8854 | hypothetical protein |
|  |  | 22 | 14.682 |  |
|  |  | 24 | 11.5359 |  |
|  |  | 25 | 11.1863 |  |
|  | Unigene57560_Longan-RNA:1:491:+ | 376 | 11.8854 | BAF09797.1\| Os02g0708400 |
|  |  | 377 | 10.4872 |  |
|  | Unigene21710_Longan-RNA:1:170:+ | 125 | 14.682 | expressed protein |
|  | Unigene11587_Longan-RNA:1:2086:+ | 180 | 182.127 | Rieske iron-sulfur protein Tic55 |
|  |  | 181 | 43.3469 |  |
|  | Unigene11099_Longan-RNA:1:1469:+ | 159 | 350.6206 | leucine-rich repeat receptor-like protein kinase |
|  |  | 160 | 199.6056 |  |
|  |  | 161 | 350.6206 |  |
|  |  | 162 | 199.6056 |  |
|  |  | 163 | 350.6206 |  |
|  |  | 164 | 199.6056 |  |
|  |  | 165 | 350.6206 |  |
|  |  | 166 | 199.6056 |  |
|  |  | 167 | 350.6206 |  |
|  |  | 168 | 198.2073 |  |
|  |  | 169 | 212.5397 |  |
|  |  | 170 | 47.1922 |  |
|  | Unigene66610_Longan-RNA:1:1142:+ | 73 | 350.62 | myosin heavy chain-related |
|  |  | 74 | 199.6056 |  |
|  |  | 75 | 350.6206 |  |
|  |  | 76 | 183.1757 |  |
|  |  | 77 | 183.5252 |  |
|  |  | 78 | 32.8598 |  |
|  | Unigene63884_Longan-RNA:1:789:+ | 770 | 41.5991 | hydrolase, alpha/beta fold family protein |
|  | Unigene64281_Longan-RNA:1:823:+ | 106 | 212.5397 | hydrolase, alpha/beta fold family protein |
|  | Unigene6679_Longan-RNA:1:1747:+ | 51 | 185.6227 | peptidoglycan-binding LysM domain-containing protein |
|  |  | 52 | 181.7774 |  |
|  |  | 53 | 38.4529 |  |
|  | Unigene54257_Longan-RNA:1:415:+ | 80 | 55.2324 | At2g12550 |
|  |  | 81 | 16.4299 |  |
|  |  | 82 | 17.129 |  |
|  | Unigene12790_Longan-RNA:1:773:+ | 78 | 10.4872 | protein phosphatase 2C-like |
|  |  |  |  |  |
|  | Unigene54243_Longan-RNA:1:415:+ | 39 | 46.4931 | protein binding |
|  |  |  |  |  |
|  | Unigene66584_Longan-RNA:1:1136:+ | 1117 | 181.7774 | zinc finger (DHHC type) family protein |
|  | Unigene60542_Longan-RNA:1:593:+ | 68 | 17.4786 | f-box family protein |
|  | Unigene53153_Longan-RNA:1:395:+ | 366 | 14.682 | zinc finger protein |
|  |  | 367 | 185.6227 |  |
|  |  | 368 | 181.7774 |  |
|  |  | 369 | 38.4529 |  |
|  | Unigene12716_Longan-RNA:1:1404:+ | 1381 | 350.6206 | expressed protein |
|  |  | 1383 | 350.6206 |  |
|  | Unigene162_Longan-RNA:1:1689:+ | 340 | 11.5359 | NBS1 (NIJMEGEN BREAKAGE SYNDROME 1) |
|  | Unigene13862_Longan-RNA:1:1291:+ | 319 | 11.5359 | DNA binding with one finger 5 protein |
|  | Unigene37882_Longan-RNA:1:243:+ | 40 | 32.1606 | DNA binding protein, putative |
|  |  | 41 | 15.7307 |  |
|  |  | 43 | 20.2752 |  |
|  | Unigene65073_Longan-RNA:1:904:+ | 34 | 14.682 | short-chain dehydrogenase/reductase family protein |
|  |  | 35 | 199.6056 |  |
|  |  | 36 | 350.6206 |  |
|  |  | 37 | 183.1757 |  |
|  |  | 38 | 191.5654 |  |
|  |  | 39 | 46.1435 |  |
|  | Unigene64875_Longan-RNA:1:884:+ | 856 | 10.4872 | remorin family protein |
|  |  | 857 | 199.6056 |  |
|  |  | 858 | 350.6206 |  |
|  |  | 859 | 199.6056 |  |
|  |  | 860 | 350.6206 |  |
|  |  | 861 | 198.2073 |  |
|  |  | 862 | 212.5397 |  |
|  |  | 863 | 43.3469 |  |
|  | Unigene2047_Longan-RNA:1:603:+ | 466 | 16.0803 | self-pruning interacting protein 1 |
|  |  | 467 | 199.6056 |  |
|  |  | 468 | 350.6206 |  |
|  |  | 469 | 199.6056 |  |
|  |  | 470 | 350.6206 |  |
|  |  | 471 | 199.6056 |  |
|  |  | 472 | 350.6206 |  |
|  |  | 473 | 183.1757 |  |
|  |  | 474 | 189.468 |  |
|  |  | 475 | 32.1606 |  |
|  |  | 476 | 15.73 |  |
|  |  | 478 | 20.2752 |  |
|  | Unigene10853_Longan-RNA:1:1368:+ | 215 | 17.129 | similar to LHT1 (LYSI NEHISTIDINE TRANSPORTER 1); amino acid transmembrane transporter |
|  | Unigene64456_Longan-RNA:1:840:+ | 818 | 14.682 | CPuORF37 (Conserved peptide upstream open reading frame 37 |
|  |  | 819 | 198.2073 |  |
|  |  | 820 | 209.7431 |  |
|  |  | 821 | 41.5991 |  |
|  | Unigene68510_Longan-RNA:1:2007:+ | 41 | 350.6206 | ANTR2; inorganic phosphate transmembrane transporter/ organic anion transmembrane transporter |
|  |  | 42 | 185.6227 |  |
|  |  | 43 | 181.7774 |  |
|  |  | 44 | 39.5016 |  |
|  | Unigene50845_Longan-RNA:1:360:+ | 129 | 43.3469 | DND1-like protein |
|  | Unigene8538_Longan-RNA:1:328:+ | 119 | 350.6206 | Ubiquitin carboxyl-terminal hydrolase 7 |
|  |  | 120 | 185.6227 |  |
|  |  | 121 | 182.127 |  |
|  |  | 122 | 43.3469 |  |
|  | Unigene5981_Longan-RNA:1:613:+ | 589 | 14.682 | expressed protein |
|  | Unigene9773_Longan-RNA:1:2406:+ | 160 | 11.5359 | type-b response regulator |
|  | Unigene66117_Longan-RNA:1:1044:+ | 190 | 13.2837 | Zinc finger, C3HC4 type family protein |
|  | Unigene10081_Longan-RNA:1:974:+ | 103 | 183.5252 | At4g33740 |
|  |  | 104 | 34.9572 |  |
|  | Unigene2785_Longan-RNA:1:1759:+ | 87 | 10.4872 | HDL1 |
|  |  | 88 | 198.2073 |  |
|  |  | 89 | 210.4423 |  |
|  |  | 90 | 51.3871 |  |
|  |  | 91 | 14.682 |  |
|  |  | 92 | 14.3324 |  |
|  | Unigene6760_Longan-RNA:1:713:+ | 100 | 16.0803 | myosin heavy chain-related |
|  |  | 101 | 199.6056 |  |
|  |  | 102 | 350.6206 |  |
|  |  | 103 | 199.6056 |  |
|  |  | 104 | 350.6206 |  |
|  |  | 105 | 199.6056 |  |
|  |  | 106 | 350.6206 |  |
|  |  | 107 | 198.2073 |  |
|  |  | 108 | 210.7919 |  |
|  |  | 109 | 45.4443 |  |
|  | Unigene12685_Longan-RNA:1:2411:+ | 43 | 16.7795 | F5M15.15 |
|  |  | 47 | 75.8571 |  |
|  |  | 48 | 11.5359 |  |
|  | Unigene12650_Longan-RNA:1:579:+ | 301 | 14.682 | BAF10030.1\| Os02g0747500 |
|  |  | 302 | 14.3324 |  |
|  | Unigene3856_Longan-RNA:1:810:+ | 416 | 183.1757 | AP2 domain-containing transcription factor |
|  |  | 417 | 183.5252 |  |
|  | Unigene68225_Longan-RNA:1:1747:+ | 1724 | 350.6206 | heat shock protein binding protein |
|  |  | 1725 | 183.1757 |  |
|  |  | 1726 | 188.0697 |  |
|  |  | 1727 | 33.9085 |  |
|  | Unigene66815_Longan-RNA:1:1185:+ | 66 | 11.8854 | 1-phosphatidylinositol phosphodiesterase-related |
|  |  | 67 | 10.4872 |  |
|  | Unigene67825_Longan-RNA:1:1509:+ | 1398 | 191.5654 | similar to protein kinase |
|  |  | 1399 | 46.1435 |  |
|  | Unigene60612_Longan-RNA:1:597:+ | 497 | 16.7795 | kinase family protein |
|  |  | 498 | 24.1205 |  |
|  |  | 499 | 19.576 |  |
|  |  | 500 | 16.0803 |  |
|  |  | 501 | 183.1757 |  |
|  |  | 502 | 191.5654 |  |
|  |  | 503 | 46.1435 |  |
|  | Unigene59282_Longan-RNA:1:544:+ | 150 | 17.129 | cellulose synthase CesA1 |
|  | Unigene2367_Longan-RNA:1:572:+ | 546 | 350.6206 | phosphoesterase |
|  |  | 547 | 199.6056 |  |
|  |  | 548 | 350.6206 |  |
|  |  | 549 | 183.1757 |  |
|  |  | 550 | 183.5252 |  |
|  |  | 551 | 34.9572 |  |
|  | Unigene57949_Longan-RNA:1:502:+ | 69 | 32.16 | At2g42110 |
|  | Unigene55980_Longan-RNA:1:452:+ | 15 | 14.3324 | cytochrome P-450 |
|  | Unigene63472_Longan-RNA:1:756:+ | 269 | 75.8571 | formin homology 2 domain-containing protein / FH2 domain-containing protein |
|  |  | 270 | 11.5359 |  |
|  |  | 271 | 11.8854 |  |
|  | Unigene14408_Longan-RNA:1:4104:+ | 3661 | 16.0803 | cellulose synthase |
|  |  | 3662 | 199.6056 |  |
|  |  | 3663 | 350.6206 |  |
|  |  | 3664 | 198.2073 |  |
|  |  | 3665 | 212.5397 |  |
|  |  | 3666 | 42.6478 |  |
|  | Unigene67583_Longan-RNA:1:1414:+ | 1384 | 183.1757 | similar to transcription factor-related |
|  |  | 1385 | 188.0697 |  |
|  |  | 1386 | 33.9085 |  |
|  | Unigene58161_Longan-RNA:1:508:+ | 314 | 26.2179 | similar to Callose synthase 3 |
|  |  | 315 | 19.2265 |  |
|  | Unigene14084_Longan-RNA:1:1896:+ | 1632 | 12.235 | CYP77A5P; catalytic |
|  | Unigene14168_Longan-RNA:1:876:+ | 44 | 11.5359 | calmodulin-binding family protein |
|  |  | 45 | 51.3871 |  |
|  |  | 46 | 14.682 |  |
|  |  | 47 | 14.3324 |  |
|  | Unigene63486_Longan-RNA:1:757:+ | 87 | 14.682 | SYNC1 protein |
|  |  | 88 | 14.3324 |  |
|  | Unigene50832_Longan-RNA:1:360:+ | 338 | 16.0803 | alpha-1,4-glucan phosphorylase L isozyme |
|  |  | 339 | 198.2073 |  |
|  |  | 340 | 209.7431 |  |
|  |  | 341 | 41.5991 |  |
|  | Unigene45239_Longan-RNA:1:294:+ | 54 | 11.8854 | AGP22 (ARABINOGALACTAN PROTEIN 22) |
|  | Unigene56723_Longan-RNA:1:470:+ | 446 | 350.6206 | Probable ADP-ribosylation factor GTPase-activating protein AGD11 |
|  |  | 447 | 199.6056 |  |
|  |  | 448 | 350.6206 |  |
|  |  | 449 | 198.2073 |  |
|  |  | 450 | 209.7431 |  |
|  |  | 451 | 41.5991 |  |
|  | Unigene67185_Longan-RNA:1:1277:+ | 282 | 11.8854 | glutamyl tRNA Reductase |
|  |  | 283 | 14.682 |  |
|  |  | 285 | 11.5359 |  |
|  | Unigene62429_Longan-RNA:1:687:+ | 35 | 350.6206 | AT1G79090 |
|  |  | 36 | 198.2073 |  |
|  |  | 37 | 210.7919 |  |
|  |  | 38 | 46.4931 |  |
|  | Unigene10685_Longan-RNA:1:695:+ | 151 | 12.235 | SMA9 |
|  |  | 154 | 12.235 |  |
|  | Unigene8462_Longan-RNA:1:920:+ | 108 | 350.6206 | zinc finger (C3HC4-type RING finger) family protein |
|  |  | 109 | 198.2073 |  |
|  |  | 110 | 210.7919 |  |
|  |  | 111 | 46.4931 |  |
|  | Unigene12998_Longan-RNA:1:3615:+ | 239 | 41.5991 | autoinhibited calcium ATPase |
|  | Unigene68095_Longan-RNA:1:1640:+ | 52 | 14.682 | phototropic-responsive NPH3 family protein |
|  |  | 53 | 199.6056 |  |
|  |  | 54 | 350.6206 |  |
|  |  | 55 | 183.1757 |  |
|  |  | 56 | 183.5252 |  |
|  |  | 57 | 34.258 |  |
|  | Unigene11084_Longan-RNA:1:470:+ | 166 | 11.8854 | RING-H2 finger protein ATL5C |
|  |  | 167 | 14.682 |  |
|  |  | 169 | 11.5359 |  |
|  | Unigene67975_Longan-RNA:1:1578:+ | 1357 | 16.7795 | peptidoglycan-binding LysM domain-containing protein |
|  |  | 1388 | 14.682 |  |
|  |  | 1389 | 185.6227 |  |
|  |  | 1390 | 181.7774 |  |
|  |  | 1391 | 38.4529 |  |
|  | Unigene5408_Longan-RNA:1:880:+ | 87 | 16.0803 | Sodium Bile acid symporter family protein, expressed |
|  |  | 88 | 199.6056 |  |
|  |  | 89 | 350.6206 |  |
|  |  | 90 | 199.6056 |  |
|  |  | 91 | 350.6206 |  |
|  |  | 92 | 183.1757 |  |
|  |  | 93 | 183.5252 |  |
|  |  | 94 | 34.258 |  |
|  | Unigene12006_Longan-RNA:1:2184:+ | 197 | 46.4931 | At1g68060/T23K23_9 |
|  |  | 200 | 75.8571 |  |
|  |  | 201 | 11.5359 |  |
|  | Unigene15156_Longan-RNA:1:1179:+ | 1086 | 350.6206 | MEE47 (maternal effect embryo arrest 47); transcription factor |
|  |  | 1087 | 199.6056 |  |
|  |  | 1088 | 350.6206 |  |
|  |  | 1089 | 199.6056 |  |
|  |  | 1090 | 350.6206 |  |
|  |  | 1091 | 183.1757 |  |
|  |  | 1092 | 183.5252 |  |
|  |  | 1093 | 32.8598 |  |
|  | Unigene9585_Longan-RNA:1:842:+ | 159 | 350.6206 | zinc ion binding protein |
|  |  | 160 | 199.6056 |  |
|  |  | 161 | 350.6206 |  |
|  |  | 162 | 198.2073 |  |
|  |  | 163 | 212.5397 |  |
|  |  | 164 | 43.3469 |  |
|  | Unigene9420_Longan-RNA:1:949:+ | 162 | 350.6206 | ZFP2 (ZINC FINGER PROTEIN 2); nucleic acid binding / transcription factor/ zinc ion binding |
|  |  | 170 | 350.6206 |  |
|  |  | 174 | 350.6206 |  |
|  |  | 180 | 350.6206 |  |
|  |  | 182 | 350.6206 |  |
|  |  | 182 | 350.6206 |  |
|  |  | 194 | 350.6206 |  |
|  | Unigene10942_Longan-RNA:1:1108:+ | 157 | 90.5391 | Multicopper oxidase, type 1 |
|  |  | 158 | 350.6206 |  |
|  |  | 159 | 198.2073 |  |
|  |  | 160 | 210.4423 |  |
|  |  | 161 | 55.2324 |  |
|  |  | 162 | 18.1777 |  |
|  |  | 163 | 18.5273 |  |
|  | Unigene13974_Longan-RNA:1:966:+ | 74 | 11.8854 | CBL-interacting protein kinase 15 |
|  |  | 165 | 11.8854 |  |
|  |  | 166 | 14.682 |  |
|  |  | 168 | 11.8854 |  |
|  |  | 169 | 10.8367 |  |
|  | Unigene1601_Longan-RNA:1:2469:+ | 81 | 16.0803 | trichome birefringence |
|  |  | 82 | 198.2073 |  |
|  |  | 83 | 210.4423 |  |
|  |  | 84 | 55.2324 |  |
|  |  | 85 | 19.2265 |  |
|  |  | 86 | 15.7307 |  |
|  | Unigene1643_Longan-RNA:1:632:+ | 87 | 199.6056 | f-box family protein |
|  |  | 88 | 350.6206 |  |
|  |  | 89 | 199.6056 |  |
|  |  | 90 | 350.6206 |  |
|  |  | 91 | 199.6056 |  |
|  |  | 92 | 350.6206 |  |
|  |  | 93 | 199.6056 |  |
|  |  | 94 | 350.6206 |  |
|  |  | 95 | 199.6056 |  |
|  |  | 96 | 350.6206 |  |
|  |  | 97 | 198.2073 |  |
|  |  | 98 | 212.5397 |  |
|  | Unigene28808_Longan-RNA:1:198:+ | 180 | 23.4213 | GRAS family transcription factor |
|  | Unigene4582_Longan-RNA:1:501:+ | 89 | 350.6206 | BAF23002.1\| Os08g0169700 |
|  |  | 90 | 199.6056 |  |
|  |  | 91 | 350.6206 |  |
|  |  | 92 | 199.6056 |  |
|  |  | 93 | 350.6206 |  |
|  |  | 94 | 199.6056 |  |
|  |  | 95 | 350.6206 |  |
|  |  | 96 | 199.6056 |  |
|  |  | 97 | 350.6206 |  |
|  |  | 98 | 198.2073 | 1 |
|  |  | 99 | 210.4423 | 1 |
|  |  | 100 | 55.2324 |  |
|  | Unigene14352_Longan-RNA:1:503:+ | 407 | 14.682 | universal stress protein (USP) family protein / early nodulin ENOD18 family protein |
|  |  | 408 | 199.6056 |  |
|  |  | 409 | 350.6206 |  |
|  |  | 410 | 198.2073 |  |
|  |  | 411 | 210.7919 |  |
|  |  | 412 | 49.2896 |  |
|  | Unigene7765_Longan-RNA:1:723:+ | 106 | 350.6206 | f-box family protein |
|  |  | 107 | 199.6056 |  |
|  |  | 108 | 350.6206 |  |
|  |  | 109 | 199.6056 |  |
|  |  | 110 | 350.6206 |  |
|  |  | 111 | 183.1757 |  |
|  |  | 112 | 188.0697 |  |
|  |  | 113 | 33.9085 |  |
|  | Unigene2307_Longan-RNA:1:464:+ | 80 | 90.5391 | At3g09050 |
|  |  | 81 | 350.6206 |  |
|  |  | 82 | 199.6056 |  |
|  |  | 83 | 350.6206 |  |
|  |  | 84 | 199.6056 |  |
|  |  | 85 | 350.6206 |  |
|  |  | 86 | 199.6056 |  |
|  |  | 87 | 350.6206 |  |
|  |  | 88 | 198.2073 |  |
|  |  | 89 | 210.7919 |  |
|  |  | 90 | 49.2896 |  |
|  | Unigene13107_Longan-RNA:1:1469:+ | 73 | 18.1777 | BAF21277.1\| Os07g0275300 |
|  |  | 74 | 18.5273 |  |
|  | Unigene64388_Longan-RNA:1:833:+ | 807 | 210.7919 | At2g25605 |
|  |  | 808 | 46.4931 | 1 |
|  |  | 811 | 75.158 | 1 |
| miR854,miR1436 | Unigene54544_Longan-RNA:1:421:+ | 47 | 19.576 | C2 |
|  |  | 48 | 94.3844 |  |
|  |  | 49 | 163.5997 |  |
|  |  | 50 | 16.4299 |  |
|  |  | 161 | 18.1777 |  |
| miR855 | Unigene13413_Longan-RNA:1:1219:+ | 1155 | 26.917 | BAF22822.1\| Os08g0128000 |
|  |  | 1156 | 27.6162 |  |
|  |  | 1157 | 117.1066 |  |
| miR860 | Unigene68458_Longan-RNA:1:1949:+ | 1678 | 13.2837 | Unknown protein |
|  | Unigene61541_Longan-RNA:1:639:+ | 279 | 14.3324 | zinc finger (C3HC4-type RING finger) family protein |
|  | Unigene152_Longan-RNA:1:499:+ | 472 | 42.6478 | Dihydrodipicolinate reductase 2, chloroplastic OS |
|  |  | 473 | 17.129 |  |
| miR861 | Unigene62636_Longan-RNA:1:699:+ | 386 | 10.1376 | arginine-tRNA-protein transferase 1 |
|  | Unigene58942_Longan-RNA:1:533:+ | 95 | 15.0316 | Ycf1 |
|  | Unigene65694_Longan-RNA:1:980:+ | 182 | 10.1376 | zinc finger family protein |
| miR862 | Unigene5723_Longan-RNA:1:1352:+ | 1323 | 26.917 | beta-glucosidase |
|  | Unigene59724_Longan-RNA:1:560:+ | 112 | 27.2666 | AF08824.1\| Os02g0504100 |
|  |  | 113 | 10.4872 |  |
| miR866 | Unigene62623_Longan-RNA:1:698:+ | 323 | 20.2752 | unnamed protein product |
|  |  | 327 | 11.5359 |  |
| miR866,miR267 | Unigene58240_Longan-RNA:1:511:+ | 429 | 11.5359 | amine oxidase/ polyamine oxidas |
|  |  | 490 | 23.4213 |  |
|  |  | 491 | 50.6879 |  |
| miR867 | Unigene66452_Longan-RNA:1:1108:+ | 319 | 16.7795 | F27J15.26 |
| miR870 | Unigene62794_Longan-RNA:1:709:+ | 499 | 11.5359 | putrescine-binding periplasmic protein-related |
|  | Unigene58165_Longan-RNA:1:508:+ | 31 | 16.4299 | Vesicle transport v-SNARE; t-snare |
|  | Unigene61038_Longan-RNA:1:616:+ | 299 | 18.1777 | casein kinase I-like |
| miR870,miR1512 | Unigene67770_Longan-RNA:1:1484:+ | 367 | 14.3324 | NAK-type protein kinase |
|  |  | 741 | 40.8999 |  |
|  |  | 742 | 12.584 |  |
| miR870,miR2641 | Unigene67381_Longan-RNA:1:1340:+ | 1113 | 24.1205 | At2g16090/F7H1.11 |
|  |  | 1271 | 12.5846 |  |
| miR870,miR1069 | Unigene60875_Longan-RNA:1:609:+ | 318 | 11.8854 | TATA binding protein associated factor |
|  |  | 541 | 11.5359 |  |
|  |  | 542 | 16.4299 |  |
| miR916 | Unigene23966_Longan-RNA:1:179:+ | 44 | 12.584 | GRAS family transcription factor |
|  | Unigene7352_Longan-RNA:1:1741:+ | 1556 | 11.5359 | binding |
| miR952 | Unigene39145_Longan-RNA:1:250:+ | 140 | 15.0316 | bile acid:Na+ symporter family protein |
| miR1027 | Unigene19078_Longan-RNA:1:160:+ | 31 | 14.3324 | hypothetical protein isoform 2 |
|  | Unigene67267_Longan-RNA:1:1304:+ | 294 | 10.1376 | DEAD-box ATP-dependent RNA helicase 13 |
| miR1027,miR2673 | Unigene57746_Longan-RNA:1:496:+ | 301 | 22.7222 | binding |
|  |  | 302 | 111.1639 |  |
| miR1036 | Unigene64911_Longan-RNA:1:886:+ | 403 | 11.8854 | early flowering 3 |
| miR1041 | Unigene43698_Longan-RNA:1:281:+ | 118 | 23.0717 | AT3g53630/F4P12_330 |
|  | Unigene67604_Longan-RNA:1:1419:+ | 492 | 13.283 | Nuclear factor related to kappa-B-binding protein , related |
| miR1041,miR2644 | Unigene9048_Longan-RNA:1:632:+ | 572 | 12.584 | ACP reductase |
| miR1046 | Unigene68248_Longan-RNA:1:1763:+ | 1576 | 24.8196 | ARK3 (ARMADILLO REPEAT KINESIN 3); ATP binding / binding / microtubule motor |
|  | Unigene2291_Longan-RNA:1:680:+ | 112 | 29.0145 | AT5G09960-like protein |
|  | Unigene3221_Longan-RNA:1:4075:+ | 4019 | 36.3555 | BAF21336.1\| Os07g0300200 |
|  | Unigene56964_Longan-RNA:1:475:+ | 345 | 30.4128 | transcription factor |
|  |  | 354 | 26.2179 |  |
|  | Unigene67674_Longan-RNA:1:1445:+ | 709 | 13.6333 | mitotic phosphoprotein N' end (MPPN)family protein |
|  | Unigene60747_Longan-RNA:1:603:+ | 513 | 10.4872 | BAF22216.1\| Os07g0620600 |
|  | Unigene13899_Longan-RNA:1:1363:+ | 641 | 27.2666 | protein phosphatase-2C |
|  |  | 642 | 25.8683 |  |
|  | Unigene53959_Longan-RNA:1:410:+ | 334 | 15.7307 | LysM type receptor kinase |
|  |  | 335 | 16.0803 |  |
|  | Unigene65893_Longan-RNA:1:1009:+ | 289 | 25.1692 | heat-intolerant 1 |
|  |  | 290 | 16.4299 |  |
|  | Unigene12736_Longan-RNA:1:1720:+ | 1304 | 16.4299 | ABC transporter like protein |
|  | Unigene31683_Longan-RNA:1:210:+ | 184 | 12.9342 | endo-alpha-1,4-glucanase |
|  |  | 185 | 29.364 |  |
|  | Unigene31452_Longan-RNA:1:209:+ | 63 | 19.2265 | AT3G01090 |
|  | Unigene65821_Longan-RNA:1:995:+ | 403 | 83.8973 | At2g19940/F6F22.3 |
|  |  | 404 | 19.576 |  |
|  | Unigene57009_Longan-RNA:1:477:+ | 444 | 17.828 | glycinamide ribonucleotide synthetase |
|  | Unigene7697_Longan-RNA:1:339:+ | 42 | 11.1863 | PAD4 |
|  | Unigene64424_Longan-RNA:1:837:+ | 594 | 15.3812 | iaa-amino acid hydrolase 4 |
|  | Unigene36548_Longan-RNA:1:235:+ | 147 | 74.4588 | similar to ATPDR3/PDR3 (PLEIOTROPIC DRUG RESISTANCE 3); ATPase, coupled to transmembrane movement of substances |
|  |  | 148 | 106.6194 |  |
|  | Unigene19807_Longan-RNA:1:163:+ | 125 | 81.4503 | rhamnose synthase |
|  |  | 126 | 12.235 |  |
|  | Unigene65954_Longan-RNA:1:1019:+ | 511 | 13.9829 | enhancer of polycomb-like protein |
|  | Unigene67331_Longan-RNA:1:1325:+ | 207 | 61.1751 | At1g32160/F3C3_6 |
|  |  | 208 | 47.1922 |  |
|  | Unigene58603_Longan-RNA:1:522:+ | 236 | 21.6735 | F5J5.4 |
| miR1048,miR3440 | Unigene5669_Longan-RNA:1:586:+ | 219 | 46.1435 | osmotin-like protein I |
|  |  | 222 | 93.6853 |  |
|  |  | 233 | 92.6366 |  |
|  |  | 564 | 44.0461 |  |
|  |  | 565 | 50.3384 |  |
| miR1048,miR1510 | Unigene61378_Longan-RNA:1:631:+ | 574 | 11.1863 | RAB7D |
| miR1061 | Unigene54657_Longan-RNA:1:423:+ | 321 | 22.7222 | BAF08824.1\| Os02g0504100 |
|  |  | 322 | 12.5846 |  |
|  | Unigene52547_Longan-RNA:1:385:+ | 293 | 27.9658 | heat shock protein 82 |
|  | Unigene65876_Longan-RNA:1:1006:+ | 849 | 53.1349 | xyloglucan endotransglucosylase/hydrolase 5 |
|  | Unigene56542_Longan-RNA:1:465:+ | 277 | 12.9342 | 26S proteasome subunit S3 |
| miR1061,miR854 | Unigene59844_Longan-RNA:1:565:+ | 444 | 45.4443 | coatomer delta subunit |
|  |  | 445 | 17.4786 |  |
|  |  | 521 | 22.7222 |  |
| miR1074 | Unigene62493_Longan-RNA:1:691:+ | 377 | 27.6162 | G/HBF-1 |
| miR1081 | Unigene60626_Longan-RNA:1:597:+ | 340 | 29.0145 | AKIN gamma |
|  |  | 341 | 29.364 |  |
|  | Unigene66094_Longan-RNA:1:1041:+ | 327 | 89.1408 | photoperiod responsive protein |
|  | Unigene54623_Longan-RNA:1:423:+ | 22 | 29.0145 | transcription factor group |
|  |  | 23 | 25.1692 |  |
|  | Unigene57435_Longan-RNA:1:488:+ | 461 | 53.8341 | (Sanskrit for 'bright');poly(ADP-ribose) glycohydrolase |
|  |  | 462 | 212.5397 |  |
|  | Unigene67188_Longan-RNA:1:1279:+ | 135 | 13.2837 | predicted protein |
|  | Unigene66879_Longan-RNA:1:1203:+ | 789 | 10.1376 | UDP-galactose/glucose pyrophosphorylase |
|  |  | 790 | 23.7709 |  |
| miR1081,miR1535 | Unigene44167_Longan-RNA:1:285:+ | 218 | 13.9829 | EFH68346.1\| T25K16.10 |
|  |  | 235 | 11.5359 |  |
|  | Unigene59833_Longan-RNA:1:565:+ | 459 | 33.5589 | G protein gamma subunit 2 |
| miR1102 | Unigene58041_Longan-RNA:1:504:+ | 212 | 12.235 | T23E23.25 |
| miR1102 | Unigene16706_Longan-RNA:1:152:+ | 42 | 27.6162 | DNA helicase, TBP-interacting protein |
|  |  | 45 | 47.8914 |  |
|  |  | 46 | 13.9829 |  |
|  |  | 47 | 28.3153 |  |
|  |  | 48 | 12.9342 |  |
|  |  | 49 | 13.6333 |  |
| miR1122 | Unigene68174_Longan-RNA:1:1703:+ | 1551 | 16.0803 | At3g60850 |
|  | Unigene51514_Longan-RNA:1:369:+ | 95 | 26.917 | RNA recognition motif (RRM)-containing protein |
|  | Unigene7212_Longan-RNA:1:555:+ | 369 | 16.0803 | protein binding / ubiquitin-protein ligase/ zinc ion binding |
| miR1127 | Unigene146_Longan-RNA:1:462:+ | 205 | 27.9658 | Retinoblastoma-related protein |
| miR1132 | Unigene45523_Longan-RNA:1:297:+ | 237 | 22.3726 | EMB2737 |
|  |  | 238 | 10.1376 |  |
|  | Unigene2557_Longan-RNA:1:972:+ | 737 | 18.8769 | predicted protein |
|  |  | 740 | 42.6478 |  |
|  | Unigene62381_Longan-RNA:1:684:+ | 396 | 38.1033 | leucine-rich repeat family protein |
|  |  | 397 | 31.4615 |  |
| miR1137 | Unigene61023_Longan-RNA:1:616:+ | 433 | 53.1349 | LEM3 (ligand-effect modulator 3) family protein |
|  |  | 434 | 20.9743 |  |
|  | Unigene68525_Longan-RNA:1:2028:+ | 935 | 14.682 | circadian clock-associated FKF1 |
|  | Unigene56017_Longan-RNA:1:453:+ | 298 | 11.1863 | SAUR family protein |
|  |  | 299 | 233.1645 |  |
|  |  | 308 | 30.7623 |  |
|  |  | 309 | 10.8367 |  |
|  | Unigene54803_Longan-RNA:1:426:+ | 398 | 11.8854 | Serine/threonine protein phosphatase 2A 55 kDa regulatory subunit B beta isoform; |
|  | Unigene52482_Longan-RNA:1:384:+ | 98 | 102.4246 | BAF06791.1\| Os01g0861700 |
|  |  | 99 | 37.753 |  |
|  | Unigene45656_Longan-RNA:1:298:+ | 271 | 18.8769 | AT3G25530 |
|  | Unigene65827_Longan-RNA:1:997:+ | 963 | 18.8769 | receptor kinase |
|  |  | 964 | 10.4872 |  |
|  | Unigene66788_Longan-RNA:1:1178:+ | 428 | 15.0316 | Ser/Thr specific protein phosphatase 2A B regulatory subunit beta isoform |
| miR1147,miR158 | Unigene5294_Longan-RNA:1:423:+ | 225 | 29.0145 | enoyl-CoA hydratase |
|  |  | 226 | 22.7222 |  |
| miR1153 | Unigene62730_Longan-RNA:1:705:+ | 509 | 16.0803 | APUM12 (ARABIDOPSIS PUMILIO 12); RNA binding / binding |
|  |  | 510 | 111.1639 |  |
|  | Unigene12175_Longan-RNA:1:1247:+ | 295 | 15.7307 | flavin-dependent monooxygenase 1 |
| miR1154 | Unigene12456_Longan-RNA:1:1053:+ | 836 | 62.9229 | somatic embryogenesis receptor kinase |
|  |  | 837 | 13.6333 |  |
| miR1169 | Unigene50800_Longan-RNA:1:359:+ | 125 | 15.3812 | trehalase |
| miR1170 | Unigene55733_Longan-RNA:1:446:+ | 49 | 14.3324 | C2 domain-containing protein |
|  | Unigene37263_Longan-RNA:1:240:+ | 106 | 42.6478 | protein-tyrosine-phosphatase-like protein |
| miR1211 | Unigene22374_Longan-RNA:1:173:+ | 38 | 20.2752 | Fas-associated factor 1 |
|  |  | 39 | 10.4872 |  |
| miR1211,miR854 | Unigene13687_Longan-RNA:1:1083:+ | 147 | 210.7919 | AP2 domain class transcription factor |
|  |  | 148 | 49.2896 |  |
| miR1211,miR854 | Unigene64399_Longan-RNA:1:834:+ | 260 | 16.0803 | DNA-binding protein-related |
|  |  | 261 | 183.1757 |  |
|  |  | 262 | 183.5252 |  |
|  |  | 263 | 32.8598 |  |
| miR1211,miR1917,miR835 | Unigene62028_Longan-RNA:1:664:+ | 49 | 11.8854 | protein binding protein |
|  |  | 55 | 12.9342 |  |
| miR1217 | Unigene46821_Longan-RNA:1:310:+ | 184 | 17.129 | Pyruvate kinase |
| miR1217,miR2643 | Unigene48610_Longan-RNA:1:330:+ | 257 | 40.8999 | H0814G11.12 |
|  |  | 258 | 12.9342 |  |
|  |  | 261 | 30.7623 |  |
|  |  | 268 | 20.6247 |  |
|  |  | 271 | 17.4786 |  |
| miR1320 | Unigene29142_Longan-RNA:1:199:+ | 115 | 15.7307 | AT3G26720 |
|  | Unigene65177_Longan-RNA:1:914:+ | 479 | 31.1119 | NAD-dependent epimerase/dehydratase family protein |
|  | Unigene944_Longan-RNA:1:707:+ | 586 | 13.2837 | Putative serine /threonine kinase similar to NAK |
|  | Unigene54650_Longan-RNA:1:423:+ | 182 | 41.9486 | similar to GTP-binding family protein |
|  |  | 183 | 10.4872 |  |
| miR1424 | Unigene53967_Longan-RNA:1:410:+ | 298 | 21.3239 | At2g40095 |
| miR1426 | Unigene15194_Longan-RNA:1:1332:+ | 405 | 16.0803 | U-box domain-containing protein 24 |
|  |  | 406 | 31.811 |  |
|  | Unigene59489_Longan-RNA:1:552:+ | 476 | 39.8512 | Hypothetical protein |
|  | Unigene16857_Longan-RNA:1:153:+ | 95 | 11.8854 | econdary cell wall-related glycosyltransferase family 47 |
|  |  | 96 | 11.8854 |  |
|  | Unigene63589_Longan-RNA:1:766:+ | 441 | 13.6333 | kinesin-like protein |
|  | Unigene2435_Longan-RNA:1:789:+ | 511 | 13.6333 | chloroplast protein translocon component Tic40 precursor |
|  | Unigene67923_Longan-RNA:1:1557:+ | 1423 | 170.591 | BAF22314.1\| Os07g0637200 |
|  | Unigene10203_Longan-RNA:1:1093:+ | 413 | 15.0316 | BAF22964.1\| Os08g0161500 |
|  | Unigene50755_Longan-RNA:1:359:+ | 261 | 19.2265 | quinone oxidoreductase |
|  |  | 262 | 18.1777 |  |
|  | Unigene60113_Longan-RNA:1:575:+ | 426 | 141.9262 | ferredoxin family protein, expressed |
|  |  | 427 | 41.5991 |  |
|  | Unigene67756_Longan-RNA:1:1478:+ | 1428 | 14.682 | armadillo repeat-containing protein |
|  |  | 1429 | 25.5187 |  |
|  | Unigene55366_Longan-RNA:1:438:+ | 335 | 16.4299 | esterase/lipase/thioesterase family protein |
|  | Unigene60864_Longan-RNA:1:609:+ | 454 | 15.3812 | DNAJ chaperone C-terminal domain-containing protein |
|  | Unigene64498_Longan-RNA:1:844:+ | 729 | 10.8367 | serine/threonine protein kinase |
|  |  | 730 | 18.5273 |  |
|  |  | 731 | 15.0316 |  |
| miR1426,miR2667 | Unigene68559_Longan-RNA:1:2070:+ | 1257 | 12.9342 | plastid alpha-amylase |
|  |  | 1551 | 10.4872 |  |
| miR1426,miR1508 | Unigene59141_Longan-RNA:1:540:+ | 22 | 18.1777 | CTP synthase-like protein |
|  |  | 274 | 12.235 |  |
| miR1426,miR1320 | Unigene46579_Longan-RNA:1:307:+ | 189 | 11.5359 | endonuclease/exonuclease/phosphatase family protein |
|  |  | 190 | 10.8367 |  |
|  |  | 196 | 24.1205 |  |
|  |  | 197 | 18.1777 |  |
| miR1436 | Unigene31603_Longan-RNA:1:210:+ | 53 | 22.3726 | protein binding protein |
|  |  | 54 | 88.0921 |  |
|  |  | 55 | 18.8769 |  |
|  | Unigene65484_Longan-RNA:1:951:+ | 872 | 34.258 | EMB2753 |
| miR1438 | Unigene4838_Longan-RNA:1:2993:+ | 1757 | 13.6333 | zinc finger protein-like |
|  | Unigene52549_Longan-RNA:1:385:+ | 179 | 13.6333 | peroxidase |
|  |  | 187 | 33.5589 |  |
|  |  | 188 | 16.4299 |  |
|  | Unigene60620_Longan-RNA:1:597:+ | 161 | 12.235 | isocitrate dehydrogenase (NAD+) |
|  | Unigene66995_Longan-RNA:1:1233:+ | 820 | 29.7136 | BAF28273.1\| Os11g0484300 |
|  | Unigene61482_Longan-RNA:1:636:+ | 363 | 31.4615 | At5g18420 |
|  | Unigene61182_Longan-RNA:1:623:+ | 124 | 17.4786 | aspartyl protease family protein |
|  | Unigene45794_Longan-RNA:1:300:+ | 30 | 29.0145 | transducin family protein |
|  |  | 31 | 27.6162 |  |
|  | Unigene58298_Longan-RNA:1:512:+ | 290 | 10.8367 | BAF25730.1\| Os09g0541700 |
|  | Unigene8496_Longan-RNA:1:3140:+ | 343 | 81.1007 | ATPase subunit 1 |
| miR1438,miR1525 | Unigene68623_Longan-RNA:1:2207:+ | 2104 | 26.2179 | Concanavalin A-like lectin/glucanase; U2 auxiliary factor small subunit |
| miR1438,miR1525 | Unigene51554_Longan-RNA:1:370:+ | 98 | 51.3871 | forkhead-associated domain-containing protein / FHA domain-containing protein |
|  |  | 99 | 34.258 |  |
| miR1442 | Unigene53240_Longan-RNA:1:397:+ | 253 | 26.5675 | At1g27150/T7N9_21 |
|  | Unigene67596_Longan-RNA:1:1416:+ | 82 | 12.235 | importin beta-2 subunit family protein |
|  | Unigene55351_Longan-RNA:1:438:+ | 98 | 16.4299 | At2g26470 |
|  | Unigene63155_Longan-RNA:1:732:+ | 261 | 10.4872 | swap (Suppressor-of-White-APricot)/surp domain-containing protein |
|  | Unigene50256_Longan-RNA:1:351:+ | 262 | 10.1376 | cullin 4 |
|  | Unigene62466_Longan-RNA:1:689: | 502 | 11.5359 | Vesicle-associated membrane protein 724 |
|  | Unigene50903_Longan-RNA:1:361:+ | 179 | 15.73 | PAF1 complex component |
|  | Unigene58035_Longan-RNA:1:504:+ | 335 | 35.6563 | Universal stress protein family protein |
|  | Unigene46897_Longan-RNA:1:310:+ | 273 | 142.6253 | peptidylprolyl cis-trans isomerase-like protein |
|  |  | 274 | 105.9203 |  |
|  |  | 275 | 15.0316 |  |
|  |  | 276 | 15.7307 |  |
|  | Unigene68230_Longan-RNA:1:1749:+ | 1481 | 15.3812 | cullin-associated and neddylation dissociated, hemivenata |
| miR1438,miR1442 | Unigene63244_Longan-RNA:1:739:+ | 569 | 30.0632 | expressed protein |
|  |  | 570 | 22.3726 |  |
|  |  | 571 | 22.3726 |  |
| miR1442,miR2673 | Unigene61168_Longan-RNA:1:623:+ | 120 | 80.4015 | hypothetical protein |
| miR1444,miR867 | Unigene66496_Longan-RNA:1:1116:+ | 655 | 12.5846 | ATP binding / microtubule motor |
|  |  | 694 | 16.7795 |  |
|  |  | 695 | 11.5359 |  |
| miR1448 | Unigene50560_Longan-RNA:1:356:+ | 269 | 19.2265 | trehalose synthase/phosphatase-like protein |
| miR1508 | Unigene67777_Longan-RNA:1:1489:+ | 1255 | 12.5846 | At5g67620 |
|  | Unigene2703_Longan-RNA:1:2018:+ | 958 | 10.1376 | SET domain protein |
|  | Unigene68317_Longan-RNA:1:1816:+ | 1648 | 45.7939 | CDPK-related protein kinase |
|  |  | 1649 | 27.9658 |  |
|  | Unigene64061_Longan-RNA:1:804:+ | 689 | 15.7307 | similar to leucine-rich repeat protein-related |
| miR1508,miR5170 | Unigene2490_Longan-RNA:1:1566:+ | 1432 | 13.6333 | P-P-bond-hydrolysis-driven protein transmembrane transporter/ catalytic |
|  |  | 1451 | 11.1863 |  |
| miR1508,miR854 | Unigene9102_Longan-RNA:1:946:+ | 203 | 350.6206 | ABC transporter G family member 34 |
|  |  | 204 | 198.2073 |  |
|  |  | 205 | 210.4423 |  |
|  |  | 206 | 55.2324 |  |
|  |  | 207 | 18.1777 |  |
|  |  | 208 | 18.5273 |  |
| miR1512 | Unigene8742_Longan-RNA:1:1065:+ | 712 | 31.811 | zinc ion binding |
|  |  | 715 | 27.6162 |  |
|  | Unigene60281_Longan-RNA:1:582:+ | 484 | 13.6333 | decapping protein 2-like |
|  | Unigene58882_Longan-RNA:1:531:+ | 40 | 13.283 | BAF27412.1\| Os11g0112300 |
|  | Unigene48745_Longan-RNA:1:331:+ | 288 | 20.9743 | unnamed protein product |
|  | Unigene47246_Longan-RNA:1:314:+ | 37 | 12.9342 | AT5g24690/MXC17_8 |
|  | Unigene60807_Longan-RNA:1:606:+ | 288 | 10.8367 | BAF29690.2\| Os12g0428600 |
|  | Unigene60497_Longan-RNA:1:591:+ | 396 | 30.0632 | multispanning membrane protein-like |
|  | Unigene56534_Longan-RNA:1:465:+ | 357 | 16.0803 | bromodomain protein |
|  | Unigene57317_Longan-RNA:1:485:+ | 31 | 25.5187 | cell division cycle protein |
|  | Unigene64881_Longan-RNA:1:884:+ | 761 | 13.2837 | 54-kD signal recognition particle (SRP) specific protein |
|  | Unigene61185_Longan-RNA:1:623:+ | 170 | 55.2324 | protein kinase-like protein |
|  |  | 171 | 66.0691 | 1 |
|  | Unigene67645_Longan-RNA:1:1436:+ | 949 | 30.7623 | phosphoglycerate/bisphosphoglycerate mutase family protein |
|  | Unigene32503_Longan-RNA:1:214:+ | 50 | 19.2265 | similar to chaperonin containing TCP-1 complex gamma chain |
|  | Unigene50551_Longan-RNA:1:356:+ | 43 | 10.4872 | aluminum-induced protein |
| miR1513 | Unigene2471_Longan-RNA:1:203:+ | 88 | 25.5187 |  |
| miR1514 | Unigene58992_Longan-RNA:1:535:+ | 182 | 13.2837 | sphere organelles protein-related |
| miR1517 | Unigene68263_Longan-RNA:1:1773:+ | 845 | 18.5273 | BAF17199.1\| Os05g0354300 |
| miR1519 | Unigene10890_Longan-RNA:1:524:+ | 48 | 12.235 | 5-formyltetrahydrofolate cyclo-ligase |
|  | Unigene68756_Longan-RNA:1:2542:+ | 2313 | 26.917 | BAF13785.1\| Os03g0844500 |
|  | Unigene65781_Longan-RNA:1:990:+ | 445 | 41.9486 | sec23/sec24 transport family protein |
|  |  | 446 | 27.2666 |  |
|  | Unigene6577_Longan-RNA:1:460:+ | 343 | 20.6247 | BAH93340.1\| Os06g0159750 |
|  |  | 344 | 24.8196 |  |
|  | Unigene32798_Longan-RNA:1:215:+ | 85 | 11.5359 | Phytochrome-associated serine/threonine protein phosphatase |
|  | Unigene67667_Longan-RNA:1:1441:+ | 68 | 13.6333 | centromeric protein-related |
|  | Unigene27348_Longan-RNA:1:192:+ | 141 | 14.3324 | UPF0183 protein |
|  |  | 142 | 16.7795 |  |
|  | Unigene42658_Longan-RNA:1:273:+ | 33 | 13.6333 | DNAJ heat shock N-terminal domain-containing protein |
|  | Unigene55447_Longan-RNA:1:440:+ | 387 | 18.5273 | cyclin-related protein 1 |
|  | Unigene57876_Longan-RNA:1:500:+ | 99 | 17.129 | ATM (ATAXIA-TELANGIECTASIA MUTATED); 1-phosphatidylinositol-3-phosphate 5-kinase |
|  | Unigene7768_Longan-RNA:1:2179:+ | 1968 | 15.3812 | DEAD-box ATP-dependent RNA helicase |
|  |  | 1969 | 10.4872 |  |
| miR1519,miR2628 | Unigene40913_Longan-RNA:1:261:+ | 220 | 67.8169 | uracil phosphoribosyl transferase like protein |
|  |  | 221 | 19.576 |  |
|  |  | 222 | 25.5187 |  |
| miR1521 | Unigene13345_Longan-RNA:1:2303:+ | 1153 | 11.8854 | ARF-GAP domain 4 |
| miR1522 | Unigene51486_Longan-RNA:1:369:+ | 271 | 20.9743 | BAF14201.1\| Os04g0228000 |
|  |  | 272 | 13.2837 |  |
| miR1525 | Unigene67508_Longan-RNA:1:1386:+ | 718 | 11.1863 | anion exchanger family protein |
|  |  | 719 | 13.9829 |  |
|  | Unigene56630_Longan-RNA:1:467:+ | 328 | 12.235 | alpha-biotin transcarboxylase subunit |
|  |  | 329 | 52.4358 |  |
|  | Unigene55084_Longan-RNA:1:432:+ | 126 | 15.3812 | 6-phosphogluconolactonase |
|  |  | 128 | 12.5846 |  |
|  |  | 129 | 10.1376 | 1 |
| miR1533 | Unigene37128_Longan-RNA:1:239:+ | 90 | 33.9085 | multidrug resistance protein ABC transporter family |
|  | Unigene11427_Longan-RNA:1:471:+ | 259 | 12.235 | BAF19922.1\| Os06g0602600 |
|  | Unigene8307_Longan-RNA:1:624:+ | 42 | 24.8196 | At5g19300 |
|  |  | 43 | 22.7222 |  |
|  | Unigene50378_Longan-RNA:1:353:+ | 275 | 11.5359 | BAF17792.1\| Os05g0486200 |
|  |  | 279 | 43.6965 |  |
| miR1535 | Unigene24196_Longan-RNA:1:180:+ | 58 | 21.6735 | transcription factor |
|  |  | 59 | 14.3324 |  |
| miR1849 | Unigene53344_Longan-RNA:1:399:+ | 201 | 15.7307 | shikimate kinase |
|  | Unigene1792_Longan-RNA:1:998:+ | 492 | 22.7222 | cytochrome c oxidase subunit 2 |
|  |  | 493 | 25.5187 |  |
| miR1856 | Unigene66200_Longan-RNA:1:1058:+ | 262 | 11.5359 | pentatricopeptide repeat-containing protein |
| miR1863 | Unigene60179_Longan-RNA:1:578:+ | 321 | 10.4872 | cellulose synthase |
|  |  | 322 | 17.8282 |  |
| miR1887 | Unigene35588_Longan-RNA:1:230:+ | 159 | 10.8367 | hypothetical protein isoform 2 |
|  | Unigene14764_Longan-RNA:1:1140:+ | 717 | 12.235 | transcription regulator |
|  | Unigene58571_Longan-RNA:1:521:+ | 471 | 104.1724 | equilibrative nucleoside transporter |
|  |  | 476 | 22.3726 |  |
|  | Unigene61714_Longan-RNA:1:647:+ | 614 | 275.4627 | SPHK1 (SPHINGOSINE KINASE 1); D-erythro-sphingosine kinase/ diacylglycerol kinase/ sphinganine kinase |
|  |  | 615 | 79.3528 |  |
|  |  | 616 | 359.3599 |  |
|  |  | 617 | 37.4042 |  |
| miR1917 | Unigene55497_Longan-RNA:1:441:+ | 404 | 20.9743 | ATE2 (ARGININE-TRNA PROTEIN TRANSFERASE 2); arginyltransferase |
|  | Unigene25194_Longan-RNA:1:184:+ | 58 | 47.5418 | luminal binding protein (BiP) |
|  | Unigene52778_Longan-RNA:1:389:+ | 161 | 15.0316 | BAF20504.1\| Os06g0716700 |
|  |  | 164 | 18.8769 |  |
|  | Unigene64385_Longan-RNA:1:833:+ | 688 | 12.584 | flavanone-3-hydroxylase |
|  | Unigene65175_Longan-RNA:1:915:+ | 241 | 12.9342 | GTP-binding family protein |
|  |  | 242 | 12.235 |  |
|  | Unigene58768_Longan-RNA:1:528:+ | 138 | 17.4786 | fimbrin-like protein AtFim2 |
|  |  | 139 | 10.1376 |  |
| miR1919 | Unigene43983_Longan-RNA:1:283:+ | 150 | 22.3726 | nonclathrin coat protein zeta1-COP |
|  | Unigene66583_Longan-RNA:1:1136:+ | 267 | 14.3324 | class III peroxidase |
| miR2088 | Unigene66784_Longan-RNA:1:1177:+ | 327 | 12.7222 | similar to pyrroline-5-carboxylate synthetase |
|  | Unigene60624_Longan-RNA:1:597:+ | 316 | 14.682 | similar to Os01g0104000 |
|  |  | 317 | 10.1376 |  |
|  | Unigene13582_Longan-RNA:1:1723:+ | 628 | 11.5359 | cytochrome P450 |
| miR2090 | Unigene49324_Longan-RNA:1:339:+ | 260 | 14.682 | Expressed protein |
| miR2092 | Unigene51494_Longan-RNA:1:369:+ | 127 | 43.6965 | BAF16795.2\| Os05g0200500 |
| miR2093 | Unigene63651_Longan-RNA:1:770:+ | 661 | 25.8683 | ATEB1B |
|  | Unigene66974_Longan-RNA:1:1228:+ | 964 | 11.5359 | ATTIP49A/RIN1 |
|  | Unigene67993_Longan-RNA:1:1583:+ | 1028 | 131.0895 | RNA recognition motif-containing protein |
|  | Unigene62392_Longan-RNA:1:684:+ | 414 | 15.7307 | Pre-mRNA-splicing factor ini1 |
|  | Unigene24115_Longan-RNA:1:180:+ | 150 | 25.5187 | 2,3-bisphosphoglycerate-independent phosphoglycerate mutase |
|  | Unigene65816_Longan-RNA:1:995:+ | 875 | 31.811 | At1g30300 [Arabidopsis thaliana] |
|  |  | 876 | 25.1692 |  |
| miR2099 | Unigene12344_Longan-RNA:1:1529:+ | 1193 | 24.1205 | At1g72880 [Arabidopsis thaliana] |
|  |  | 1194 | 11.1863 |  |
|  | Unigene63288_Longan-RNA:1:742:+ | 309 | 20.6247 | CTV.2 |
|  | Unigene67373_Longan-RNA:1:1338:+ | 1315 | 11.1863 | AT4g37080/C7A10_280 |
|  | Unigene13614_Longan-RNA:1:1813:+ | 1503 | 11.1863 | FAD-binding domain-containing protein |
|  | Unigene57502_Longan-RNA:1:490:+ | 20 | 16.4299 | diphosphomevelonate decarboxylase |
|  | Unigene48538_Longan-RNA:1:329:+ | 67 | 153.4621 | BAM1 (BETA-AMYLASE 1); beta-amylase |
|  |  | 68 | 141.227 |  |
|  |  | 69 | 115.7083 |  |
|  | Unigene66698_Longan-RNA:1:1161:+ | 180 | 12.235 | BAF04643.1\| Os01g0276500 |
|  |  | 184 | 11.5359 |  |
|  |  | 188 | 55.2324 | 1 |
|  |  | 189 | 141.227 |  |
|  |  | 188 | 55.2324 | 1 |
|  |  | 189 | 141.227 |  |
|  | Unigene48900_Longan-RNA:1:333:+ | 135 | 12.5846 | polycomb group protein EMF2 |
|  |  | 136 | 54.5332 |  |
|  | Unigene66126_Longan-RNA:1:1046:+ | 177 | 22.7222 | speckle-type POZ protein |
|  |  | 178 | 26.2179 | 1 |
|  |  | 179 | 37.4042 |  |
|  | Unigene63514_Longan-RNA:1:760:+ | 572 | 41.5991 | MYB transcription factor |
|  | Unigene50004_Longan-RNA:1:348:+ | 249 | 12.5846 | Similar to 2-oxo acid dehydrogenase, lipoyl-binding site |
| miR2099,miR1426 | Unigene58458_Longan-RNA:1:518:+ | 401 | 26.917 | PP2Ac-2 - Phosphatase 2A isoform 2 belonging to family 2 |
| miR2108 | Unigene68901_Longan-RNA:1:3729:+ | 3109 | 30.7623 | binding |
|  | Unigene37_Longan-RNA:1:1199:+ | 809 | 10.1376 | expressed protein |
|  | Unigene46148_Longan-RNA:1:303:+ | 187 | 21.3239 | expressed protein |
| miR2111 | Unigene51156_Longan-RNA:1:364:+ | 239 | 21.3239 | non-photosynthetic ferredoxin |
| miR2607 | Unigene65766_Longan-RNA:1:989:+ | 416 | 20.2752 | anthranilate phosphoribosyltransferase-like protein |
|  | Unigene48452_Longan-RNA:1:328:+ | 160 | 19.2265 | ATP-dependent Clp protease |
| miR2607,miR854 | Unigene7910_Longan-RNA:1:1013:+ | 106 | 16.0803 | BAF09188.1\| Os02g0588300 |
|  |  | 107 | 199.6056 |  |
|  |  | 108 | 350.6206 |  |
|  |  | 109 | 199.6056 |  |
|  |  | 110 | 350.6206 |  |
|  |  | 111 | 198.2073 |  |
|  |  | 112 | 212.5397 |  |
|  |  | 113 | 43.3469 |  |
| miR2612 | Unigene60951_Longan-RNA:1:612:+ | 81 | 78.3041 | matrix metalloprotease 1 |
|  | Unigene14152_Longan-RNA:1:870:+ | 179 | 14.3324 | pentatricopeptide repeat protein |
|  | Unigene66667_Longan-RNA:1:1153:+ | 1090 | 41.2495 | chromatin remodeling complex subunit |
|  |  | 1091 | 30.7623 |  |
|  | Unigene14075_Longan-RNA:1:1327:+ | 727 | 12.235 | Galactoside 2-alpha-L-fucosyltransferase |
|  | Unigene59009_Longan-RNA:1:536:+ | 253 | 13.6333 | leucine-rich repeat transmembrane protein kinase |
| miR2628 | Unigene2357_Longan-RNA:1:1932:+ | 800 | 11.8854 | At5g23850 |
|  | Unigene68133_Longan-RNA:1:1671:+ | 892 | 17.129 | BAF17086.1\| Os05g0316000 |
|  | Unigene9579_Longan-RNA:1:1254:+ | 944 | 10.1376 | WD-40 repeat family protein |
|  |  | 945 | 11.8854 |  |
|  | Unigene63099_Longan-RNA:1:728:+ | 22 | 31.811 | b-zip transcription factor |
|  |  | 23 | 11.1863 |  |
|  | Unigene67737_Longan-RNA:1:1469:+ | 854 | 22.3726 | myb family transcription factor |
|  | Unigene5310_Longan-RNA:1:292:+ | 116 | 12.235 | hydrolase family protein 17 |
|  | Unigene15195_Longan-RNA:1:1365 | 274 | 12.235 | knotted1-like homeobox transcription factor |
|  | Unigene12577_Longan-RNA:1:651:+ | 180 | 11.1863 | PRLI-interacting factor-related |
|  | Unigene37730_Longan-RNA:1:242:+ | 95 | 15.0316 | reticulon family protein |
| miR2634 | Unigene13090_Longan-RNA:1:362:+ | 126 | 31.811 | vesicle transport v-SNARE 13 |
|  | Unigene60544_Longan-RNA:1:593:+ | 561 | 40.8999 | cell division cycle protein |
|  |  | 562 | 20.8999 |  |
|  | Unigene65666_Longan-RNA:1:976:+ | 612 | 13.6333 | BAF03901.1\| Os01g0141900 |
|  | Unigene58052_Longan-RNA:1:505:+ | 299 | 11.186 | Contains S1 RNA binding domain |
| miR2641 | Unigene14520_Longan-RNA:1:1899 | 1532 | 10.4872 | mannan synthase |
|  | Unigene7748_Longan-RNA:1:552:+ | 282 | 12.235 | aspartyl protease family protein |
|  | Unigene16351_Longan-RNA:1:151:+ | 64 | 13.2837 | Thioredoxin-like protein Clot |
|  |  | 65 | 22.023 |  |
| miR2642 | Unigene34715_Longan-RNA:1:225:+ | 37 | 103.4733 | similar to oxysterol-binding family protein |
|  |  | 38 | 78.6537 |  |
|  |  | 39 | 13.6333 |  |
|  | Unigene37306_Longan-RNA:1:240:+ | 162 | 12.235 | RUB1 conjugating enzyme |
|  | Unigene25795_Longan-RNA:1:186:+ | 130 | 14.682 | At5g15120 |
|  | Unigene34662_Longan-RNA:1:225:+ | 150 | 166.0467 | alpha-biotin transcarboxylase subunit |
|  |  | 151 | 73.41 |  |
| miR2643 | Unigene64902_Longan-RNA:1:886:+ | 563 | 75.857 | At4g30630 |
|  | Unigene55953_Longan-RNA:1:452:+ | 421 | 16.0803 | eukaryotic rpb5 RNA polymerase subunit family protein |
|  | Unigene59831_Longan-RNA:1:565:+ | 343 | 12.235 | villin 2 |
| miR2644 | Unigene15225_Longan-RNA:1:5187:+ | 5103 | 10.8367 | BAF04136.2\| Os01g0182900 |
| miR2654 | Unigene64127_Longan-RNA:1:810:+ | 680 | 13.6333 | AKINbetagammaI |
|  | Unigene13969_Longan-RNA:1:480:+ | 69 | 11.8854 | Strong similarity to phosphoribosylanthranilate transferase |
| miR2658 | Unigene56934_Longan-RNA:1:475:+ | 251 | 16.7795 | clathrin assembly protein AP17-like protein |
|  | Unigene41588_Longan-RNA:1:266:+ | 138 | 11.5359 | brassinosteroid signalling positive regulator-related （BES1/BZR1 homolog protein 3） |
|  | Unigene52515_Longan-RNA:1:385:+ | 89 | 13.2837 | ribosomal protein L12 |
| miR2661 | Unigene68036_Longan-RNA:1:1611:+ | 52 | 11.1863 | Cysteine protease ATG4 |
| miR2663 | Unigene9170_Longan-RNA:1:943:+ | 758 | 14.3324 | WRKY transcription factor 19 |
| miR2667 | Unigene62640_Longan-RNA:1:699:+ | 668 | 11.1863 | BAF06528.1\| Os01g0815700 |
|  |  | 669 | 10.8367 |  |
|  | Unigene60220_Longan-RNA:1:580:+ | 181 | 81.1007 | receptor-like protein kinase precusor-like protein |
|  | Unigene61368_Longan-RNA:1:631:+ | 547 | 18.5273 | adenylosuccinate-AMP lyase |
|  | Unigene20648_Longan-RNA:1:166:+ | 108 | 41.9486 | RAN GTPase activating protein 2 |
| miR2670 | Unigene26856_Longan-RNA:1:191:+ | 172 | 16.7795 | At2g36720 |
| miR2671 | Unigene67655_Longan-RNA:1:1438:+ | 1201 | 16.7795 | conserved hypothetical protein |
|  |  | 1202 | 62.5734 |  |
| miR2673 | Unigene11018_Longan-RNA:1:400:+ | 157 | 50.6879 | predicted protein |
|  |  | 158 | 68.1665 |  |
|  |  | 159 | 79.3528 |  |
|  |  | 160 | 50.6879 |  |
|  |  | 161 | 68.1665 |  |
|  |  | 162 | 26.917 |  |
|  | Unigene59392_Longan-RNA:1:548:+ | 520 | 201.0038 | AP2/ERF domain-containing transcription factor |
|  |  | 523 | 201.0038 |  |
|  |  | 524 | 27.6162 |  |
|  |  | 525 | 11.5359 |  |
|  | Unigene12331_Longan-RNA:1:977:+ | 372 | 10.8367 | expressed protein |
|  | Unigene51623_Longan-RNA:1:371:+ | 345 | 201.0038 | zinc finger family protein |
|  |  | 346 | 27.6162 |  |
|  |  | 347 | 20.9743 |  |
|  |  | 348 | 201.0038 |  |
|  |  | 349 | 27.6162 |  |
|  |  | 350 | 20.9743 |  |
|  |  | 351 | 26.2179 |  |
|  | Unigene12251_Longan-RNA:1:831:+ | 808 | 24.1205 | preproMP73 |
|  |  | 809 | 31.1119 |  |
|  |  | 810 | 23.4213 |  |
|  |  | 811 | 30.0632 |  |
|  | Unigene51982_Longan-RNA:1:376:+ | 310 | 32.5102 | UDP-glucose glucosyltransferase |
|  | Unigene12541_Longan-RNA:1:861:+ | 206 | 17.4786 | acuolar protein sorting-associated protein 41 homolog |
|  |  | 214 | 30.4128 |  |
|  |  | 215 | 16.0803 |  |
|  | Unigene49256_Longan-RNA:1:338:+ | 314 | 10.8367 | expressed protein |
|  |  | 315 | 15.7307 |  |
|  | Unigene7458_Longan-RNA:1:885:+ | 728 | 25.5187 | At4g04630 |
|  | Unigene63085_Longan-RNA:1:727:+ | 431 | 22.7222 | zinc finger (C3HC4-type RING finger) family protein |
|  |  | 432 | 111.163 |  |
|  | Unigene60784_Longan-RNA:1:605:+ | 118 | 17.8282 | At1g73320 |
|  | Unigene25062_Longan-RNA:1:183:+ | 130 | 41.2495 | Contains homology to DNAJ heatshock protein |
|  |  | 131 | 14.3324 |  |
|  | Unigene40503_Longan-RNA:1:258:+ | 123 | 17.8282 | PUB38 (PLANT U-BOX 38); ubiquitin-protein ligase |
|  | Unigene11625_Longan-RNA:1:765:+ | 557 | 19.9256 | regulator of chromosome condensation family protein |
|  | Unigene9976_Longan-RNA:1:1941:+ | 700 | 15.7307 | dehydration-responsive protein-related |
|  | Unigene7801_Longan-RNA:1:2383:+ | 119 | 27.6162 | ETO1 (ETHYLENE OVERPRODUCER 1); protein binding, bridging |
|  | Unigene65212_Longan-RNA:1:920:+ | 670 | 53.1349 | ATP binding protein |
|  |  | 671 | 17.8282 |  |
|  |  | 672 | 20.9743 |  |
|  |  | 673 | 201.0038 |  |
|  |  | 674 | 27.6162 |  |
|  |  | 675 | 12.9342 |  |
|  | Unigene5219_Longan-RNA:1:1157:+ | 1025 | 13.9829 | aquaporin, MIP family, PIP subfamily |
|  |  | 1028 | 19.9256 |  |
|  |  | 1029 | 68.1665 |  |
|  |  | 1030 | 79.3528 |  |
|  |  | 1031 | 50.6879 |  |
|  |  | 1032 | 68.1665 |  |
|  |  | 1033 | 79.3528 |  |
|  |  | 1034 | 50.6879 |  |
|  |  | 1035 | 66.4187 |  |
|  | Unigene68720_Longan-RNA:1:2435:+ | 2229 | 201.0038 | subtilase family protein |
|  |  | 2230 | 27.6162 |  |
|  | Unigene65260_Longan-RNA:1:925:+ | 900 | 25.5187 | f-box family protein |
|  |  | 901 | 27.6162 |  |
|  |  | 902 | 20.9743 |  |
|  |  | 903 | 201.0038 |  |
|  |  | 904 | 27.6162 |  |
|  |  | 905 | 11.5359 |  |
|  | Unigene49108_Longan-RNA:1:336:+ | 305 | 201.0038 | At5g56880 |
|  |  | 306 | 27.6162 |  |
|  |  | 307 | 12.9342 |  |
|  |  | 313 | 18.1777 |  |
|  | Unigene65724_Longan-RNA:1:983:+ | 320 | 19.576 | inducer of CBF expression 1 |
|  |  | 533 | 13.9829 |  |
|  | Unigene8769_Longan-RNA:1:2106:+ | 507 | 17.4786 | Os06g0109400 [Oryza sativa Japonica Group] >gi\|113594616\|dbj\|BAF18490.1\| Os06g0109400 [Oryza sativa Japonica Group] |
|  |  | 519 | 17.4786 |  |
|  | Unigene61401_Longan-RNA:1:632:+ | 605 | 20.9743 | pathogenesis-related protein 1-like protein |
|  |  | 606 | 201.0038 |  |
|  |  | 607 | 27.6162 |  |
|  |  | 608 | 20.9743 |  |
|  |  | 609 | 201.0038 |  |
|  |  | 610 | 27.6162 |  |
|  |  | 611 | 11.5359 |  |
|  |  | 612 | 15.7307 |  |
|  | Unigene64539_Longan-RNA:1:849:+ | 781 | 201.0038 | DC1 domain-containing protein |
|  |  | 782 | 27.6162 |  |
|  |  | 783 | 11.5359 |  |
|  | Unigene67268_Longan-RNA:1:1305:+ | 75 | 20.9743 | Protein Brevis radix-like 2; |
|  |  | 76 | 201.0038 |  |
|  |  | 77 | 27.6162 |  |
|  |  | 78 | 20.9743 |  |
|  |  | 79 | 26.2179 |  |
|  | Unigene3834_Longan-RNA:1:2424:+ | 1482 | 201.0038 | transducin family protein / WD-40 repeat family protein |
|  |  | 1483 | 27.6162 | 1 |
|  |  | 1484 | 11.5359 | 1 |
|  |  | 1485 | 15.7307 |  |
|  | Unigene14737_Longan-RNA:1:2219:+ | 671 | 201.0038 | EST gb\|ATTS1136 comes from this gene |
|  |  | 672 | 27.6162 |  |
|  |  | 673 | 12.9342 |  |
|  | Unigene3285_Longan-RNA:1:1385:+ | 259 | 17.4786 | At5g38690/MBB18_24 |
|  | Unigene68424_Longan-RNA:1:1917:+ | 329 | 17.4786 | bzip-like transcription factor-like protein |
|  | Unigene54104_Longan-RNA:1:413:+ | 345 | 17.478 | GIL1 (GRAVITROPIC IN THE LIGHT) |
|  | Unigene33810_Longan-RNA:1:220:+ | 167 | 55.5819 | transcription factor |
|  |  | 168 | 12.9342 |  |
|  | Unigene14118_Longan-RNA:1:841:+ | 812 | 15.0316 | G1-like7 protein |
|  |  | 813 | 10.1376 |  |
|  | Unigene48976_Longan-RNA:1:334:+ | 74 | 12.235 | conserved hypothetical protein |
|  | Unigene36709_Longan-RNA:1:236:+ | 121 | 15.3812 | high mobility group family |
|  |  | 122 | 76.2067 |  |
|  | Unigene67649_Longan-RNA:1:1437:+ | 1411 | 27.6162 | At4g28230 |
|  |  | 1412 | 20.9743 |  |
|  |  | 1413 | 201.0038 |  |
|  |  | 1414 | 27.6162 |  |
|  |  | 1415 | 20.9743 |  |
|  |  | 1416 | 201.0038 |  |
|  |  | 1417 | 27.6162 |  |
|  | Unigene61964_Longan-RNA:1:661:+ | 637 | 201.0038 | predicted protein |
|  |  | 638 | 27.6162 |  |
|  |  | 639 | 20.9743 |  |
|  |  | 640 | 201.0038 |  |
|  |  | 641 | 27.6162 |  |
| miR2676 | Unigene15129_Longan-RNA:1:3004:+ | 2558 | 13.6333 | Cl-channel clc-7 |
|  |  | 2559 | 11.8854 |  |
|  |  | 2560 | 26.5675 |  |
|  | Unigene64956_Longan-RNA:1:891:+ | 433 | 52.7854 | DNA binding zinc finger protein |
|  |  | 434 | 39.5016 |  |
|  |  | 439 | 13.9829 |  |
| miR2863 | Unigene59802_Longan-RNA:1:564:+ | 371 | 29.364 | mitochondrial substrate carrier family protein |
|  |  | 372 | 13.2837 |  |
|  |  | 373 | 35.6563 |  |
|  |  | 374 | 41.5991 |  |
| miR2870 | Unigene12695_Longan-RNA:1:1536:+ | 1245 | 37.0546 | octicosapeptide/Phox/Bem1p(PB1) domain-containing protein |
|  | Unigene2501_Longan-RNA:1:226:+ | 199 | 22.7222 | dehydration-responsive element binding protein 3 |
|  |  | 202 | 10.1376 |  |
|  | Unigene55712_Longan-RNA:1:446:+ | 168 | 38.802 | hydrolase |
| miR2911,miR2612 | Unigene63998_Longan-RNA:1:799:+ | 575 | 26.2179 | calcium dependent protein kinase 13 |
| miR2931 | Unigene65225_Longan-RNA:1:922:+ | 67 | 10.8367 | Outward rectifying potassium channel |
|  | Unigene66221_Longan-RNA:1:1061:+ | 444 | 13.2837 | zinc-binding family protein |
|  |  | 445 | 30.7623 |  |
| miR2933 | Unigene55441_Longan-RNA:1:440:+ | 144 | 41.9486 | predicted protein |
|  | Unigene11631_Longan-RNA:1:972:+ | 208 | 32.1606 | AT4g37020 |
|  |  | 211 | 12.5846 |  |
|  | Unigene2452_Longan-RNA:1:1907:+ | 1557 | 11.5359 | receptor-like protein kinase-like protein |
|  |  | 1558 | 11.5359 |  |
| miR2933,miR854 | Unigene60858_Longan-RNA:1:608:+ | 587 | 19.576 | unnamed protein product |
|  |  | 588 | 11.5359 |  |
|  |  | 589 | 41.5991 |  |
| miR2936,miR854 | Unigene64251_Longan-RNA:1:820:+ | 239 | 16.4299 | WRKY transcription factor 13 |
| miR2936,miR854 | Unigene62874_Longan-RNA:1:713:+ | 19 | 15.0316 | RNA helicase-like protein |
|  |  | 20 | 10.8367 |  |
| miR2948 | Unigene47163_Longan-RNA:1:313:+ | 128 | 24.1205 | similar to elongation factor 2 |
|  |  | 129 | 31.4615 |  |
| miR3433 | Unigene36847_Longan-RNA:1:237:+ | 147 | 11.5359 | predicted protein |
| miR3435 | Unigene418_Longan-RNA:1:375:+ | 353 | 10.4872 | unnamed protein product |
|  | Unigene66602_Longan-RNA:1:1139:+ | 755 | 11.5359 | PAN domain-containing protein |
| miR3437 | Unigene61157_Longan-RNA:1:622:+ | 424 | 26.2179 | AT5g49210/K21P3_8 |
|  | Unigene47315_Longan-RNA:1:315:+ | 236 | 57.6794 | BAF15782.1\| Os04g0615200 |
| miR3437,miR1533 | Unigene16181_Longan-RNA:1:151:+ | 127 | 44.7452 | cyclopropyl isomerase |
|  |  | 128 | 93.6853 |  |
|  |  | 130 | 16.7795 |  |
|  |  | 131 | 11.8854 |  |
| miR3440 | Unigene56572_Longan-RNA:1:466:+ | 17 | 10.1376 | agenet domain-containing protein |
|  | Unigene10808_Longan-RNA:1:1156:+ | 849 | 11.8854 | lectin-receptor like protein kinase 3 |
|  | Unigene55868_Longan-RNA:1:450:+ | 317 | 22.023 | BAF22878.1\| Os08g0139000 |
|  | Unigene67048_Longan-RNA:1:1244:+ | 745 | 10.8367 | CLASP (CLIP-ASSOCIATED PROTEIN); binding |
| miR3442 | Unigene8967_Longan-RNA:1:444:+ | 382 | 11.1863 | zinc ion binding |
|  |  | 383 | 15.7307 |  |
|  | Unigene62862_Longan-RNA:1:713:+ | 592 | 12.235 | HhH-GPD base excision DNA repair protein-related |
|  | Unigene68345_Longan-RNA:1:1836:+ | 713 | 40.8999 | Zinc finger, CCCH-type; Sugar transporter superfamily |
|  |  | 714 | 24.1205 |  |
|  | Unigene61787_Longan-RNA:1:650:+ | 547 | 18.1777 | BAF06279.1\| Os01g0768200 |
|  | Unigene9855_Longan-RNA:1:498:+ | 326 | 12.235 | plastid DNA-binding protein |
|  | Unigene68353_Longan-RNA:1:1842:+ | 1566 | 70.264 | bHLH transcription factor bHLH003 |
|  | Unigene16872_Longan-RNA:1:153:+ | 34 | 12.9342 | methionine synthase |
|  | Unigene51559_Longan-RNA:1:370:+ | 273 | 33.5589 | matrixin family protein |
|  |  | 274 | 26.2179 |  |
|  | Unigene61058_Longan-RNA:1:618:+ | 368 | 75.5075 | BAF23886.1\| Os08g0459100 |
|  | Unigene40299_Longan-RNA:1:257:+ | 40 | 19.9256 | transformer-SR ribonucleo protein |
|  | Unigene59714_Longan-RNA:1:560:+ | 153 | 36.3555 | bZip transcription factor |
|  | Unigene68619_Longan-RNA:1:2188:+ | 1675 | 13.9829 | At2g15560 |
|  | Unigene44039_Longan-RNA:1:284:+ | 138 | 11.5359 | multicatalytic endopeptidase complex, proteasome precursor, beta subunit |
| miR3444 | Unigene46250_Longan-RNA:1:304:+ | 227 | 13.283 | aldehyde dehydrogenase family 7 member A1 |
|  |  | 231 | 13.2837 |  |
| miR3444,miR827 | Unigene61988_Longan-RNA:1:662:+ | 378 | 49.6392 | CIPK4 (CBL-interacting protein kinase 4); ATP binding / kinase/ protein kinase/ protein serine/threonine kinase |
|  |  | 381 | 12.9342 |  |
| miR3452 | Unigene65885_Longan-RNA:1:1008:+ | 675 | 36.0059 | hAT-like transposase |
|  |  | 676 | 26.5675 |  |
|  | Unigene57851_Longan-RNA:1:499:+ | 215 | 11.1863 | OTU-like cysteine protease family protein |
|  | Unigene64726_Longan-RNA:1:867:+ | 244 | 36.0059 | flavonol synthase-like protein |
| miR3452,miR854 | Unigene55474_Longan-RNA:1:440:+ | 419 | 350.6206 | magnesium transporter |
|  |  | 420 | 183.1757 |  |
|  |  | 421 | 181.7774 |  |
|  |  | 422 | 32.1606 |  |
| miR3454 | Unigene59682_Longan-RNA:1:558:+ | 359 | 50.3384 | ras-related GTP-binding protein |
| miR3458 | Unigene61144_Longan-RNA:1:622:+ | 443 | 62.9229 | nectarin IV |
|  | Unigene41856_Longan-RNA:1:267:+ | 107 | 25.5187 | ChaC-like family protein |
|  |  | 109 | 14.682 |  |
| miR3515 | Unigene518_Longan-RNA:1:801:+ | 454 | 12.9342 | leucine-rich repeat family protein /protein kinase family protein |
|  |  | 455 | 13.6333 |  |
|  | Unigene22134_Longan-RNA:1:172:+ | 86 | 11.8854 | 2OG-Fe(II) oxygenase |
|  |  | 97 | 22.3726 |  |
|  | Unigene49237_Longan-RNA:1:338:+ | 190 | 91.5878 | Glycosyl hydrolases family 38 protein, expressed |
|  |  | 191 | 15.3812 |  |
|  | Unigene56933_Longan-RNA:1:474:+ | 370 | 18.1777 | similar to catalytic |
|  | Unigene62679_Longan-RNA:1:701:+ | 447 | 36.0059 | methionine sulfoxide reductase type |
|  |  | 448 | 674.6738 |  |
|  |  | 449 | 112.9117 |  |
| miR3624 | Unigene68700_Longan-RNA:1:2403:+ | 2364 | 10.8367 | pha-glucosidase 1 (AGLU1) |
| miR3630 | Unigene67639_Longan-RNA:1:1434:+ | 1222 | 14.3324 | phosphatidylinositol 3-and 4-kinase family protein |
|  |  | 1223 | 15.0316 |  |
| miR3949,miR2667 | Unigene32776_Longan-RNA:1:215:+ | 41 | 27.266 | BAF22969.1\| Os08g0162100 |
| miR3981 | Unigene208_Longan-RNA:1:307:+ | 156 | 10.4872 | ARK3 (A. THALIANA RECEPTOR KINASE 3); kinase/ transmembrane receptor protein serine/threonine kinase |
|  | Unigene67304_Longan-RNA:1:1315:+ | 730 | 13.2837 | membrane protein-like |
|  |  | 737 | 62.2238 |  |
|  | Unigene14866_Longan-RNA:1:1642:+ | 34 | 10.4872 | YebC-related |
|  |  | 35 | 13.9829 |  |
|  | Unigene62185_Longan-RNA:1:673:+ | 280 | 10.8367 | pseudo response regulator |
|  |  | 281 | 12.9342 |  |
|  | Unigene13499_Longan-RNA:1:410:+ | 366 | 28.3153 | N2,N2-dimethylguanosine tRNA methyltransferase family protein |
|  |  | 367 | 18.5273 |  |
| miR4244 | Unigene67148_Longan-RNA:1:1269:+ | 1042 | 66.7682 | expressed protein |
|  |  | 1043 | 56.6306 |  |
|  | Unigene64528_Longan-RNA:1:847:+ | 113 | 20.6247 | EST gb\|Z37689 comes from this gene |
| miR4372 | Unigene13439_Longan-RNA:1:948:+ | 663 | 29.7136 | mitochondrial dicarboxylate carrier |
| miR4388 | Unigene68331_Longan-RNA:1:1829:+ | 1435 | 11.186 | DGK1 (DIACYLGLYCEROL KINASE1); calcium ion binding / diacylglycerol kinase |
| miR4413 | Unigene67224_Longan-RNA:1:1290:+ | 1215 | 12.5846 | binding / small GTPase regulator |
|  | Unigene60683_Longan-RNA:1:600:+ | 548 | 11.5359 | expressed protein |
|  | Unigene65637_Longan-RNA:1:971:+ | 667 | 16.0803 | F-box family protein |
|  | Unigene12748_Longan-RNA:1:825:+ | 644 | 13.9829 | F-box family protein |
|  | Unigene68454_Longan-RNA:1:1944:+ | 1921 | 63.6221 | 3,4-dihydroxy-2-butanone-4-phosphate synthase/ GTP cyclohydrolase II |
|  |  | 1922 | 33.9085 |  |
| miR5014 | Unigene34800_Longan-RNA:1:226:+ | 17 | 13.283 | phosphatidylinositol synthase |
|  | Unigene42431_Longan-RNA:1:271:+ | 98 | 29.364 | transcription factor, putative |
|  |  | 99 | 27.2666 |  |
|  | Unigene64914_Longan-RNA:1:887:+ | 865 | 12.235 | cytoplasmic aconitate hydratase |
|  | Unigene66267_Longan-RNA:1:1070:+ | 514 | 12.235 | CCHC-type integrase |
| miR5020 | Unigene10392_Longan-RNA:1:1938:+ | 1878 | 13.6333 | pentatricopeptide repeat-containing protein |
|  | Unigene36869_Longan-RNA:1:237:+ | 46 | 10.487 | BAF22366.1\| Os07g0645100 |
|  |  | 47 | 11.1863 |  |
|  |  | 48 | 11.8854 |  |
| miR5021,miR854 | Unigene2646_Longan-RNA:1:1883:+ | 79 | 11.8854 | DnaJ homologue |
|  |  | 80 | 14.682 |  |
|  |  | 82 | 11.8854 |  |
|  |  | 83 | 14.682 |  |
|  |  | 85 | 11.8854 |  |
|  |  | 86 | 14.682 |  |
|  |  | 88 | 11.5359 |  |
| miR5021,miR854 | Unigene11640_Longan-RNA:1:1034:+ | 176 | 11.8854 | At4g04630 |
|  |  | 177 | 14.682 |  |
|  |  | 179 | 11.8854 |  |
|  |  | 180 | 14.682 |  |
|  |  | 182 | 11.5359 |  |
| miR5021,miR854 | Unigene10043_Longan-RNA:1:999:+ | 134 | 11.8854 | GUN4; enzyme binding / tetrapyrrole binding |
|  |  | 135 | 14.682 |  |
|  |  | 137 | 11.8854 |  |
|  |  | 138 | 10.4872 |  |
| miR5021,miR854 | Unigene63406_Longan-RNA:1:751:+ | 263 | 11.8854 | Protein phosphatase AP2C1 |
|  |  | 264 | 14.682 |  |
|  |  | 266 | 11.5359 |  |
| miR5021,miR854 | Unigene64264_Longan-RNA:1:821:+ | 108 | 10.1376 | SERAT3;2 (Serine acetyltransferase 3;2); acetyltransferase/ serine O-acetyltransferase |
|  |  | 109 | 11.8854 |  |
| miR5021,miR854 | Unigene10901_Longan-RNA:1:693:+ | 222 | 78.6537 | NBS-LRR type disease resistance protein |
| miR5028 | Unigene13134_Longan-RNA:1:376:+ | 217 | 16.0803 | expressed protein |
| miR5057 | Unigene48422_Longan-RNA:1:328:+ | 220 | 17.129 | peroxidase 1 |
|  | Unigene54126_Longan-RNA:1:413:+ | 108 | 25.5187 | N-acetylglucosaminyltransferase I |
|  |  | 111 | 14.3324 |  |
|  | Unigene43011_Longan-RNA:1:276:+ | 43 | 15.0316 | f-box family protein |
|  |  | 44 | 10.8367 |  |
|  | Unigene51834_Longan-RNA:1:374:+ | 220 | 12.9342 | AT-hook protein 1 |
| miR5057,miR1137 | Unigene65400_Longan-RNA:1:941:+ | 591 | 10.1376 | peroxidase |
|  |  | 664 | 12.235 |  |
| miR5066 | Unigene2796_Longan-RNA:1:878:+ | 551 | 19.2265 | hypothetical protein |
|  | Unigene49816_Longan-RNA:1:345:+ | 122 | 12.5846 | eukaryotic translation initiation factor 4E |
|  | Unigene9481_Longan-RNA:1:489:+ | 222 | 27.2666 | small molecular heat shock protein 10 |
| miR5071 | Unigene56755_Longan-RNA:1:471:+ | 345 | 54.5332 | integral membrane transporter family protein |
|  | Unigene39416_Longan-RNA:1:252:+ | 172 | 18.177 | aspartyl protease family protein |
| miR5077 | Unigene33919_Longan-RNA:1:221:+ | 138 | 25.5187 | tetratricopeptide repeat-containing protein |
|  |  | 139 | 24.47 |  |
| miR5079 | Unigene55004_Longan-RNA:1:430:+ | 87 | 10.1376 | kinase family protein |
|  | Unigene51027_Longan-RNA:1:363:+ | 191 | 13.2837 | pseudo response regulator |
|  |  | 194 | 20.2752 |  |
|  | Unigene60246_Longan-RNA:1:581:+ | 558 | 38.1033 | bZIP protein-like |
|  |  | 559 | 16.4299 |  |
|  | Unigene31501_Longan-RNA:1:209:+ | 188 | 34.9572 | splicing factor Prp18 family protein |
|  | Unigene43690_Longan-RNA:1:281:+ | 219 | 12.235 | DNA-binding protein |
|  |  | 220 | 36.705 |  |
|  | Unigene62010_Longan-RNA:1:663:+ | 620 | 26.5675 | VP1/ABI3 family regulatory protein-like |
|  | Unigene68655_Longan-RNA:1:2260:+ | 1513 | 64.3212 | At1g31870 |
|  |  | 1514 | 37.4042 |  |
|  | Unigene35139_Longan-RNA:1:228:+ | 147 | 83.5477 | vacuolar sorting receptor homolog |
| miR5085 | Unigene26410_Longan-RNA:1:189:+ | 39 | 28.3153 | chloroplast light-regulated protein |
|  | Unigene51604_Longan-RNA:1:370:+ | 306 | 28.6649 | organic anion transporter |
|  |  | 307 | 12.9342 |  |
|  | Unigene56642_Longan-RNA:1:468:+ | 297 | 32.8598 | dicyanin |
|  |  | 298 | 15.3812 |  |
|  | Unigene60186_Longan-RNA:1:579:+ | 244 | 161.8518 | blue copper protein precursor |
|  | Unigene26410_Longan-RNA:1:189:+ | 39 | 28.3153 | chloroplast light-regulated protein |
| miR5137 | Unigene51221_Longan-RNA:1:365:+ | 178 | 22.7222 | Putative membrane protein ycf1 |
|  |  | 181 | 11.885 |  |
| miR5140 | Unigene49541_Longan-RNA:1:342:+ | 197 | 14.3324 | octicosapeptide/Phox/Bem1p domain-containing protein |
| miR5142 | Unigene14760_Longan-RNA:1:2741:+ | 1201 | 10.8367 | potassium ion transmembrane transporter |
| miR5163 | Unigene32248_Longan-RNA:1:213:+ | 92 | 139.8288 | EFH62165.1\| ALDH6B2 |
|  |  | 93 | 88.0921 |  |
| miR5168 | Unigene60817_Longan-RNA:1:606:+ | 201 | 11.1863 | SEH1H; nucleotide binding |
|  |  | 202 | 14.682 |  |
| miR5170 | Unigene26764_Longan-RNA:1:190:+ | 39 | 68.1665 | LEM3 (ligand-effect modulator 3) family protein / CDC50 family protein |
| miR5170,miR821 | Unigene52657_Longan-RNA:1:387:+ | 348 | 12.235 | sulfite oxidase |
|  |  | 363 | 19.9256 |  |
| miR5171 | Unigene54208_Longan-RNA:1:415:+ | 275 | 44.7452 | KELP; DNA binding / transcription coactivator/ transcription regulator |
| miR5185 | Unigene56575_Longan-RNA:1:466:+ | 34 | 20.6247 | Elongation factor G 1, mitochondrial |
|  | Unigene67019_Longan-RNA:1:1239:+ | 1030 | 17.4786 | NAC domain protein |
|  |  | 1031 | 13.9829 |  |
|  | Unigene4499_Longan-RNA:1:490:+ | 373 | 15.7307 | beta-ketoacyl-coa synthase family protein |
|  | Unigene64508_Longan-RNA:1:845:+ | 392 | 34.258 | catalytic/ protein serine/threonine phosphatase |
| miR5202 | Unigene29454_Longan-RNA:1:201:+ | 105 | 14.3324 | SAR DNA-binding protein-2 |
