# Supplementary material for: Comparative Analysis Reveals Dynamic Changes in miRNAs and Their Targets and Expression during Somatic Embryogenesis in Longan (Dimocarpus longan Lour.)
Source: PLoS One. 2013 Apr 11;8(4):e60337. doi: 10.1371/journal.pone.0060337 (PMC3623967; doi:10.1371/journal.pone.0060337)
Supplement: Supporting Information S1 File containing the following supporting information tables — Table S1. Coserved and non-coserved longan miRNAs. Table S2. Longan-known miRNA targets by predicition method. Table S3. Identifiction targets for longan miRNAs by Degradome sequencing. Table S4. The consensus targets for longan miRNAs obtaining from prediction and degradome analysis. Table S5. Primer sequences. Table S6. The miRNA names, primer sequences. (ZIP) [file pone.0060337.s001.zip › Supporting Information files/Table S6.doc]

| **Table S6 The miRNA names, primer sequences** | | |
| --- | --- | --- |
| numbers | miRNA names | primer seuqueces（5'-3'） |
| 1 | *dlo-miR156a* | TCCTGACAGAAGAGAGTGAGCAC |
| 2 | *dlo-miR156c* | GATTTGACAACGAGAGAGAGCAC |
| 3 | *dlo-miR159a.1* | GATTTGGATTGAAGGGAGCTCTA |
| 4 | *dlo-miR159a.2* | GCTTTGGATTGAAGGGAGCTTA |
| 5 | *dlo-miR159c* | CTTTGGATTGAAGGGAGTCCTA |
| 6 | *dlo-miR159f* | ACTTGGATTGAAGGGAGCTCTA |
| 7 | *dlo-miR159b** | CGAGCTCCTTGAAGTCCAATAG |
| 8 | *dlo-miR160a* | CTGGCTCCCTGTATGCCA |
| 9 | *dlo-miR166c** | GAATGTTGTCTGGCTCGAGG |
| 10 | *dlo-miR167a* | AGAAGCTGCCAGCATGATCTA |
| 11 | *dlo-miR168a** | ACGCCTTGCATCAACTGAAT |
| 12 | *dlo-miR390a.1* | GCGCTATCCATCCTGAGTTTC |
| 13 | *dlo-miR390a*.1* | GCCGCTATCTATCCTGAGTTTCA |
| 14 | *dlo-miR397a* | CATTGAGTGCAGCGTTGATG |
| 15 | *dlo-miR398b.1* | AAGTTCTCAGGTCGCCCCT |
| 16 | *dlo-miR398b.2* | TGTTCTCAGGTCGCCCC |
| 17 | *dlo-miR398b.3* | GTGTTCTCAGGTCGTCCCTG |
| 18 | *dlo-miR808* | CGAGATCAGTGGGAAAAGAAGAA |
| 19 | *dlo-miR2089*-1* | AGGAGTGGTGAATAGGTACATGAA |
| 20 | *dlo-miR5077* | TTCACGTCGGGTTCACCA |
| 21 | *dlo-miR4a* | ACGGGTTCAAAGGTTGACAGA |
| 22 | *dlo-miR17* | TGCAGCACAAAATACAGTCTGG |
| 23 | *dlo-miR24* | AAATGATTTCGGACCAGGCT |
| 24 | *dlo-miR26* | TGTGAATGATGCGGGAGATAA |
| 25 | *U6 snRNA* | CGATACAGAGAAGATTAGCATGG |
| 26 | *5.8S RNA** | ACGTCTGCCTGGGTGTCACAA |
